# The Cultural Evolution of Structured Languages in an Open‐Ended, Continuous World

**DOI:** 10.1111/cogs.12371

**Published:** 2016-04-07

**Authors:** Jon W. Carr, Kenny Smith, Hannah Cornish, Simon Kirby

**Affiliations:** ^1^ School of Philosophy, Psychology and Language SciencesUniversity of Edinburgh; ^2^ Psychology, School of Natural SciencesUniversity of Stirling

**Keywords:** Categorization, Communication, Compositionality, Cultural evolution, Iterated learning, Language evolution, Sound symbolism

## Abstract

Language maps signals onto meanings through the use of two distinct types of structure. First, the space of meanings is discretized into categories that are shared by all users of the language. Second, the signals employed by the language are compositional: The meaning of the whole is a function of its parts and the way in which those parts are combined. In three iterated learning experiments using a vast, continuous, open‐ended meaning space, we explore the conditions under which both structured categories and structured signals emerge ex nihilo. While previous experiments have been limited to either categorical structure in meanings or compositional structure in signals, these experiments demonstrate that when the meaning space lacks clear preexisting boundaries, more subtle morphological structure that lacks straightforward compositionality—as found in natural languages—may evolve as a solution to joint pressures from learning and communication.

## 1 Introduction

Language facilitates the division of the world into discrete, arbitrary categories (Lupyan, Rakison, & McClelland, 2007). For example, the words *bottle*,* cup*,* flask*,* glass*, and *mug* separate the space of drinking vessels into discrete regions based on such features as shape, material, and function; however, languages differ in the way they discretize our continuous sensory perception of the observable world (Malt, Sloman, & Gennari, 2003). The presence of categorical structure in language reduces an intractable, theoretically infinite set of meanings to a tractable, finite set of words that have the flexibility to handle novel exemplars (Lakoff, 1987). By aligning on a particular system of categorical meaning distinctions, members of a linguistic population can rely on their shared understanding of the structure of the world to successfully communicate.

A second important property of language is its compositional structure: The meaning of a sentence—at multiple levels of analysis—is a function of the meanings of its parts and the way in which those parts are combined. For example, the meanings of *the water is in the cup* and *the cup is in the water* are predictable from the constituent parts (six monomorphemic words) and the word order. In language, compositional structure is a means for optimizing the trade‐off between expressivity (the number of meanings that can be expressed) and compressibility (the degree to which the language can be reduced to atomic units and rules of recombination) (Kirby, Tamariz, Cornish, & Smith, 2015).

This paper focuses on how these two structural properties of language (categorical and compositional structure) can emerge simultaneously through the cultural evolutionary processes that are argued to hold at least some explanatory power in understanding where such structure comes from (e.g., Christiansen & Chater, 2008). Although the cultural evolution of categorical (e.g., Xu, Dowman, & Griffiths, 2013) and compositional (e.g., Kirby, Cornish, & Smith, 2008) structure has previously been demonstrated in isolation, we show here that structured languages can evolve where no categories have been provided by the experimenter a priori. We show this using an *open‐ended* meaning space and the experimental paradigm of *iterated learning*.

### 1.1 Iterated learning

Iterated learning refers to “a process in which an individual acquires a behavior by observing a similar behavior in another individual who acquired it in the same way” (Kirby et al., 2008, p. 10681). For example, an individual learns a language from his or her parents, who themselves learned the language from their own parents. Taking inspiration from earlier computational (e.g., Hurford, 1989; Kirby, 2002; Smith, 2004) and experimental (e.g., Galantucci, 2005; Horner, Whiten, Flynn, & de Waal, 2006; Selten & Warglien, 2007) studies, Kirby et al. (2008) devised an experimental paradigm for studying iterated learning using adult human learners.

The basic design of an iterated learning experiment is as follows. An artificial language (i.e., a mapping between signals and meanings) is generated. In the case of Kirby et al. (2008), this language was a set of 27 randomly generated strings that were mapped onto a fixed set of 27 meanings (three shapes, in one of three colors, moving in one of three distinct patterns). Participants learn this language in a training phase and are then asked to reproduce the language by typing in the corresponding strings for a selection of meanings. The output from this test phase is then taught to a new participant, whose test output is, in turn, taught to another new participant. These experiments typically show that, after several generations, the languages that initially started out as random evolve some form of structure.

The simplest kind of structure that can arise from these experiments is where participants collapse all meaning distinctions. This kind of language (referred to as “degenerate” by Kirby et al., 2015) is highly learnable because a single word can be applied to any meaning. Similarly, systems of structure can arise where the meaning space is collapsed into a small number of categories, each labeled by a distinct word. These kinds of structure represent one way in which languages might adapt to become easier to learn and therefore reliably transmitted. However, while these kinds of language are highly compressible, they are not expressive (see Kirby et al., 2015, for more discussion of this trade‐off).

The second experiment reported by Kirby et al. (2008) implemented a “filtering” system that removed duplicate strings from the training material taught to the next participant in a chain, such that the training language always consisted of a set of unique signals. This modification was intended as an analog of the pressure for expressivity that exists in natural languages. In this experiment, small sets of meaningful, recombinable units emerged corresponding to the dimensions of the meaning space. For example, labels for all blue stimuli began with *l‐* and labels for all stimuli moving in a spiral motion ended with *‐pilu*. By learning a handful of linguistic units and the rules for combining them, participants were able to generate a unique label for any possible meaning combination, including meanings they had not been taught during training.

### 1.2 Continuous meaning spaces

Iterated learning experiments have typically relied on meaning spaces that are discrete, finite, low dimensional, and structured by the experimenter. Kirby (2007) has described such meaning spaces as fixed and monolithic (p. 256). For example, the meaning space used in Kirby et al. (2008), described above, is three dimensional with each dimension (color, shape, and motion) varying over three discrete qualities. To take another example, the space in Smith and Wonnacott (2010) has two discrete dimensions (animal and plurality) for a total of eight meanings.

More recently, iterated learning experiments have been conducted using continuous meaning spaces (see also work with continuous signal spaces by e.g., Verhoef, 2012). Xu et al. (2013) conducted an experiment where participants had to label a continuous color space using between two and six color terms according to condition. The way in which a participant discretized the space was then taught to a new participant in a chain. After 13 generations of cultural transmission, the structure of the space came to resemble the way in which color space is typically structured by languages recorded in the World Color Survey (Kay, Berlin, Maffi, Merrifield, & Cook, 2009). For example, in the three‐term condition, the emergent systems discretized the space into dark, light, and red categories.

Perfors and Navarro (2014) used a meaning space of squares that could vary continuously in terms of color (white to black) and size (small to large). In one condition, there was an abrupt change in the color, such that the stimuli could be categorized into two broad categories (light‐colored squares and dark‐colored squares); in another condition, there was an abrupt change in the size of the squares. Labels for these stimuli were then passed along a transmission chain of learners. In both conditions, the authors found that the structure of the emergent languages came to mirror the structure of the meaning space, primarily making color or size distinctions according to condition.

Silvey, Kirby, and Smith (2013) produced a continuous meaning space by randomly generating four seed polygons and then gradually morphing the polygons into each other, creating a space of 25 stimuli. The space had no obvious internal boundaries; as such, participants showed variation in how they discretized it. The authors also conducted an iterated learning experiment using the same meaning space (Silvey, 2014, Chapter 5). In this experiment, each generation consisted of a pair of participants who communicated about the stimuli using a fixed set of up to 30 words. Over five generations, the category systems that emerged tended to make fewer distinctions and became easier to learn. Furthermore, the category structures became increasingly convex, providing experimental evidence for predictions made by Gärdenfors (2000) about semantic convexity.^1^

### 1.3 Research questions

Two important and related questions arise from prior research into iterated learning. First, to what extent are the general findings supported under more realistic assumptions about meaning? For example, do the results still hold when the meaning space possesses properties that more closely reflect the natural world (e.g., high‐dimensionality, open‐endedness, continuousness)? This question has been partially addressed by the work with continuous meaning spaces described above (see also simulation work by e.g., Laskowski, 2008). The second question that arises is whether iterated learning simply returns the structure prescribed by the experimenter, transferring it from one domain (e.g., predefined categories in the meaning space) to another domain (e.g., the emergent structure in the signals). Xu et al. (2013) address this issue to a certain extent; however, the participants in their experiment are explicitly told how many categories to create—the number of categories does not arise naturally—and the participants are also likely to have strong preconceptions of how to discretize color space based on the color system of their native language (although the authors do address this); furthermore, Xu et al. (2013) do not test for emergent signal structure, since a fixed set of labels is provided. If it is indeed the case that iterated learning experiments simply return structure provided by the experimenter, is it realistic to assume that structured languages can evolve in a context where individuals are not provided with shared categorizations of the observable world?

In this paper, we address these concerns by introducing a novel meaning space of randomly generated triangle stimuli. Like previous work, our meaning space is continuous, but crucially it is also open‐ended: The structure of the space is neither provided by the experimenter nor naturally categorizable; instead it is up to the participants to arbitrarily decide how to categorize the space. In addition, the experiment is set up in such a way that no two generations are tested on or trained on precisely the same stimuli, forcing participants to generalize from the training stimuli to the test stimuli in all cases. Finally, the space of possible stimuli that participants can encounter is vast, forcing participants to adopt a system of categorization. Together, these properties of our meaning space represent more realistic assumptions about the natural world, and by not defining what the meaning dimensions are, we can test whether structure can arise in the signals and in the meaning space simultaneously.

### 1.4 Outline of this paper

This paper reports three artificial language learning experiments that use the paradigm of experimental iterated leaning described above. Experiment 1 (basic transmission) looks at what happens when there is no pressure for expressivity. It therefore provides a baseline for how participants respond to the open‐ended meaning space. The results demonstrate that categories emerge over generational time to discretize the space of possible triangles. Experiment 2 (transmission with an artificial expressivity pressure) explores whether compositional structure can emerge alongside the categorization of the meaning space by implementing an artificial pressure for expressivity. The results of this experiment were negative, suggesting that the second experiment reported by Kirby et al. (2008) may be a special case relating to the discrete meaning space adopted therein. Experiment 3 (transmission with communication) implements a natural expressivity pressure—communication—and shows that sublexical structure can emerge when languages are both learned and used to communicate.

## 2 Experiment 1: Basic transmission

Our first experiment is equivalent to the first experiment reported by Kirby et al. (2008) and looks at what happens when languages are passed along a simple transmission chain with no pressure for expressivity. We had two hypotheses about what would happen over generational time:

1. We expect that the languages will become increasingly easy to learn.
2. We expect to find emergent categories in the meaning space.

These outcomes were expected because the languages should adapt to the cognitive biases of the language users, gradually becoming more learnable. Categories are a way to increase learnability because they constitute a more compressed representation of the meaning space.

### 2.1 Method

The experiment adopted the standard iterated learning paradigm described previously: Participants were arranged into transmission chains in which the output from generation *i* became the input to generation *i* + 1 for a given chain.

#### 2.1.1 Participants

Forty participants (20 female) were recruited at the University of Edinburgh. The median age was 22 years (range: 19–34). Participants were paid £5.50 for participation, and a £20 Amazon voucher was offered as a prize for the best learner. Ethical approval was granted for all experiments reported in this paper according to the procedures of the School of Philosophy, Psychology, and Language Sciences at the University of Edinburgh. All participants provided informed consent and were offered debrief information.

#### 2.1.2 Stimuli

**Figure 1:**
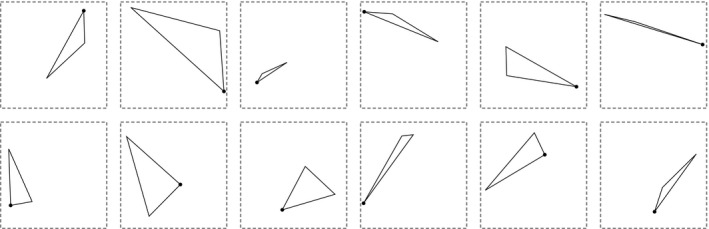
Examples of the triangle stimuli. The stimuli are generated by randomly selecting three points inside a dashed, gray bounding box. One vertex is marked with a black circle.

Participants learned and produced artificial languages that consisted of labels paired with triangles. To generate a triangle stimulus, three points were chosen at random in a 480×480‐pixel space and joined together with black lines (2 pixels wide). The space was enclosed in a 500×500‐pixel dashed, gray bounding box. One vertex (determined randomly) was marked with a black circle with a radius of eight pixels (referred to as the *orienting spot*). Its function is to give the participant some context about which way the triangle is oriented, although this was not explicitly explained to participants. The number of stimuli^2^ that can be generated in this way is $3\left(\begin{array}{c} 480^{2}\\ 3 \end{array}\right)\;\approx \;6\;\times \;10^{15}$. See Fig. 1 for some examples of the triangle stimuli. In this paper, we use the terms *dynamic set* and *static set* to refer to subsets from the set of possible triangles that participants may be exposed to. These terms are explained in greater detail below; for now it suffices to say that a unique dynamic set is generated at every generation (i.e., it changes across participants and generations), while the static set is identical for all participants across all experiments, allowing us to take measurements on a consistent set of stimuli.

The labels used as input to the first generation in a chain were generated by concatenating 2–4 syllables at random. A syllable consisted of a consonant from the set {*d*,* f*,* k*,* m*,* p*,* z*} and a vowel from the set {*a*,* i*,* o*,* u*} (pronounced /ɑ i oʊ u/), yielding 24 possible syllables. The labels used as input to subsequent generations were derived from the output of the previous generation in the chain. We used the MacinTalk speech synthesizer (Alex voice) to produce a synthesized spoken version of each label with primary stress on the penultimate syllable. The use of spoken stimuli, alongside the written stimuli, offers a number of benefits: (a) it makes the task more engaging, (b) if frees participants from having to consider how to pronounce or subvocalize the words, (c) it ensures that all participants hear the words pronounced in the same way, and (d) it ensures that participants still hear the word even if they only pay attention to the triangle stimulus and ignore the written label. When participants introduced new characters, those characters were assigned phonological values consistent with English orthography.

#### 2.1.3 Procedure

**Figure 2:**
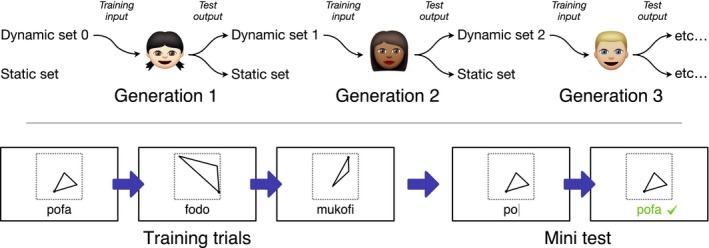
(Top) The participant at generation *i* is trained on a set of triangle stimuli paired with labels (dynamic set *i* − 1). He or she is then tested on two novel sets of triangles: a randomly generated set (dynamic set *i*) and a set that remains constant for all participants (the static set). The labels applied to the dynamic set become the training input to generation *i* + 1. (Bottom) During training, the participant sees a series of three triangles along with their associated labels. One of the three triangles is then presented again, and the participant is prompted to type its associated label. Feedback is then given on whether the answer was correct.

Participants were assigned to one of four chains at random until the chain reached 10 generations. Participants were told that they would be learning the language of the *Flatlanders* (after Abbott, 1884), a fictional life‐form that has many words for triangles. The task was explained to participants in a written brief (see Appendix S1 in the supplementary material), the contents of which were reiterated verbally. The experiment was divided into a training phase followed by a test phase. The training phase involved learning the labels used by the previous participant. The test phase involved providing labels for novel triangles. The experimental procedure is illustrated in Fig. 2 and each phase is explained in the following paragraphs.

During training, participants learned the labels that the previous participant had applied to the 48 triangles in his or her dynamic set (i.e., the unique set of stimuli generated for the previous participant's test phase). Each training trial lasted 5 s. On each trial, the triangle was presented first, and its associated label appeared below it after a 1 s delay to ensure that both stimuli were attended to. The synthesized form of the label was played through headphones at the same time as the presentation of the written form. Training was done in three blocks. In each block, the participant was exposed to the 48 items in a randomized order for a total of 144 trials. After every third trial (i.e., 16 times per block, 48 times overall), the participant was shown one of the previous three triangle stimuli again and prompted to type its label. We refer to this as a *mini test*. Over the course of training, each of the 48 items was mini‐tested once. Feedback on each mini test was given in the form of a green checkmark or a red cross according to whether the participant answered correctly. If the answer was incorrect, the correct answer was shown. The mini tests were intended as a means for holding the participant's attention during the training phase.

In the test phase, participants were exposed to 96 triangle stimuli, none of which they had seen during training, and were prompted to type the associated label for each one. The 96 stimuli consisted of the 48 stimuli in a newly generated dynamic set (which would go on to become the training material for the subsequent participant in the chain) and the 48 stimuli in the static set (in randomized order). The presentation of these two sets was interleaved. The static set comprised the same set of triangles across all participants in all experiments, allowing us to take measurements on a consistent set of stimuli. No feedback was provided during the test phase, since there is no right or wrong answer.

### 2.2 Results

**Figure 3:**
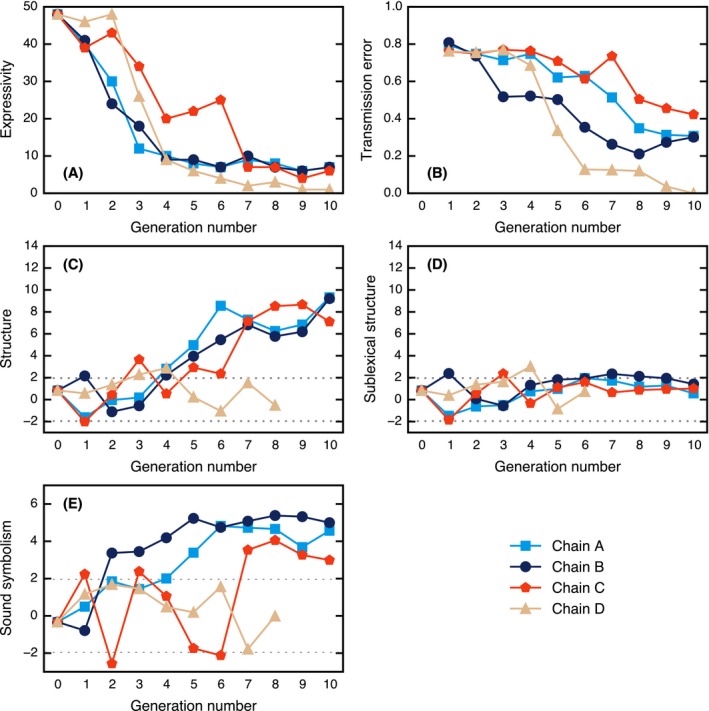
Results of Experiment 1. (A) Expressivity: number of unique strings in the static set. (B) Levels of transmission error. (C) Levels of general structure. (D) Levels of sublexical structure. (E) Levels of shape‐based sound symbolism. The dotted lines in (C), (D), and (E) give the upper and lower 95% significance levels; points lying outside of this interval are unlikely to be explained by chance. Some data points at the end of Chain D are undefined due to the small number of unique strings.

The results for Experiment 1 are shown in Fig. 3 and are discussed in the following sections. The raw data and analysis are available from https://github.com/jwcarr/flatlanders.

#### 2.2.1 Loss of expressivity

We can estimate how expressive a language is by looking at the number of words it contains. A language with more words is potentially capable of making more meaning distinctions. In the initial Generation‐0 input, 48 unique strings were used to label the static set, but by Generation 10, this number decreased to 6 or 7, and in Chain D, a single word, *mika*, was used to describe all triangles. These results are shown in Fig. 3A. Page's test (Page, 1963) revealed that this decrease in the number of unique labels was significant (*L* = 1,993, *m* = 4, *n* = 11, *p* < .001). These results show that the languages are becoming less expressive over time.

#### 2.2.2 Increase in learnability

We expected to find that the languages would become increasingly learnable over time. If a language is easy to learn, a participant's output language should more faithfully reproduce the rules of the input language. In other words, we would expect to find a decrease in intergenerational transmission error over time. Intergenerational transmission error was measured by taking the mean normalized Levenshtein edit‐distance^3^ (Levenshtein, 1966) between the strings used to describe items in the static set at generation *i* and the corresponding strings at generation *i* − 1:

(1) $$\frac{1}{48}\sum_{m=1}^{48}\frac{\text{LD}(s_{i}^{m},s_{i-1}^{m})}{\text{max}[\text{len}\left(s_{i}^{m}\right),\text{len}\left(s_{i-1}^{m}\right)]},$$

where LD gives the Levenshtein edit‐distance, *s* is a string, and *m* is a meaning from the static set of 48 items. This measure of error is expressed in [0, 1], where 0 is perfect alignment between consecutive generations. The results for transmission error are shown in Fig. 3B. Page's test revealed that the decrease in transmission error was significant (*L* = 1,514, *m* = 4, *n* = 10, *p* < .001), suggesting that the languages are becoming easier to learn over time. Although transmission error may appear quite high by the final generation, this should not be surprising, since a score of 0 requires not only that consecutive participants label the categories in the same way, but also that they infer the same category boundaries; in natural languages, however, the boundaries between categories are known to be fuzzy (Rosch, 1973).

#### 2.2.3 Emergence of structure

Although the languages became less expressive, we expected to find that the words would increasingly be used to categorize the space systematically. In a systematic language, we would expect to find that similar labels refer to similar meanings, while dissimilar labels refer to dissimilar meanings. Thus, to measure how structured the system is, we correlate the dissimilarity between pairs of strings with the dissimilarity between pairs of triangles for all *n*(*n* − 1)/2 pairs. The normalized Levenshtein edit‐distance was used as a measure of dissimilarity between strings. To measure the dissimilarity between triangles, we conducted a separate experiment in which naïve participants were asked to rate the dissimilarity between pairs of triangles (see Appendix A for full details of this experiment and Appendix S2 in the supplementary material for an alternative geometric approach). Following previous studies (e.g., Kirby et al., 2008, 2015), the distance matrices for string dissimilarity and triangle dissimilarity were correlated using the Mantel test (Mantel, 1967), since the distances are not independent of each other making standard parametric statistics unsuitable. The test compares the Pearson correlation for the veridical signal–meaning mapping against a distribution of Pearson correlations for permutations of the mapping, yielding a standard score (*z*‐score). The results of this analysis are presented in Fig. 3C. The last two generations of Chain D are undefined under this measure because there is only one word in the language. The plot shows that structure is emerging in all chains with the exception of Chain D. Page's test revealed a significant increase in structure (*L* = 1472, *m* = 3, *n* = 11, *p* < .001; excluding Chain D due to missing data points).

However, this measure of structure cannot discriminate between category structure and string‐internal structure (e.g., compositionality). To test if structure was present inside the signals, a modification was made to the measure: Rather than randomize the mapping between signals and meanings, we randomize the mapping between the category labels (i.e., the unique set of words in the language) and the sets of triangles they map onto, such that the set of triangles labeled by a given word remains intact but the labels for each category are randomly shuffled. Under this randomization method, any categorical structure in the language remains present in the permuted mappings, so a high *z*‐score indicates that there must be additional structure present inside the strings themselves. The results from this alternative approach are shown in Fig. 3D, where the majority of data points are below the upper 95% significance level, suggesting that there is no string‐internal structure in the languages of this experiment.

**Figure 4:**
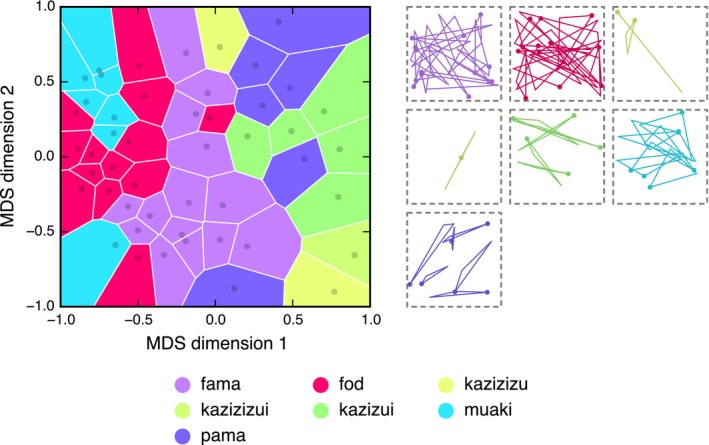
Categorical structure of the meaning space at Generation 10 in Chain A. The plot on the left shows how the meaning space is discretized by the words in the language: Similarity in position represents similarity in meaning; similarity in color represents similarity in word form. On the right, all triangles in the static set are grouped by the word used to describe them (presented in the same order as the legend). Refer to the main text for a full description and interpretation of this figure.

To visualize the categories, the pairwise dissimilarity ratings (obtained from the naïve raters; Appendix A) were passed through a multidimensional scaling (MDS) algorithm, producing a two‐dimensional representation of the meaning space.^4^ MDS finds an arrangement of items in a metric space that best preserves the distances known to exist between those items (see e.g., Borg & Groenen, 2005). The MDS solution is shown in the plot in Fig. 4. Each dark dot represents one of the triangles in the static set; triangles that are close together in this space were rated to be similar, and triangles that are far apart were rated to be dissimilar. Although the dimensions of the space are somewhat abstract, the *x*‐axis appears to correspond to shape, while the *y*‐axis appears to have some correspondence with size. The space is partitioned into 48 Voronoi cells—one cell for each triangle in the static set. Each cell encompasses all points in the space that lie closer to the associated triangle than to any other triangle from the static set. In other words, each Voronoi cell delimits the space of triangles that would have been labeled with the associated string under the assumption that each item is a prototypical member of a convex category (Gärdenfors, 2000).

Color is used in Fig. 4 to show information about the state of the language at Generation 10 in Chain A; similarity in color indicates similarity in word form. To determine a color for each word, we computed the pairwise Levenshtein edit‐distances between the seven words in this particular language and derived a two‐dimensional MDS solution centered on the origin. The Cartesian coordinates in this MDS space were converted to polar coordinates and then mapped into HSV (hue, saturation, intensity value) colorimetric space: The angular coordinate was mapped to hue and the radial coordinate (scaled in [.5, 1] to avoid overly dark colors) was mapped to saturation; the intensity value was held constant at 1 (see Lespinats & Fertil, 2011, for a full description of this method). The seven words are given in the legend alongside their assigned colors. Each Voronoi cell is colored according to the word that was used to describe its associated triangle, making it possible to see how the space is discretized by the words. The plot is a visual approximation of the measure of structure described above: In a structured language, similar colors will cluster into similar regions, while in an unstructured language, colors will be randomly distributed across the space. The images to the right of the plot show all triangle stimuli in the static set grouped and colored according to the word that was used to describe them. Note that Fig. 4 combines two data sets: The structure of the meaning space is determined by the naïve raters, while the color coding is determined by how the participant at Generation 10 in Chain A labeled the triangles. Figures for all generations in all chains can be found in Appendix S3 in the supplementary material.

Fig. 4 clearly shows that the language divides the meaning space into around five categorical regions. The center of the space (medium thin triangles) is occupied by the word *fama* (light purple), with the similar word *pama* (dark purple) branching off into the top‐right corner (smaller thin triangles). The *kazi‐* forms (*kazizizu*,* kazizizui*, and *kazizui*; yellow–green) occupy the right‐hand side of the plot and represent the extremely thin triangles. *Muaki* (blue) mostly occupies the top left (smaller open triangles), and *fod* (pink) occupies the center left (larger open triangles). With some exceptions, the five main categories tend to form single, contiguous regions (e.g., it is possible to travel between any two examples of a *fama* without leaving the *fama* region), although the regions do not appear to be convex (it is not always possible to travel in a straight line without passing through another category). It is important to note, however, that the Voronoi tesselation of MDS space only offers a two‐dimensional model of participants’ underlying conceptual representations of the triangles and linguistic categories; the plots should therefore not be taken as a reliable source of information about the precise structuring of the meaning space.

#### 2.2.4 The rise of sound‐symbolic languages

Sound symbolism describes the phenomenon where a unit of sound goes “beyond its linguistic function as a contrastive, non‐meaning‐bearing unit, to directly express some kind of meaning” (Nuckolls, 1999, p. 228). Although we did not initially set out to test for the emergence of sound‐symbolic languages, it appeared that such patterning might be present. For example, the word *kiki* (the same word used in the classic bouba/kiki experiments; Köhler, 1929) arose independently in several chains (Chains C and D in this experiment and Chains E, G, and H in Experiment 2) to describe very thin or small triangles. To explore the emergence of shape‐based sound symbolism, we hypothesized that the extent to which each triangle was thin vs. equilateral would be correlated with the presence of phonemes associated with pointy vs. round stimuli (following e.g., Köhler, 1929; Kovic, Plunkett, & Westermann, 2010; Maurer, Pathman, & Mondloch, 2006). The “equilateralness” of a triangle (a proxy for shape) was calculated as

(2) $$\frac{a}{p^{2}/\left(12\sqrt{3}\right)},$$

where *a* is the triangle's area and *p* is its perimeter.^5^ To measure the “roundedness” of a string, we used the sound‐symbolic correspondences described by Ahlner and Zlatev 2010, p. 310) to divide all phonemes that occurred into three categories: “round” phonemes /b d g l m n oʊ  Ɔ u/, which received a score of +1, “pointy” phonemes /k p t eɪ i/, which received a score of −1, and all other phonemes, which received a score of 0. We then correlated the total roundedness of the strings with the equilateralness of the corresponding triangles and compared this correlation to a distribution of correlations for permutations of the mapping between signal and meaning to arrive at a standardized measure of shape‐based sound symbolism. The results are shown in Fig. 3E; by the final generations, there are significant levels of shape‐based sound symbolism in chains A, B, and C.

The same analysis was conducted for size‐based sound symbolism using the centroid size^6^ as a measure of a triangle's size. This measure is uncorrelated with the triangle's shape (Bookstein, 1991, p. 97), which is particularly important given the great amount of overlap in phonemes associated with both shape and size. Specifically, the “bigness” of a string was measured based on the phonemes listed in Thompson and Estes 2011, p. 2396): The “big” phonemes /b d g l m w  ɑ oʊ  Ɔ u/ received a score of  +1 and the “small” phonemes /k p t eɪ i/ received a score of −1. While there was an effect in some later generations, the results were quite weak. Given the lack of a strong effect for size, only the shape‐based sound symbolism results are reported in this paper.

#### 2.2.5 Summary of Experiment 1

The results for Experiment 1 suggest that categorical structure emerges in the languages. In Chains A, B, and C, the space of possible triangles was gradually divided into a small number of arbitrary categories that varied across chains. In Chain D, a single word came to stand for all triangles, which is itself a form of categorical structure—in everyday English, for example, all three‐sided, two‐dimensional figures can be categorized under the single word *triangle*. The small number of words that emerged in the languages by the final generations mirrors the underspecification found in the first experiment of Kirby et al. (2008). Categories allow for languages that are more compressed and, as such, more learnable. For example, the language depicted in Fig. 4 can be minimally represented by seven words, but it is presumably capable of describing any of the $6\;\times \;10^{15}$ triangles that could have been generated. However, highly compressed languages are not necessarily useful in the context of language use, where it is important to be able to disambiguate one referent from a set of referents (see Kemp & Regier, 2012, for an example of this trade‐off in the context of kinship categories). To test whether more expressive languages could evolve under this unstructured, open‐ended meaning space, we conducted two additional experiments that include expressivity pressures.

## 3 Experiment 2: Transmission with an artificial expressivity pressure

Our second experiment tests whether artificially forcing participants to use expressive languages results in compositional structure as a solution to maintaining both diversity of forms and compressible (and therefore learnable) languages. We had three hypotheses:

1. We expect that the languages will become increasingly easy to learn.
2. We expect to find emergent categories in the meaning space.
3. We expect to find emergent structure in the signals (e.g., compositionality).

The addition of Hypothesis 3 to the two hypotheses of Experiment 1 was motivated by Kirby et al. (2008), whose second experiment showed that forcing languages to remain expressive results in emergent compositional structure. In our experiment, participants could, for example, use a system where the first syllable (*a*,* b*, or *c*) denotes three sizes, the second syllable (*d* or *e*) denotes broad or thin, and the third syllable (*f*,* g*,* h*, or *i*) denotes the quadrant that the triangle is primarily located in. In this example, participants would only need to learn nine linguistic units (syllables *a*–*i*) and the rules for combining them but would be able to generate 3 × 2 × 4 = 24 distinct words, providing referential precision at minimal cost in terms of the number of label components to be learned.

### 3.1 Method

#### 3.1.1 Participants

Forty participants (25 female), none of whom took part in Experiment 1, were recruited at the University of Edinburgh. The median age was 22 years (range: 18–50). Participants were paid £5.50 for participation, and a £20 Amazon voucher was awarded to the best learner.

#### 3.1.2 Procedure

The procedure was identical to Experiment 1, except that participants could not use the same string more than three times to label test items from the dynamic set (i.e., every other test trial). We did not impose this limitation on the static set because only the dynamic set can lead to a runaway loss of expressivity, since the way in which this set was labeled would be passed to the next generation. The advantage of this approach is that participants will only encounter the expressivity pressure in half of trials. The disadvantage is that the static set may not be entirely representative of how the participant responded in the dynamic set. In dynamic set trials, upon attempting to enter a word that had previously been used three times, the participant was presented with the message “You've used this word too often. Please use another word.” An additional sentence was added to the brief to explain that this could happen (see Appendix S1 in the supplementary material). This modification to the test procedure forces the languages to remain expressive, since the output languages passed to the next generation must contain a minimum of 48 / 3 = 16 unique strings.

### 3.2 Results

**Figure 5:**
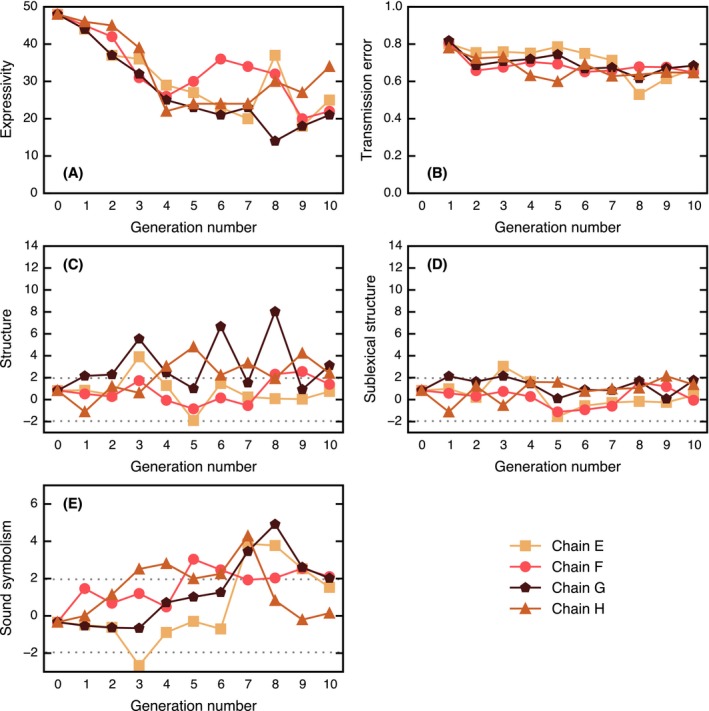
Results of Experiment 2. (A) Expressivity: number of unique strings in the static set. (B) Levels of transmission error. (C) Levels of general structure. (D) Levels of sublexical structure. (E) Levels of shape‐based sound symbolism. The dotted lines in (C), (D), and (E) give the upper and lower 95% significance levels; points lying outside of this interval are unlikely to be explained by chance.

The results of Experiment 2 are shown in Fig. 5 and are discussed in the following sections.

#### 3.2.1 Expressivity

The number of unique strings used to label items in the dynamic set was not able to collapse so dramatically. Although the pressure was only applied to the dynamic set, the number of unique strings in the static set also remained high (as shown in Fig. 5A). The languages thus remain more expressive than Experiment 1.

#### 3.2.2 Learnability

Fig. 5B shows that intergenerational transmission error in Experiment 2 remained relatively static. Nevertheless, the results do show a significant decrease (*L* = 1,415, *m* = 4, *n* = 10, *p* < .001) from an average of 80% error at Generation 1 down to an average of 66% error at Generation 10.

#### 3.2.3 Structure

**Figure 6:**
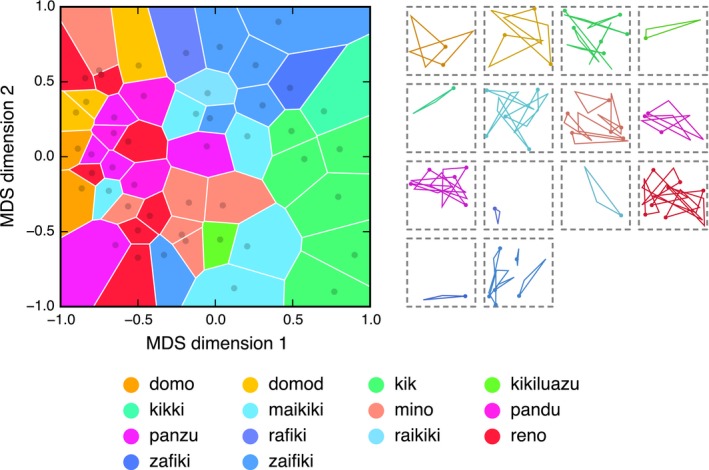
Categorical structure of the meaning space at Generation 8 in Chain G. The plot on the left shows how the meaning space is discretized by the words in the language: Similarity in position represents similarity in meaning; similarity in color represents similarity in word form. On the right, all triangles in the static set are grouped by the word used to describe them.

Although the languages in Experiment 2 are more expressive, this did not translate into increased levels of structure. Like Experiment 1, there is no evidence for sublexical structure (Fig. 5D); however, levels of general structure are also low (Fig. 5C), with only Chains G and H showing marginal, albeit fragile, levels of structure. Fig. 6 shows the state of the language at Generation 8 in Chain G. In this example, which was the most structured language to emerge, there is a clear tendency for similar labels to cluster together. For example, labels colored green cluster down the right‐hand side, dark blues in the top left, orange–yellows on the left‐hand side, and so forth. However, the structure of the space is not as clear cut as in the case of Experiment 1, partly due to the increased number of words. In general, however, strong levels of categorical structure did not develop in this experiment (as indicated by Fig. 5C), and it seems that the participants continue to make a small number of categorical distinctions by using similar (but not necessarily identical) strings to label each category. For example, although the language shown in Fig. 6 uses 14 labels, there appear to be five broad categories (colored blue/cyan, green, magenta, orange/yellow, red/salmon; this is not simply an artifact of color perception as these five broad categories are also clear from the strings themselves).

#### 3.2.4 Sound symbolism

Like Experiment 1, there are significant levels of shape‐based sound symbolism emerging in some of the later generations (Fig. 5E), although the effect tends to be weaker.

#### 3.2.5 Summary of Experiment 2

Placing a limit on the number of times a particular word could be reused allowed the languages to remain expressive. However, this did not translate into compositional structure as hypothesized. In fact, the substantial variation in the languages prevented many of the participants from stabilizing on a set of reliable categories. This result is at odds with the second experiment reported by Kirby et al. (2008), where an artificial pressure was sufficient to give rise to compositional languages. While there are many possible explanations for this, one possibility is that an artificial pressure for expressivity is only sufficient in the artificial case of a small, discrete, structured meaning space.

## 4 Experiment 3: Transmission with communication

The restriction imposed on Experiment 2 was artificial; although participants had to remain expressive, there was no natural reason to use a large number of distinct strings. In our final experiment, we replaced the artificial expressivity pressure with a more ecologically valid pressure: At each generation, two participants must use the language to communicate with each other. Communication introduces a natural pressure for expressivity because, in order to maximize their communicative success, a pair of participants will need a language that is well‐adapted to the discrimination of referents in a world of triangles. Our hypotheses were identical to those of Experiment 2.

### 4.1 Method

#### 4.1.1 Participants

Eighty participants (63 female) were recruited at the University of Edinburgh, none of whom took part in Experiments 1 or 2. The median age was 21 years (range: 18–37). Participants were paid £8.50 for participation. The pair of participants who were most successful at communicating were both awarded a £20 Amazon voucher to encourage participants to be as communicative as possible with their partners.

#### 4.1.2 Procedure

**Figure 7:**
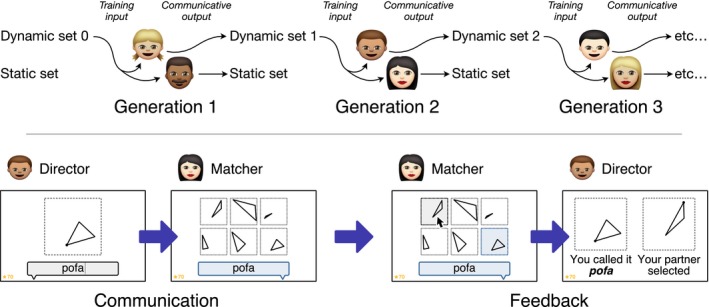
(Top) The participants at generation *i* are individually trained on dynamic set *i* − 1. They then communicate about two novel sets of triangles: a randomly generated set (dynamic set *i*) and a set that remains constant for all participants (the static set). The labels applied to the dynamic set become the training input to generation *i* + 1. (Bottom) During communication, the director is shown a triangle and is prompted to type a label to describe it. The label is then displayed on the matcher's screen along with an array of six triangles to choose from. The matcher's task is to click on the triangle that his or her partner is trying to communicate. As feedback, both participants see the target triangle and the selected triangle.

The task was explained to participants in a written brief (see Appendix S1 in the supplementary material), the contents of which were reiterated verbally. The procedure followed the same communication game paradigm introduced in other iterated learning experiments (e.g., Kirby et al., 2015; Winters, Kirby, & Smith, 2015); this is illustrated in Fig. 7. Sitting in separate booths, a pair of participants completed the same training regimen used in Experiments 1 and 2. The training material presented to the two participants was identical and was derived from the dynamic set of the previous generation. Once both participants had completed training, they entered a communication game in which they took turns to play the role of director and matcher. The director was shown a triangle stimulus on his or her screen and was asked to describe that triangle to his or her partner. This label was then displayed on the matcher's screen along with six triangles to choose from (the context array). The context array contained the target triangle (in randomized position) and five randomly generated distractors. The matcher's task was to click on the triangle that his or her partner was trying to communicate. The director and matcher were provided with full feedback: After making a selection, the correct target in the context array was highlighted in blue, and the director was shown the triangle that the matcher had selected alongside the correct target. The participants were jointly awarded 10 points for each correct match; the number of points accumulated was shown in the bottom left corner of both screens throughout the communication game.

One of the participants (determined randomly) labeled the dynamic set and the other labeled the static set for a total of 96 communication trials. Like the previous experiments, the dynamic and static sets were labeled in alternation as the pair of participants swapped roles. This approach means that the subsequent generation was exposed to input from one cultural parent (the participant who labeled the dynamic set); the disadvantage is that the static set is only representative of the participant who labeled that set.

### 4.2 Results

**Figure 8:**
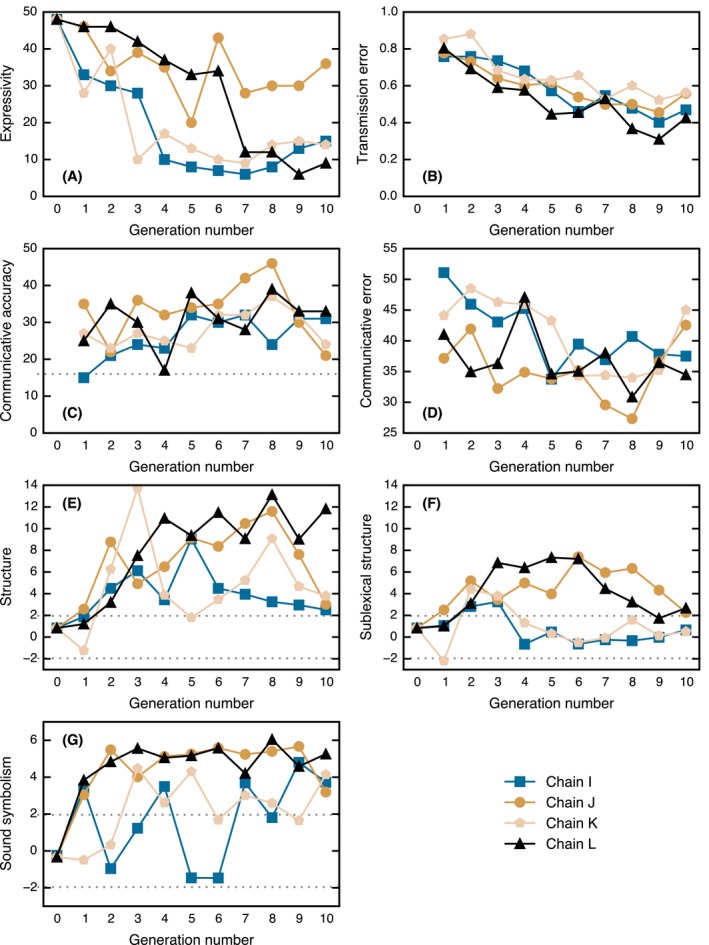
Results of Experiment 3. (A) Expressivity: number of unique strings in the static set. (B) Levels of transmission error. (C) Number of correct trials (the dotted line indicates chance level). (D) Communicative error. (E) General structure. (F) Sublexical structure. (G) Shape‐based sound symbolism. The dotted lines in (E), (F), and (G) give the upper and lower 95% significance levels; points lying outside of this interval are unlikely to be explained by chance.

The results of Experiment 3 are shown in Fig. 8 and are discussed in the following sections.

#### 4.2.1 Expressivity

The expressivity results are shown in Fig. 8A. The number of unique strings is generally greater than that observed in Experiment 1, and the number of unique strings in Chain J and the first half of Chain L is comparable to Experiment 2.

#### 4.2.2 Learnability

The results for transmission error are shown in Fig. 8B. There is a significant decrease (*L* = 1,503, *m* = 4, *n* = 10, *p* < .001) from an average of 80% error at Generation 1 down to an average of 50% error at Generation 10.

#### 4.2.3 Communicative accuracy

Fig. 8C shows the number of times the communicating pair correctly identified the target triangle out of 96 trials. The chance level of accuracy under this measure is 96 / 6 = 16 (indicated by the dotted line). All but one of the pairs scored above chance. There was a significant increase (*L* = 1,321.5, *m* = 4, *n* = 10, *p* = .021), with later generations tending to make more correct matches. Fig. 8D shows a more fine‐grained measure of communicative accuracy: the total dissimilarity between the selected triangle and the target triangle for all incorrect responses (dissimilarity scores were collected in a separate experiment; see Appendix B). This gives a measure of the amount of communicative error at each generation. There was a significant decrease (*L* = 1,356, *m* = 4, *n* = 10, *p* = .004), which again indicates that later generations communicate more accurately. Nevertheless, levels of communicative accuracy were quite low. The pair of participants with the highest score was Generation 8 in Chain J (46 correct trials). That all participants got less than half of trials correct indicates that the task was particularly difficult and that there may be a ceiling on how well participants can perform, given the amount of training they receive and the length of time they communicate for. It is also likely that a pair of participants will not infer identical category boundaries, resulting in difficulty classifying nonprototypical members of a given category.

#### 4.2.4 Emergence of sublexical structure

**Figure 9:**
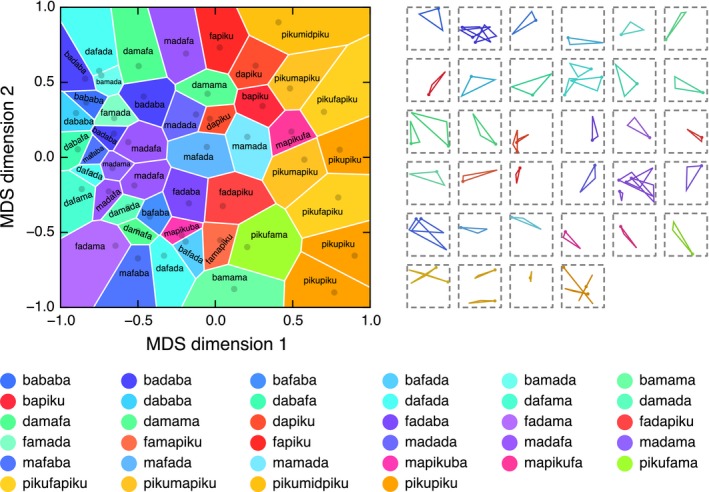
Categorical structure of the meaning space at Generation 6 in Chain L. The plot on the left shows how the meaning space is discretized by the words in the language: Similarity in position represents similarity in meaning; similarity in color represents similarity in word form. On the right, all triangles in the static set are grouped by the word used to describe them.

The results for general structure are shown in Fig. 8E. Structure emerged very rapidly and remained high over the generations (*L* = 1,755, *m* = 4, *n* = 11, *p* = .007). Furthermore, Fig. 8F reveals that sublexical structure is present in Chains J and L, peaking at around Generation 6. To take one example, the language at Generation 6 in Chain L comprises five main units: *ba*,* da*,* fa*,* ma*, and *piku*. In nearly all cases, two or three of these units will be combined together to create a word. The way in which the words map onto the meaning space is shown in Fig. 9. Due to the large number of words, each Voronoi cell in the plot has been labeled to make the system easier to comprehend.

The pattern that immediately stands out is the tendency for labels represented by orange–yellow to cluster on the right‐hand side of the plot. These triangles are labeled with words containing *piku* in initial and final position. There is also a clustering of reds and pinks corresponding to words containing *piku* in second or final position only. When *piku* occurs only once in the word, it usually indicates triangles that are small or somewhat thin (e.g., *bapiku*,* dapiku*,* fapiku*,* mapikuba*,* fadapiku*). When a word begins and ends with *piku*, it will usually refer to a very thin triangle with little area (e.g., *pikufapiku*,* pikumapiku*,* pikumidpiku*). In fact, the three thinnest triangles are simply labeled *pikupiku*. These results suggest that reduplication, a common cross‐linguistic phenomenon (Moravcsik, 1978), may play in role in intensifying meaning, perhaps through an iconic principle (double the *piku* corresponds to double the thinness; cf. Regier, 1998). Words with *da* in first position usually refer to triangles which are large and open (e.g., *dababa*,* dabafa*,* damafa*). However, when *da* occurs in second position, it often indicates that the triangle lies on the right‐hand side of the bounding box (e.g., *fadaba*,* fadama*,* fadapiku*,* madada*,* madama*). Finally, words with *ma* in first position often correspond to triangles whose orienting spots point to the top‐left corner of the bounding box (e.g., *madafa*,* mafaba*,* mamada*,* mapikufa*). However, these patterns are probabilistic; for each rule, exceptions can be identified.

Perhaps more interestingly, in many words, there appear to be meaningful subparts combined with nonmeaningful subparts. For example, the meanings of *fa* and *ma* in the words *pikufapiku* and *pikumapiku* are unclear. These subparts may be morphological residue like that found in cranberry morphs. Cranberry morphs are a class of morpheme that, for a given language, occur in only one word; as such, it is difficult to assign meaning to them without circular reference back to the word itself, calling into question the meaning of the term *morpheme* (traditionally, the smallest unit of meaning; see Aronoff, 1976, Chapter 2 for discussion of this issue). The classic example is the *cran* in the word *cranberry*, which has no independent meaning; instead it serves to distinguish cranberries from other types of berry. Similarly, the *fa* and *ma* in *pikufapiku* and *pikumapiku* may express the idea, “I'm of the type *piku...piku*, but slightly different in a way I won’t explicitly specify.” For instance, the *fa* type of *piku...piku* is slightly longer and thinner than the *ma* type, but this correspondence does not appear to be productive across the language as a whole.

#### 4.2.5 Sound symbolism

Fig. 8G shows levels of shape‐based sound symbolism, which are very strong and tend to emerge early in the chains. This is likely because the pair of participants can rely on a shared, implicit understanding of common sound‐symbolic patterns to more accurately communicate with each other.

#### 4.2.6 Summary of Experiment 3

Introducing communication created a natural pressure for participants to be expressive. Expressivity remained higher than Experiment 1 and comparable to Experiment 2. Despite this, the learnability of the languages also remained high. Participants in at least two of the chains managed the pressures for expressivity and learnability by utilizing string‐internal structure that leverages the structure in the meaning space and sound‐symbolic associations. Thus, in this experiment, where there was a pressure to maintain the diversity of signals due to the natural pressure from expressivity in addition to the pressure for learnability associated with transmission, sublexical structure emerged in addition to the general categorical structure observed in the previous experiments.

## 5 Discussion

In the Introduction, we claimed that our meaning space is a useful model of the natural world because the space of triangles is vast, continuous, and open‐ended, properties that are present in objects that occur in the real world. For example, the vast set of items referred to by the English word *cup* forms a conceptual category that has fuzzy boundaries with neighboring concepts, such as *bowl*,* glass*, and *pitcher* (Labov, 1973). The dimensions of the conceptual space in which cups are represented may be either discrete (e.g., the presence or absence of a handle) or continuous (e.g., its size or shape). Similarly, our space of triangles potentially has both discrete (e.g., the quadrant in which the triangle is located) and continuous (e.g., the size or rotation of the triangle) dimensions with boundaries that are not well defined. Furthermore, our participants are unlikely to have strong preconceptions about how the space of triangles should be discretized. While geometrical terminology exists to describe the shape of triangles (equilateral, isosceles, and scalene) and their angles (acute, obtuse, and right‐angled), these terms are not particularly useful in the context of our experimental paradigm, since they discretize the space of triangles based on artificial mathematical properties rather than naturally perceived features.

In Experiment 1, the languages that emerged discretized the meaning space into a small number of categories. Although the precise boundaries between categories varied from one chain to the next, the categories typically encoded the shape and size of the triangles; other features that could have been encoded—location or rotation in the plane—tended to be disregarded by the participants (see also Section 2 of Appendix S2 in the supplementary material). In fact, the naïve raters broadly responded to the space in the same way, rating the dissimilarity between triangles based on their shape and size properties (as evidenced by the dimensions of the MDS space). This is congruent with Landau, Smith, and Jones (1988), who showed that, when learning words, both children and adults are biased toward the shape of stimuli over their color, texture, or size. The process of collapsing categorical distinctions was taken to the extreme in one of the chains where a single word was used for all triangles by the final two generations. The process of collapsing categories is a valid strategy for maximizing compressibility (and therefore learnability), but the emergent languages in Experiment 1 were not expressive and would therefore be ill‐suited to a world where one needed to reliably discriminate referents.

In Experiment 2, we placed a limit on the number of times a word could be reused, imposing an artificial expressivity pressure on the languages. This was intended to be equivalent to the pressure imposed in Kirby et al.'s (2008) second experiment. While the number of unique strings remained high in Experiment 2, there was no evidence of the sublexical structure one would expect to find in a compositional system. In fact, the large amount of variation within each language even prevented stabilization on a set of categories in the meaning space. This result is strikingly different from the results reported by Kirby et al. (2008), who observed robust compositional structure under such a pressure. One explanation for this could be that, when the experimenter provides participants with a structured meaning space with unambiguous internal boundaries, single participants can simply transfer part of the meaning space structure onto the signals, cumulatively giving rise to compositional systems over generational time. In contrast, when participants are presented with an unstructured meaning space, as is the case here, the process of deriving structured signals becomes nontrivial. That being said, the artificial pressure used here is slightly different from that used by Kirby et al. (2008): The pressure involves direct instruction to participants—asking them to use different words when an arbitrary limit is reached—and does not maintain a one‐to‐one mapping between signal and meaning (a signal can map to up to three meanings in this experiment). The effects of such subtle differences are unclear and could be the subject of future work.

In Experiment 3, we added communication, which acts as a natural pressure for expressivity. In this experiment, each generation consisted of communicating participants who had the shared goal of maximizing their communicative accuracy. To achieve this, a language would be required that could encode a sufficient number of feature distinctions in order for the matching participant to correctly determine the target triangle. Like Experiment 2, expressivity remained high, but, unlike Experiment 2, the learnability of the languages also remained comparatively high and our measure of structure revealed that string‐internal structure was present in two of the four chains. Thus, in this experiment, where there was a natural pressure to maintain a diverse set of signals, sublexical structure emerged in addition to the categorical structure observed in Experiment 1.

Nevertheless, it is difficult to describe the emergent sublexical structure as compositional, at least in terms of how compositionality is traditionally defined. A standard, theory‐neutral definition of compositionality states that, “the meaning of a complex expression is determined by its structure and the meanings of its constituents” (Szabó, 2013). However, in our qualitative analysis of the emergent languages, it proved difficult to write simple grammars that could describe how to create composite strings with composite meanings because many of the mappings between form and meaning were highly probabilistic. In addition, in the exit questionnaire, many of our participants were unable to describe how the languages worked, suggesting instead that there were weak statistical tendencies in how form mapped onto meaning; one participant (Chain I, Generation 8, Subject A) remarked, “I think we had vague ideas of the template for each word, but we were pretty inconsistent.”

However, this is precisely how the lexicons of natural languages work. While polymorphemic words are compositional (either through inflection, *washed* = *wash* + *‐ed*, or derivation, *happiness* = *happy* + *‐ness*), monomorphemic words cannot be decomposed into smaller meaningful units. Furthermore, the extent to which polymorphemic words are compositional is also questionable. For example, Aronoff (1976, 2007) takes the view that lexemes, even polymorphemic ones, are largely idiosyncratic. Sentences need to be highly compositional to provide language with its productivity, and the production of sentences is certainly a generative process, leading to combinations of words that have never been uttered before (although cf. Wray & Perkins, 2000). In contrast, the lexicon is stored in memory and many polymorphemic words have idiosyncratic meanings that have drifted from the sum of the parts from which they were originally derived. Aronoff therefore views polymorphemic lexemes as being only weakly compositional. While Aronoff's position may be a radical alternative to the classic view, it provides an alternative perspective on compositionality (or lack thereof) at the level of the lexeme.

The second linguistic property relevant to our results is de Saussure's (1959) *arbitrariness of the sign*, which states that the relationship between form and meaning is arbitrary and established only by convention among language users. In the context of language evolution, the importance of the arbitrariness of the sign was further solidified by Hockett (1960), who counted it among the design features of language. However, there are notable exceptions to this principle, which Cuskley and Kirby (2013) break down into conventional and sensory sound symbolism.^7^

Conventional sound symbolism refers to correspondences between signal and meaning that are set up by the historical relatedness of words. Such correspondences have been shown to contribute to the overall systematicity of natural languages using corpus‐analytical techniques in both English (Monaghan, Shillcock, Christiansen, & Kirby, 2014) and Spanish (Tamariz, 2008). One example of this, which seems likely to contribute to such statistical correspondences, is phonesthesia—the phenomenon where monomorphemic words contain correspondences between sound and meaning. For example, English words beginning with *sn‐* often have meanings relating to the nose (e.g., *sneeze*,* sniff*,* snore*,* snout*, etc.). Such words may possess shared etymologies that are obfuscated by the current state of the language and/or may be adopted precisely because of the correspondences they share with preexisting words in the lexicon. Bergen (2004) and Hutchins (1998) have shown in psycholinguistic experiments that the English phonesthemes have a psychological reality in the minds of native speakers, suggesting that they should be considered in a similar light to regular morphemes (see Kwon & Round, 2015, for some discussion).

The second type, sensory sound symbolism, involves correspondences between signal and meaning motivated by cross‐modal or intramodal cognitive biases (see Lockwood & Dingemanse, 2015, for a review). This type of sound symbolism is particularly relevant to this study because it has been shown to facilitate word learning (e.g., Monaghan, Christiansen, & Fitneva, 2011; Nielsen & Rendall, 2012; Nygaard, Cook, & Namy, 2009; Parault & Schwanenflugel, 2006) and is frequently advanced as an explanation for the origin of language. We found significant levels of shape‐based sound symbolism in the emergent languages. There was also some evidence for size‐based sound symbolism in some of the languages using a conservative measure of size.

Compositionality and the arbitrariness of the sign are fundamental principles of language. However, recent research, briefly reviewed above, is suggestive of a more nuanced picture of language structure that our results are aligned with: Sound symbolic structure emerged in all three of our experiments, and, in Experiment 3, we found evidence of sublexical structure that was not compositional in the traditional sense. In the early generations of Experiment 3, the pairs of participants shared little common ground, so they made use of iconic strategies, such as sound symbolism or reduplication. This gave rise to sublexical structure that peaked in each of the chains between Generation 2 and Generation 6. This sublexical structure then gradually started to drop away, perhaps—as Aronoff might argue—because the meanings of the words begin to drift from their compositional origins as “the sign gravitates to the word” (Aronoff, 1976, p. 14). That is to say, the words may be compositional early on and then start to lose this property as they begin to evolve idiosyncratic meanings not predictable from their component parts, just as in natural language where polymorphemic words cannot always be easily decomposed into smaller units of meaning.^8^ We suggest that this aspect of compositionality, as well as a more complete understanding of how iterated learning builds morphemes out of noise—via an interim stage of statistical tendencies—is ripe for future exploration.

## 6 Conclusion

Our meaning space pushes the boundaries on the experimental study of iterated learning by avoiding several simplifications that previous experiments have made. Our meaning space is continuous, unstructured by the experimenter, vast in magnitude, and we do not prompt participants to make a certain number of categorical distinctions. Despite these features of the experimental setup, our first experiment showed that cultural evolution can deliver languages that categorize the meaning space under pressure from learnability. These languages had no string‐internal structure but showed signs of containing sensory sound symbolic patterning. In our second experiment, and unlike previous studies, combining the pressure for learnability with an artificial pressure for expressivity did not lead to signals with internal structure. In our final experiment, we found that combining a pressure for learnability with a pressure for expressivity derived from a genuine communicative task gave rise to languages that use both categorization and string‐internal structure to be both learnable and expressive. Unlike previous work, this emergent structure was sublexical rather than morphosyntactic, and as such bears similarities to certain aspects of natural lexicons, combining both conventional and sensory sound symbolism.

## Supporting information


**Appendix S1.** Experimental briefs

**Appendix S2.** Geometric measure of triangle dissimilarity

**Appendix S3.** MDS plots for all generations in all chains

## Notes

1 Although we do not test these predictions in this paper, we do use the notion of semantic convexity in our analyses. This notion states that “a subset *C* [i.e., a category] of a conceptual space *S* [i.e., a meaning space] is said to be *convex* if, for all points *x* and *y* in *C*, all points between *x* and *y* are also in *C*” (Gärdenfors, 2000, p. 69). In other words, the members of a category form a single region of a meaning space in which it is possible to travel between any two members in a straight line without leaving the region.

2 The number of possible triangles in a finite space is uncountably infinite given the set of real numbers. However, the number of triangle stimuli in our meaning space is limited by the resolution of the display and ultimately by what participants are able to perceive as distinct. The latter is difficult to precisely quantify, but for the purpose of this paper, the space can be assumed to be vast in magnitude.

3 The minimum number of insertions, deletions, and substitutions that must be made to one string to transform it into another. The distance is normalized by dividing by the length of the longer string.

4 Correlation between the original dissimilarity ratings and the corresponding Euclidean distances in MDS space: .83. Stress‐1 value: .25.

5 The denominator in Eq. (2) is the upper bound on the area of a triangle of given perimeter. When the ratio is 1, the triangle has maximum area given its perimeter and is therefore equilateral; as the ratio approaches 0, the triangle becomes increasingly thin and pointed.

6 Square root of the sum of squared distances from the centroid of the triangle to its vertices.

7 Cf. Dingemanse, Blasi, Lupyan, Christiansen, and Monaghan (2015), who refer to these notions under the terms “systematicity” and “iconicity.”

8 For example, the meaning of *reduce* is not predictable from *re*‐ and ‐*duce*, despite the fact that these morphemes appear in other English words: *receive*,* refer*,* repel*;* deduce*,* induce*,* produce* (Aronoff, 1976). However, the Latin etymology of these words indicates that they were indeed compositional in the past: *reducere* = *to lead back*,* referre* = *to carry back*,* repellere* = *to drive back*, etc.

## Appendix A Online dissimilarity rating task

To measure the dissimilarity between pairs of triangles, we conducted an online experiment on the crowdsourcing platform CrowdFlower. A standard rating procedure was adopted, which is considered to be more reliable than other, more economical methods (Giordano et al., 2011). We collected dissimilarity ratings for the 1,128 pairs of triangles in the static set. The pairs of stimuli were randomly divided into 8 subsets of 141 pairs. This was repeated 12 times, resulting in 96 subsets, each to be assigned to an individual participant. We paid a flat rate of $0.50 for each of the 96 participants who completed the task. To access the task, participants had to correctly answer three simple entry questions, which evaluated their ability to understand basic English instructions; anyone who failed to answer these questions correctly was not allowed to enter the task. The participants were told that they would see pairs of triangles and would have to “rate how similar the two triangles are” using a slider control. The main part of the task was preceded by a 1 min familiarization stage in which participants were shown all 48 triangles in the static set to give them a sense of the maximum and minimum dissimilarity.

On each trial, the pair of triangles were presented side by side in 500×500‐pixel dashed, gray bounding boxes. The slider control was located below the triangles and was labeled with *very similar* on one end and *very different* on the other; the direction of the scale was determined randomly for each participant. The slider had 1,001 levels of granularity, where 0 is maximally similar and 1,000 is maximally dissimilar. The participant could not proceed to the next trial until at least 3 s had passed and the slider control had been moved. After giving a rating, the participant had to press the enter key, which removed the triangles and slider from the screen, and then click a button labeled *next*, which was centered at the top of the screen; this forced the participant to move the mouse cursor to the top of the screen where it would be approximately equidistant from all points on the slider on the following trial.

There were six practice trials at the beginning of the experiment and three reliability trials randomly interspersed among the normal trials (for a total of 150 trials). In reliability trials, participants were shown identical triangles and should therefore have rated them with a low dissimilarity rating; this was included to monitor participants’ reliability. Due to a browser compatibility issue, a small portion of ratings (5.7%) were not recorded. After excluding these ratings, an average of 11.32 (*SD*: 1.48) independent ratings were collected for each pair of triangle stimuli. The median dissimilarity rating (on the 1,000‐point scale) for reliability trials was 0, suggesting that participants were attending to the stimuli. Two participants were excluded because their mean ratings of reliability pairs were > 100.

The remaining 94 participants’ ratings were normalized in [0, 1] such that the ratings would use the entire width of the scale. The normalized ratings were then averaged together to produce a mean dissimilarity rating for each pair of triangles. Individual rater agreement was measured by correlating an individual participant's ratings with the corresponding mean dissimilarity ratings for the 94 participants as a whole. Mean rater agreement was .7 (range: .22–.88). The three participants whose rater agreement was < .4 were then excluded, leaving a total of 91 participants.

The final distance matrix used in the main analysis was produced by averaging together the normalized ratings for the final 91 participants. There was an average of 10.72 (*SD*: 1.55) independent ratings per pair. Interrater reliability among the 91 participants was measured using Krippendorff's alpha coefficient (Krippendorff, 1970), which is applicable where multiple raters each rate incomplete but overlapping subsets of the full data set. The value of this statistic was .41, which is quite low; however, this should not be surprising given that participants were not instructed on specifically how to judge the dissimilarity between triangles, so some diversity in ratings was to be expected.

## Appendix B Dissimilarity judgments between target and selected triangles in Experiment 3

Unless otherwise noted, this online experiment was identical to that described inAppendix A above. The 80 participants who took part in Experiment 3 selected the wrong triangle from the context array a total of 2,653 times. For a more granular measure of communicative error, we wanted to quantify the dissimilarity between the target and selected triangles in each of these cases. The 2,653 pairs were randomly divided into 21 subsets (14 subsets of 126 pairs and 7 subsets of 127 pairs). This was repeated 10 times, resulting in 210 subsets to be assigned to individual participants. We paid a flat rate of $0.45 for each of the 184 participants who completed the task. There were six practice trials at the beginning and three reliability trials randomly interspersed among the normal trials (for a total of 135 or 136 trials).

The median number of independent ratings collected for each pair was 9 (range: 4–10). The median dissimilarity rating for reliability trials was 0. One participant was excluded because they rated all triangle pairs as having maximum dissimilarity. An additional 32 participants were excluded because their mean ratings of reliability pairs were > 100. The remaining 151 participants’ ratings were normalized and averaged together to produce a mean dissimilarity rating for each pair of triangles. Mean rater agreement was .69 (range: .36–.87). The three participants whose rater agreement was  < .4 were then excluded, leaving a total of 148 participants. The final dissimilarity ratings used in the main analysis were produced by averaging together the normalized ratings given by the final 148 participants. The mean number of independent ratings per pair of triangles was 7.04 (*SD*: 1.4). Krippendorff's alpha for interrater reliability was .37.
