# Supplementary material for: The Cultural Evolution of Structured Languages in an Open‐Ended, Continuous World
Source: Cogn Sci. 2016 Apr 7;41(4):892–923. doi: 10.1111/cogs.12371 (PMC5484388; doi:10.1111/cogs.12371)
Supplement: Supplementary file 1 — Appendix S1. Experimental briefs [file COGS-41-892-s001.pdf]

# Flatlanders experiment

## Brief

You have just entered a parallel universe that has only two dimensions! This curious place is inhabited by an intelligent life form, the Flatlanders, who are obsessed with two-dimensional shapes and have a huge vocabulary just for triangles alone.

Your task is to learn the words that the Flatlanders use for triangles to help us establish contact with these strange beings. It's a pretty difficult task — but we think you're the right person for the job!

**Stage 1: Training**

You will see a series of triangles, one by one. Each triangle will be presented with its name in the Flatlander language. The name will also be pronounced by the computer to help you learn it. After every third triangle, you will see one of those three triangles again and you must type in its name. This stage is designed to help you learn the language.

**Stage 2: Test**

Again, you will see a series of triangles. For each triangle, simply type in what you think it's called based on the training you completed in stage 1. The test is designed to assess how well you've learned the Flatlander language, and there's a £20 Amazon voucher for whoever learns it best.

You will learn a lot of words very quickly, and it may be difficult to take it all in. But don't panic! The most important thing is to maintain good relations with the Flatlanders by giving it your best shot. You must type in an answer for every triangle, but it's okay to guess if you're unsure. Even if you get the word wrong, you'll still get points for getting the word partially correct.

Good luck!

# Flatlanders experiment

## Brief

You have just entered a parallel universe that has only two dimensions! This curious place is inhabited by an intelligent life form, the Flatlanders, who are obsessed with two-dimensional shapes and have a huge vocabulary just for triangles alone.

Your task is to learn the words that the Flatlanders use for triangles to help us establish contact with these strange beings. It's a pretty difficult task — but we think you're the right person for the job!

**Stage 1: Training**

You will see a series of triangles, one by one. Each triangle will be presented with its name in the Flatlander language. The name will also be pronounced by the computer to help you learn it. After every third triangle, you will see one of those three triangles again and you must type in its name. This stage is designed to help you learn the language.

**Stage 2: Test**

Again, you will see a series of triangles. For each triangle, simply type in what you think it's called based on the training you completed in stage 1. However, if you use the same word too frequently, you will see a message asking you to use a different word. The test is designed to assess how well you've learned the Flatlander language, and there's a £20 Amazon voucher for whoever learns it best.

You will learn a lot of words very quickly, and it may be difficult to take it all in. But don't panic! The most important thing is to maintain good relations with the Flatlanders by giving it your best shot. You must type in an answer for every triangle, but it's okay to guess if you're unsure. Even if you get the word wrong, you'll still get points for getting the word partially correct.

Good luck!

# Flatlanders experiment

# Brief

You have just entered a parallel universe that has only two dimensions! This curious place is inhabited by an intelligent life form, the Flatlanders, who are obsessed with two-dimensional shapes and have a huge vocabulary just for triangles.

Your task is to learn the words that the Flatlanders use for triangles to help us establish contact with these strange beings. It's a pretty difficult task — but we think you're the right person for the job!

**Stage 1: Training**

You will see a series of triangles, one by one. Each triangle will be presented with its name in the Flatlander language. The name will also be pronounced by the computer to help you learn it. After every third triangle, you will see one of the previous three triangles again and you must type in its name. This stage is designed to help you learn the language.

**Stage 2: Communication**

You and your partner will communicate using the language you learned in Stage 1. When it's your turn to communicate, you'll be presented with a triangle. You will type in the word for this triangle and send it to your partner. Your partner will then see a selection of six triangles and they'll have to figure out which triangle you're talking about. You and your partner will take turns at being the communicator and matcher.

***Important:*** you must only communicate using the Flatlander language that you and your partner learned in Stage 1. You must not use English or any other language to communicate with your partner. The supervisor will be monitoring your communications during the experiment.

You will learn a lot of words very quickly during the training stage, and it may be difficult to take it all in. But don't panic! The most important thing is to maintain good relations with the Flatlanders by giving it your best shot. It's okay to guess if you're unsure. Go with your instinct and type in a word that feels right. Your partner may still be able to identify it, even if it's only partially correct.

The pair who communicate most successfully using the Flatlander language will each receive a £20 Amazon voucher.

Good luck!
