# Supplementary material for: The Cultural Evolution of Structured Languages in an Open‐Ended, Continuous World
Source: Cogn Sci. 2016 Apr 7;41(4):892–923. doi: 10.1111/cogs.12371 (PMC5484388; doi:10.1111/cogs.12371)
Supplement: Supplementary file 2 — Appendix S2. Geometric measure of triangle dissimilarity [file COGS-41-892-s002.pdf]

The Cultural Evolution of Structured Languages in an Open-Ended, Continuous World

Geometric measure of triangle dissimilarity

Jon W. Carr, Kenny Smith, Hannah Cornish, Simon Kirby

The dissimilarity ratings provided by the naïve raters reflect the cognitive perceptions that humans have about triangles. However, the participants in the main experiments may have held different perceptual representations of the triangles from those held by the naïve raters: The participants in our main experiments would have had a notion of triangle dissimilarity in the context of using a language to describe or communicate about the triangles. Here we provide an alternative geometric measure of triangle dissimilarity that explicitly considers a range of possible meaning dimensions, some of which may not have been considered important by the naïve raters. This measure also allows us to isolate particular geometric properties to determine which features were being encoded by the participants in the main experiments.

1 Geometric dissimilarity measure

We selected four features that participants could potentially use to conceptualize and communicate about the triangles (see Table 1). For each of the features, the distance was computed between every pair of triangles in the static set, yielding four distance matrices. The matrices were converted to ranks to remove the distributional effects peculiar to each metric, summed together, and then normalized in the interval [0, 1]. A pair of triangles that are similar in terms of all four features will have a score close to 0 (with 0 representing identity), while a pair of triangles that are dissimilar in terms of all four features will have a score close to 1. Fig. 1 shows the most similar and most dissimilar pairs of triangle stimuli based on this geometric measure (top) and based on the ratings from the naïve raters (bottom) for comparison.

There was a strong correlation between the scores produced from this geometric approach and the mean normalized dissimilarity ratings provided by the naïve raters ($r = .49$, $n = 1128$, $p < .001$; Mantel test). The results for structure using this measure are given in Fig. 2. The general trends are congruent with the equivalent results produced using the ratings from naïve raters. However, the structure scores tend to be lower under the geometric approach, suggesting that it does not fully capture the way in which the triangles are perceived. For this reason, we consider the structure results based on human ratings to be canonical and present this alternative method in support of our conclusions.

Table 1: Set of four features and the corresponding distance metrics used to measure the dissimilarity between a pair of triangles *X* and *Y*

**Feature**	**Metric**	**Pseudocode**
Location	Euclidean distance between centroids	`euclidDist(cent(X),cent(Y))`
Orientation	Shortest angular distance by orienting spot[a]	`min(angCW(X,Y),angCCW(X,Y))`
Shape	Absolute difference in equilateralness ratio[b]	`abs(equilat(X)-equilat(Y))`
Size	Absolute difference in centroid size[c]	`abs(centSize(X)-centSize(Y))`

[a]Orientation is defined as the angular coordinate of the orienting spot when the triangle is centered on the origin. The shortest angular distance between two triangles is the shorter of the clockwise or counterclockwise angular distances. [b]See Equation 2 in the main paper. [c]Square root of the sum of squared distances from the centroid of the triangle to its vertices.

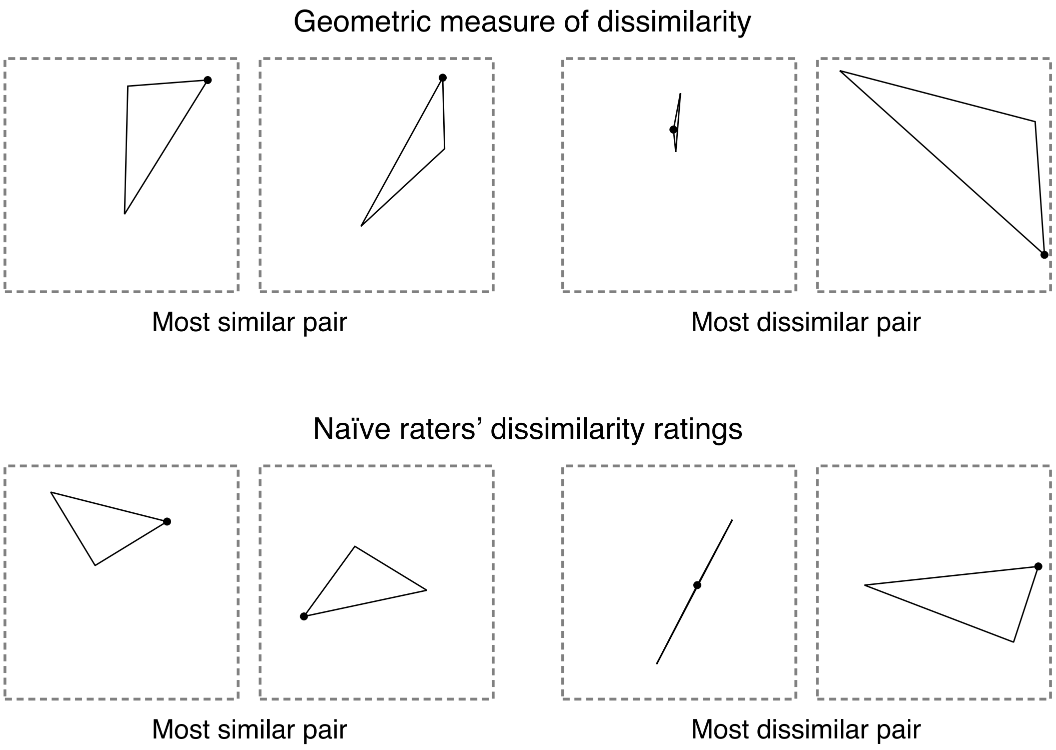

Figure 1: The most similar and most dissimilar pairs of triangles in the static set based on the geometric measure of dissimilarity (top) and the ratings of the naïve raters (bottom). Note that, under the geometric measure, all four features are taken into account, while the human raters appear to be ignoring the properties of location and orientation.

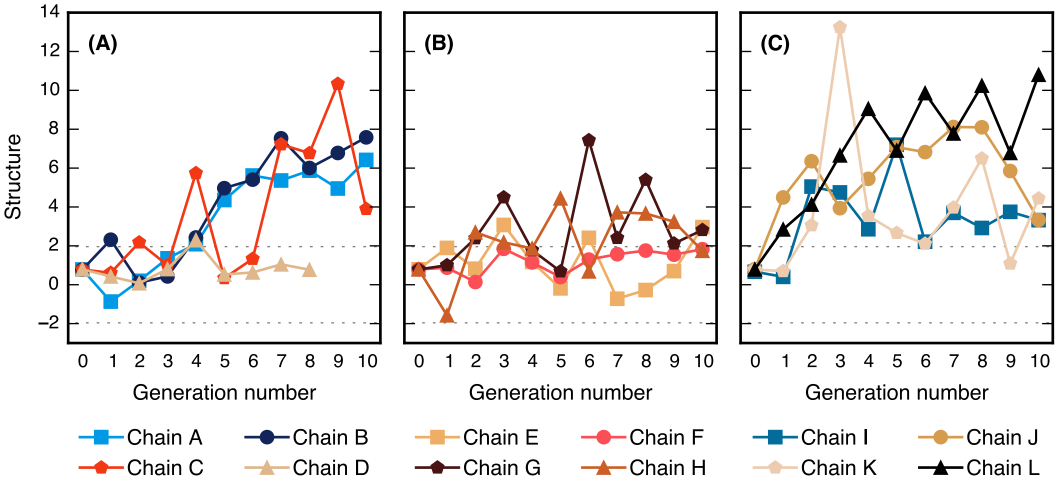

Figure 2: Levels of structure in (A) Experiment 1, (B) Experiment 2, and (C) Experiment 3 using the geometric measure of triangle dissimilarity rather than human dissimilarity ratings. The results are congruent with those presented in the main paper, although the structure scores are generally lower.

2 Encoded meaning dimensions

To determine the features encoded by a particular language, we correlated the pairwise string dissimilarity scores with all combinations of the four geometric features described in Table 1 to see which combination would yield the strongest correlation. For four features, there are $\sum_{i=1}^{4} \binom{4}{i} = 15$ combinations to consider, giving us a typology of 15 types of language that could potentially arise. These language types are listed in Table 2 along with reference numbers; for example, a Type 13 language encodes location, shape, and size.

The results of this analysis are given in Table 3; for all generations, the table gives the type number for the combination of features that resulted in the strongest correlation, along with the Pearson correlation in parentheses. The most common types of language to emerge across all experiments were Type 3 (shape; 52% of languages) and Type 10 (shape and size; 17% of languages). The language types with the highest average correlation (across all emergent languages) were Type 3 (shape; mean $r = .19$) and Type 10 (shape and size; mean $r = .16$). These results reveal a clear bias toward encoding the shape and size features of the triangles.

This analysis was also performed with the dissimilarity ratings from the naïve raters; the strongest correlations were also with Type 3 (shape; $r = .71$, $n = 1128$, $p < .001$; Mantel test) and Type 10 (shape and size; $r = .69$, $n = 1128$, $p < .001$; Mantel test), suggesting that the naïve raters were also rating the dissimilarity between triangles based primarily on the shape and size features. This is supported by the fact that the dimensions of the MDS solution corresponded approximately to shape and size.

Table 2: List of language types

Type #	Encoded meaning dimensions
1	Location
2	Orientation
3	Shape
4	Size
5	Location, Orientation
6	Location, Shape
7	Location, Size
8	Orientation, Shape
9	Orientation, Size
10	Shape, Size
11	Location, Orientation, Shape
12	Location, Orientation, Size
13	Location, Shape, Size
14	Orientation, Shape, Size
15	Location, Orientation, Shape, Size

Table 3: Encoded meaning dimensions for each emergent language

	1	2	3	4	5	6	7	8	9	10
					**Experiment 1**					
**A**	1 (.06)	3 (.05)	11 (.05)	14 (.07)	3 (.2)	3 (.31)	3 (.45)	10 (.42)	10 (.3)	3 (.46)
**B**	15 (.08)	3 (.05)	3 (.12)	3 (.17)	3 (.4)	3 (.44)	3 (.44)	3 (.52)	3 (.45)	3 (.51)
**C**	5 (.07)	10 (.12)	3 (.07)	9 (.26)	3 (.11)	3 (.06)	3 (.37)	3 (.29)	3 (.47)	3 (.37)
**D**	14 (.05)	3 (.07)	3 (.09)	13 (.17)	6 (.04)	1 (.06)	–	1 (.16)	–	–
					**Experiment 2**					
**E**	11 (.08)	13 (.06)	14 (.11)	10 (.05)	4 (.06)	6 (.11)	3 (.07)	10 (.03)	4 (.06)	7 (.19)
**F**	9 (.08)	4 (.04)	7 (.09)	7 (.08)	7 (.03)	10 (.06)	12 (.06)	10 (.08)	3 (.14)	3 (.12)
**G**	10 (.11)	6 (.12)	3 (.23)	8 (.06)	10 (.04)	10 (.31)	10 (.17)	3 (.36)	6 (.1)	3 (.16)
**H**	4 (0)	11 (.14)	6 (.1)	10 (.08)	10 (.2)	3 (.14)	3 (.21)	3 (.21)	3 (.14)	6 (.1)
					**Experiment 3**					
**I**	10 (.08)	11 (.17)	10 (.24)	3 (.18)	3 (.3)	3 (.15)	3 (.33)	3 (.24)	3 (.28)	3 (.25)
**J**	6 (.18)	3 (.33)	3 (.22)	3 (.3)	3 (.36)	3 (.38)	3 (.51)	3 (.37)	3 (.36)	10 (.15)
**K**	2 (.22)	3 (.16)	3 (.45)	13 (.14)	3 (.17)	3 (.17)	10 (.2)	10 (.24)	3 (.14)	10 (.3)
**L**	1 (.14)	3 (.24)	3 (.42)	3 (.49)	3 (.52)	3 (.57)	3 (.41)	3 (.61)	3 (.36)	10 (.48)

Note. Each cell gives the type number (see Table 2) for the combination of features that resulted in the strongest correlation; the correlation coefficient is given in parentheses.
