# Supplementary material for: The Cultural Evolution of Structured Languages in an Open‐Ended, Continuous World
Source: Cogn Sci. 2016 Apr 7;41(4):892–923. doi: 10.1111/cogs.12371 (PMC5484388; doi:10.1111/cogs.12371)
Supplement: Supplementary file 3 — Appendix S3. MDS plots for all generations in all chains [file COGS-41-892-s003.pdf]

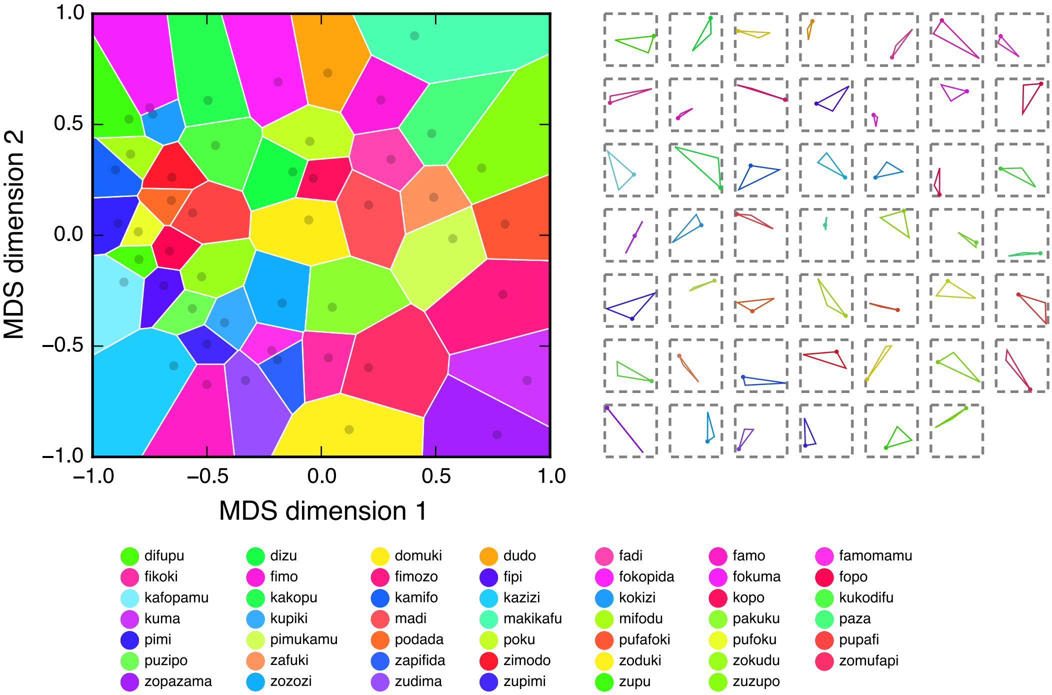

Experiment 1 Chain A Generation 0

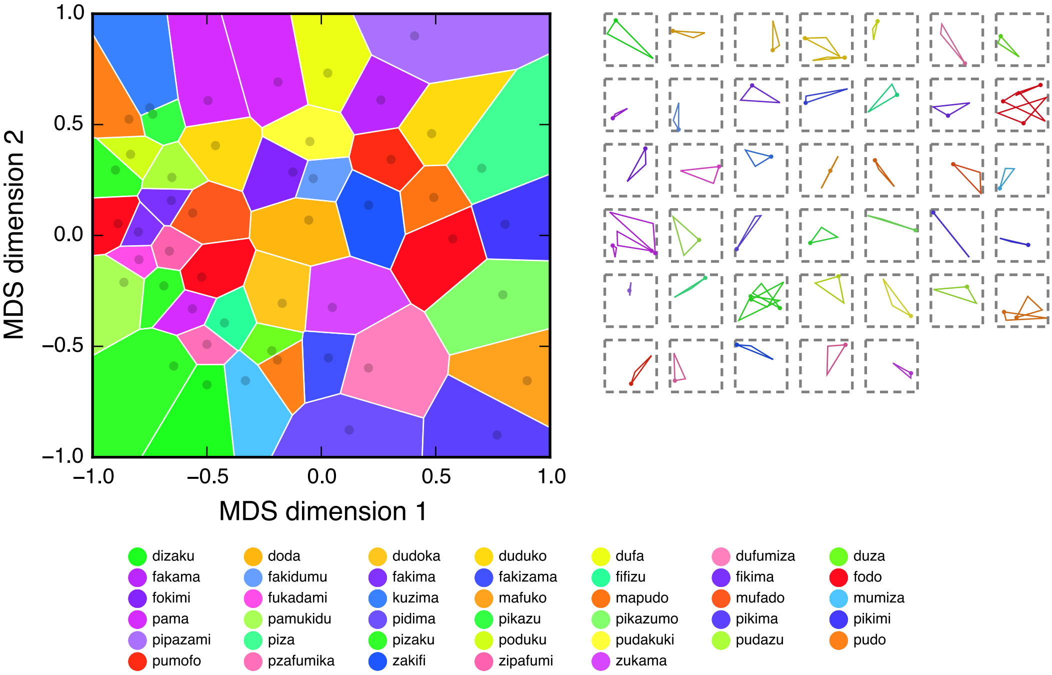

Experiment 1 Chain A Generation 1

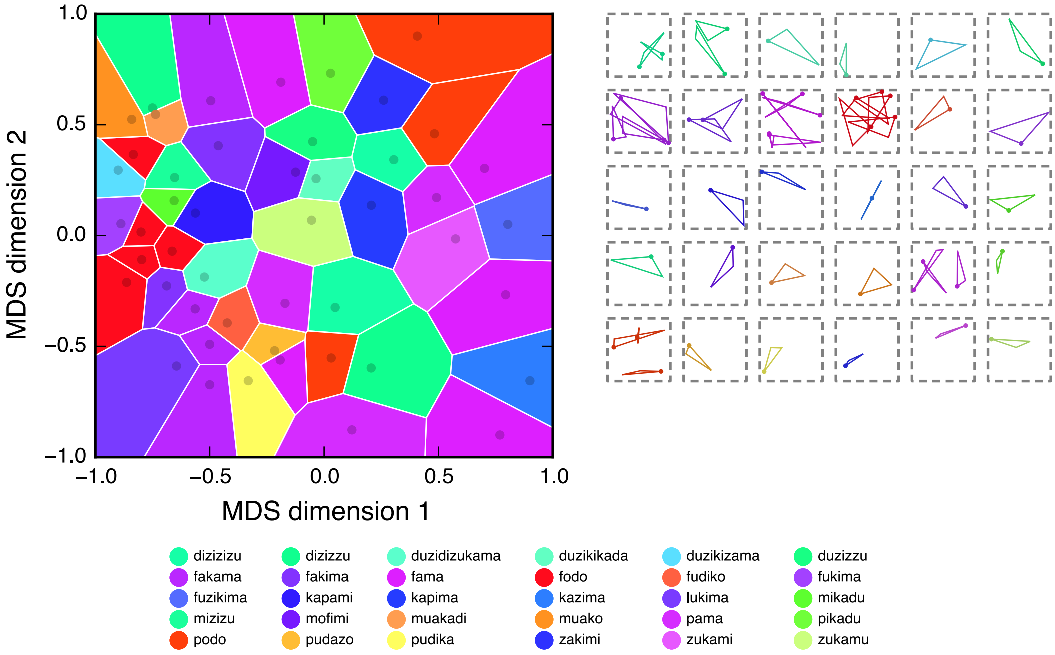

Experiment 1 Chain A Generation 2

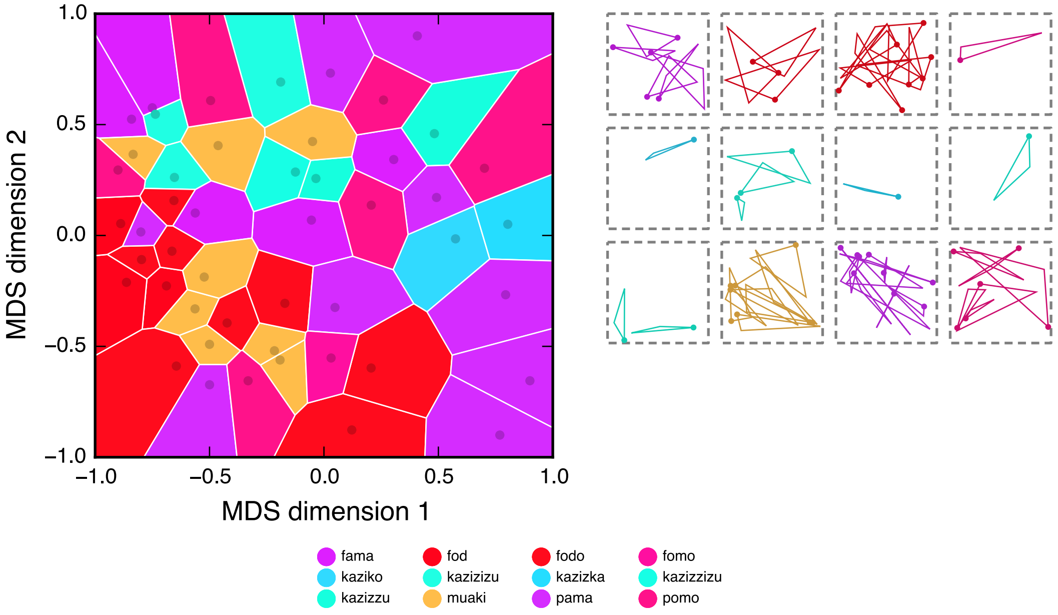

Experiment 1 Chain A Generation 3

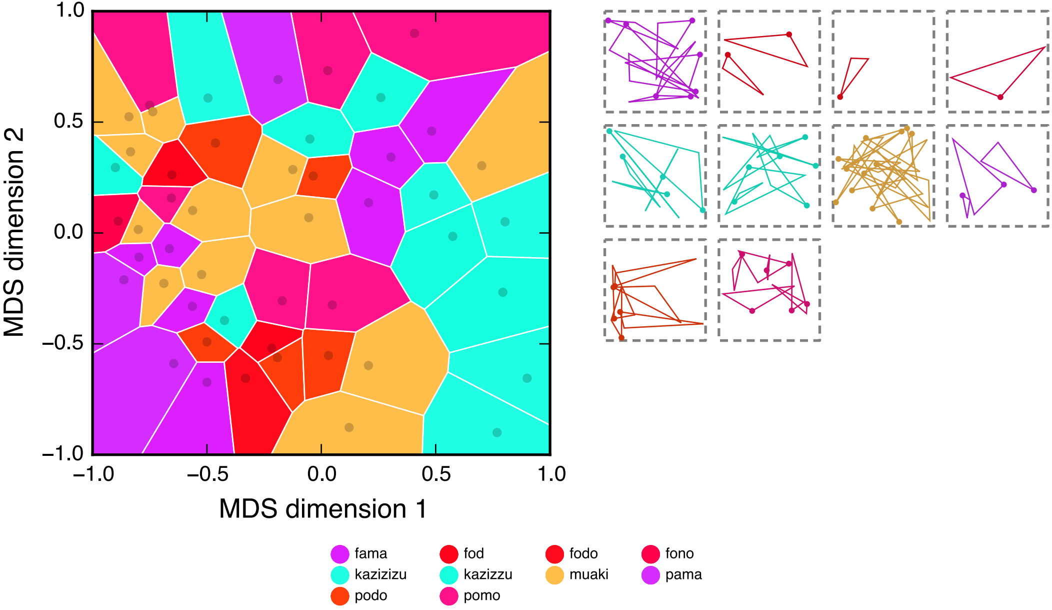

Experiment 1 Chain A Generation 4

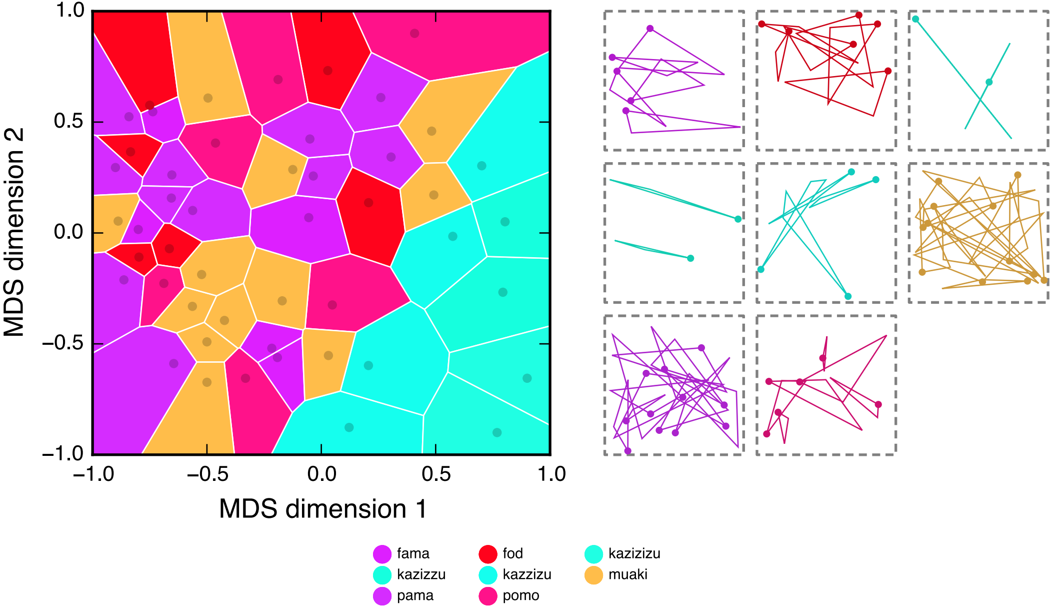

Experiment 1 Chain A Generation 5

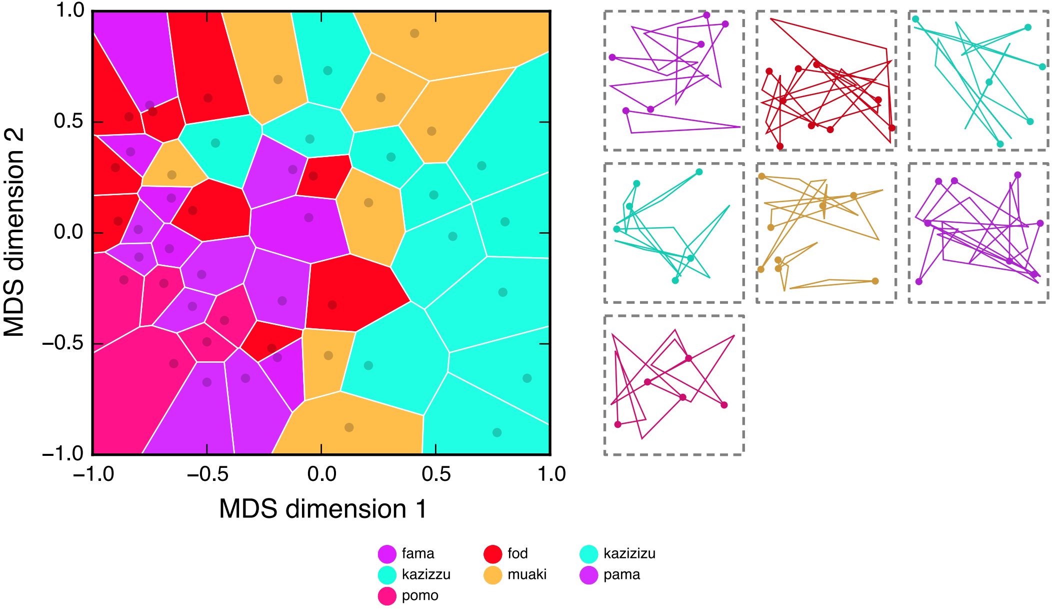

Experiment 1 Chain A Generation 6

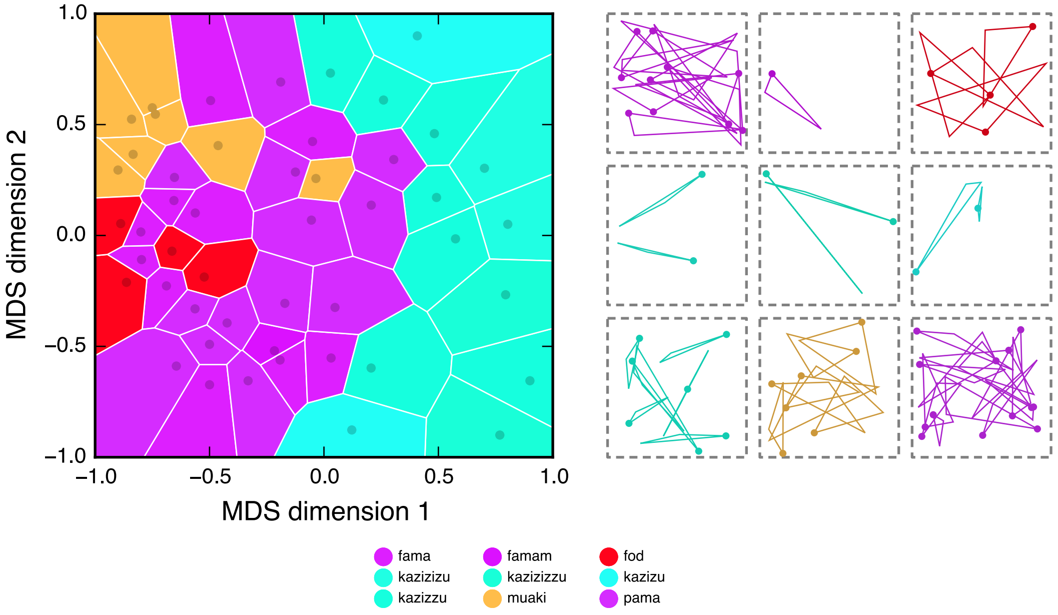

Experiment 1 Chain A Generation 7

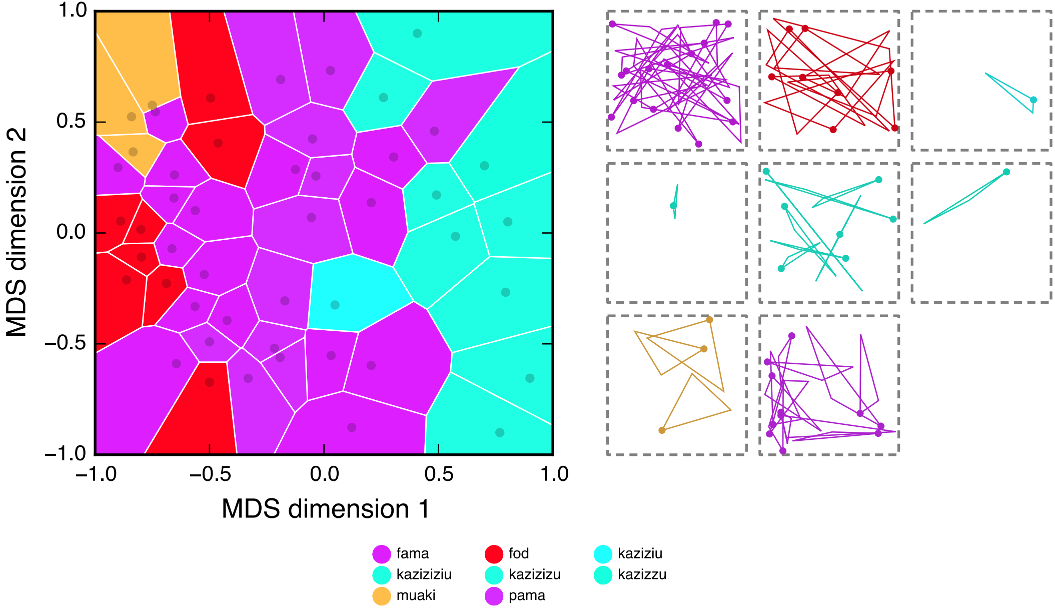

Experiment 1 Chain A Generation 8

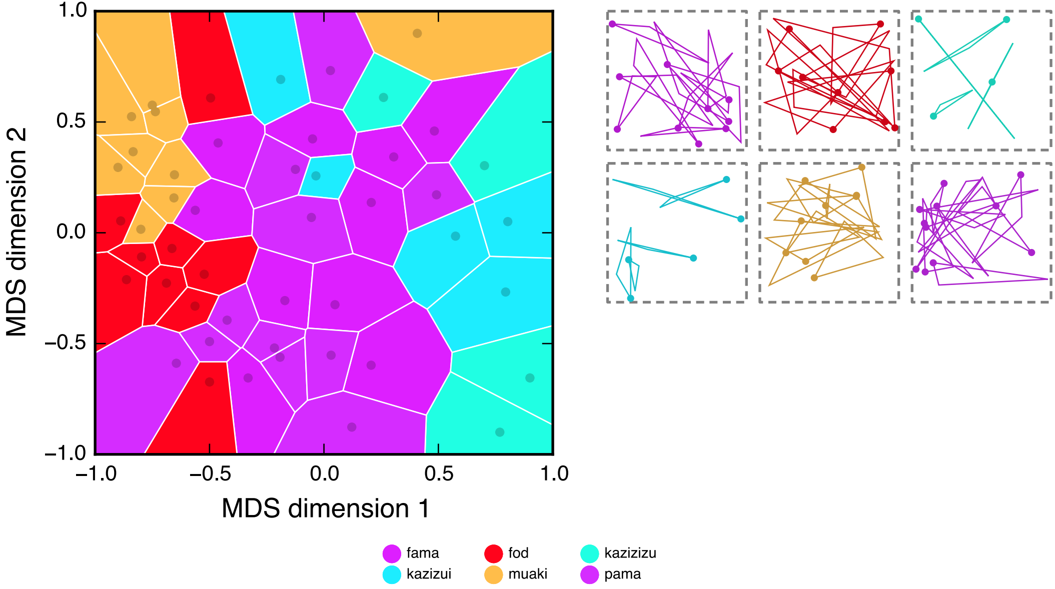

Experiment 1 Chain A Generation 9

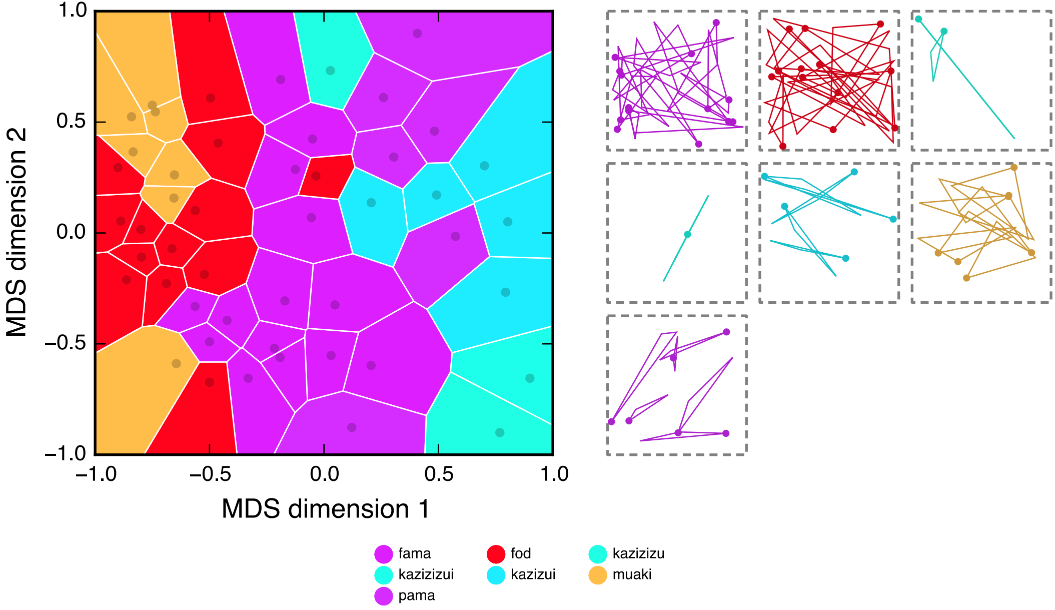

Experiment 1 Chain A Generation 10

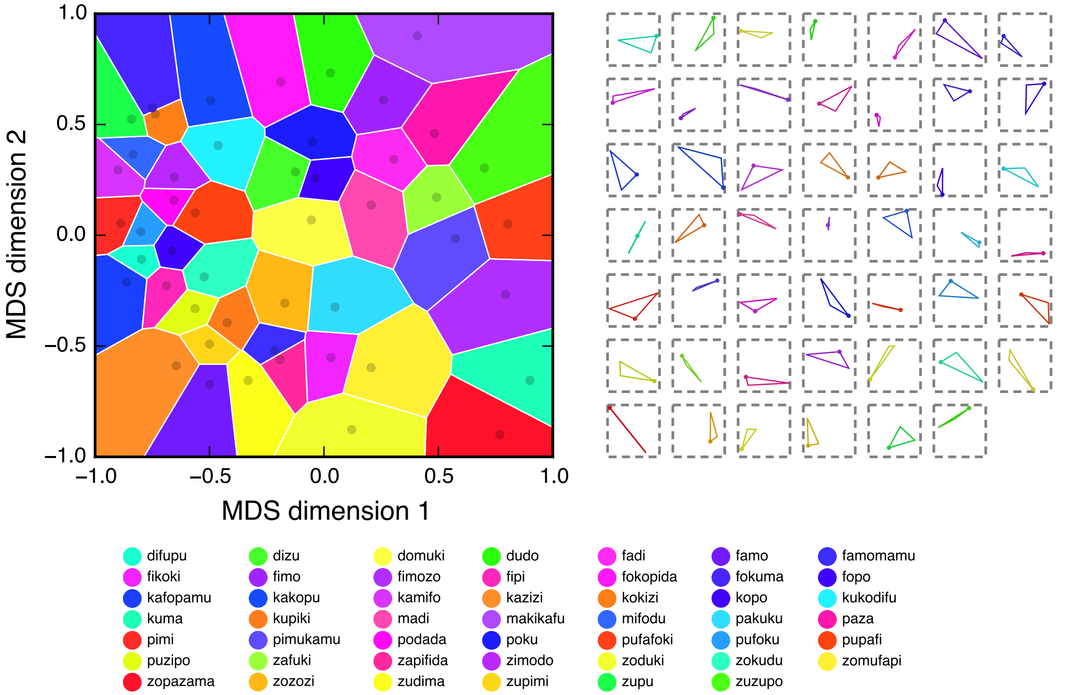

Experiment 1 Chain B Generation 0

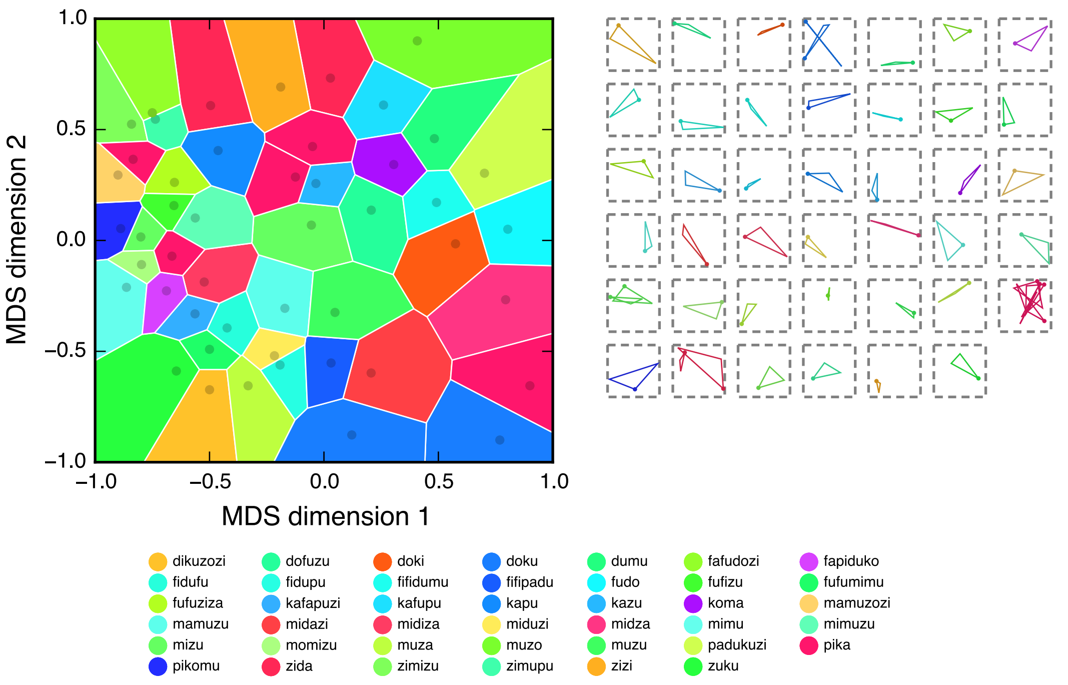

Experiment 1 Chain B Generation 1

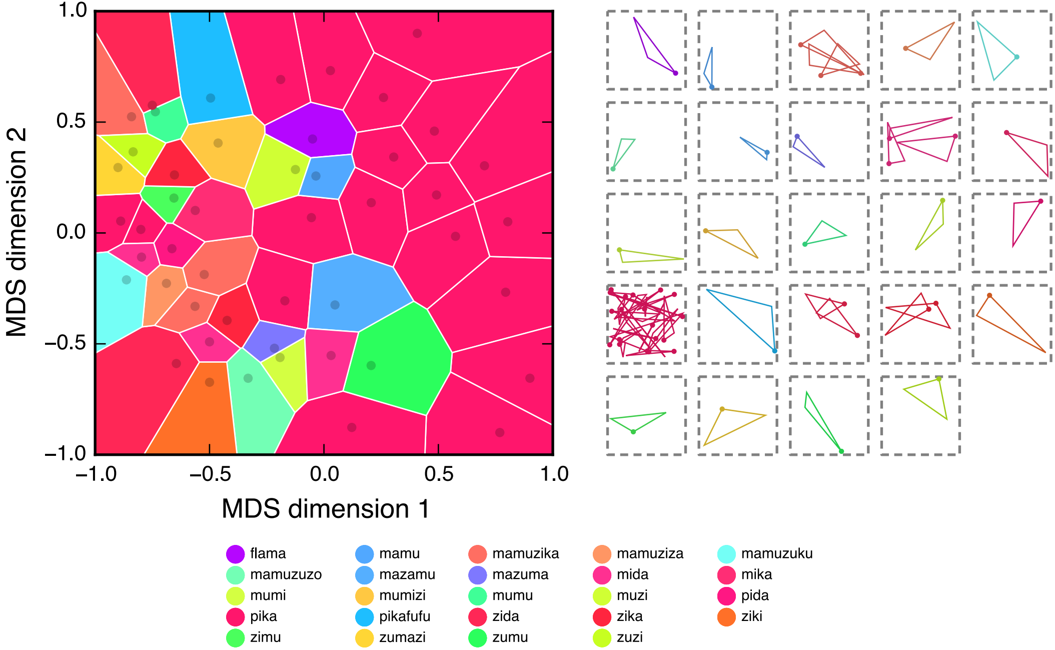

Experiment 1 Chain B Generation 2

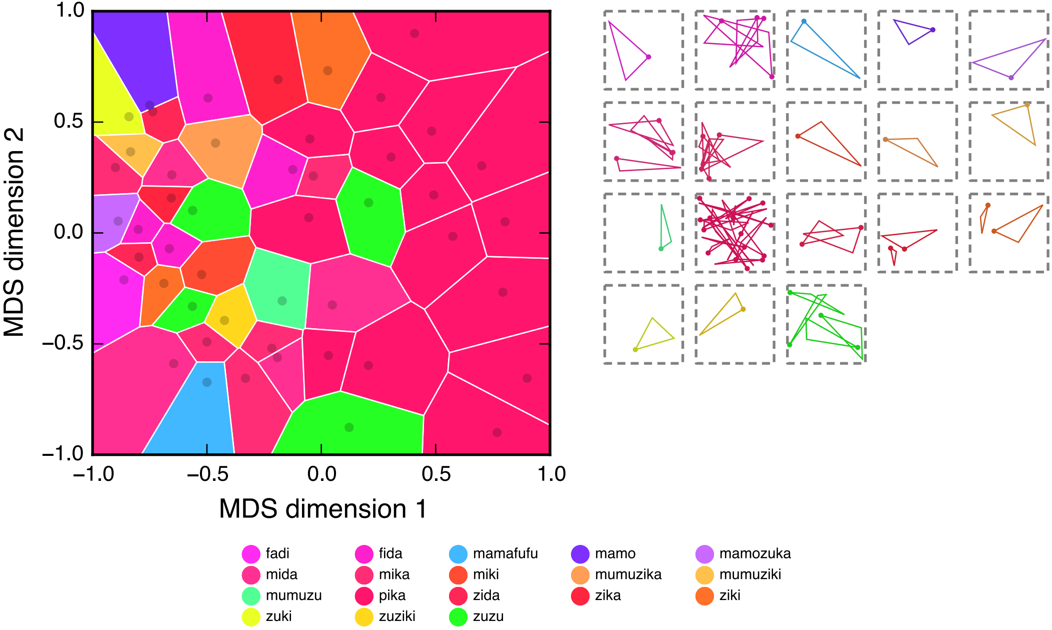

Experiment 1 Chain B Generation 3

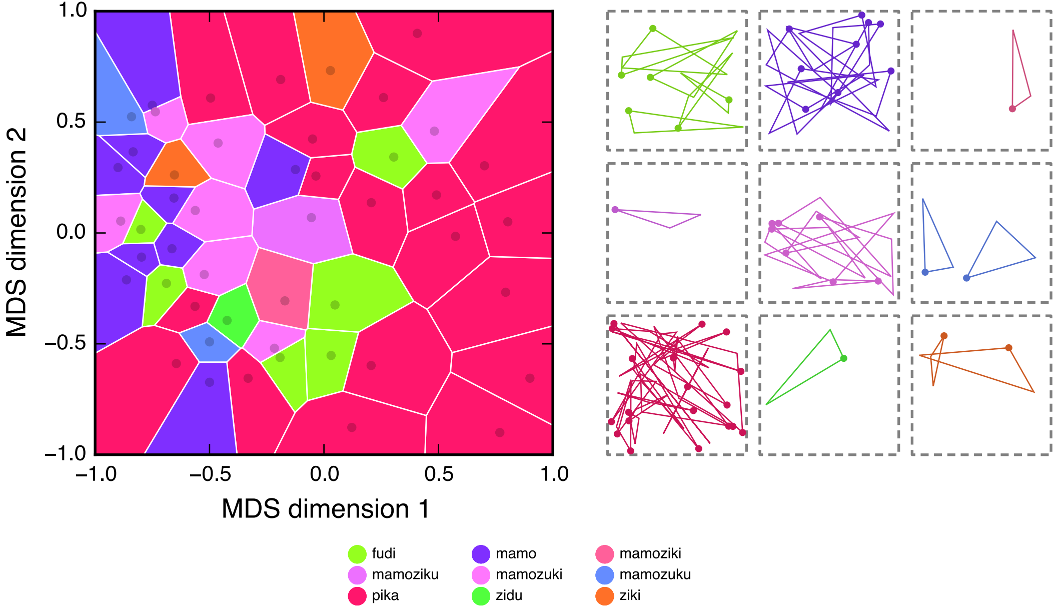

Experiment 1 Chain B Generation 4

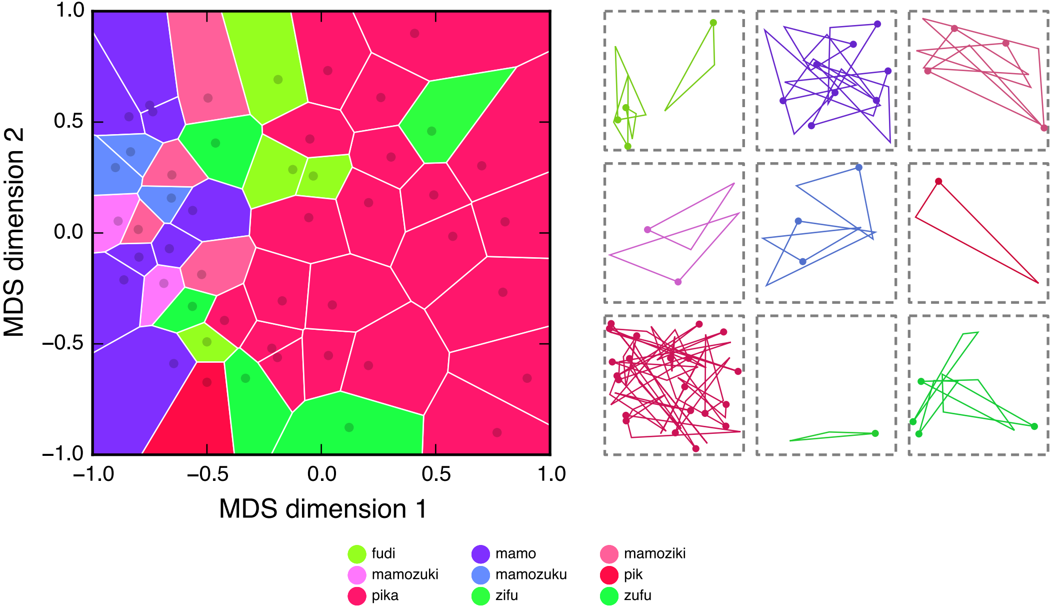

Experiment 1 Chain B Generation 5

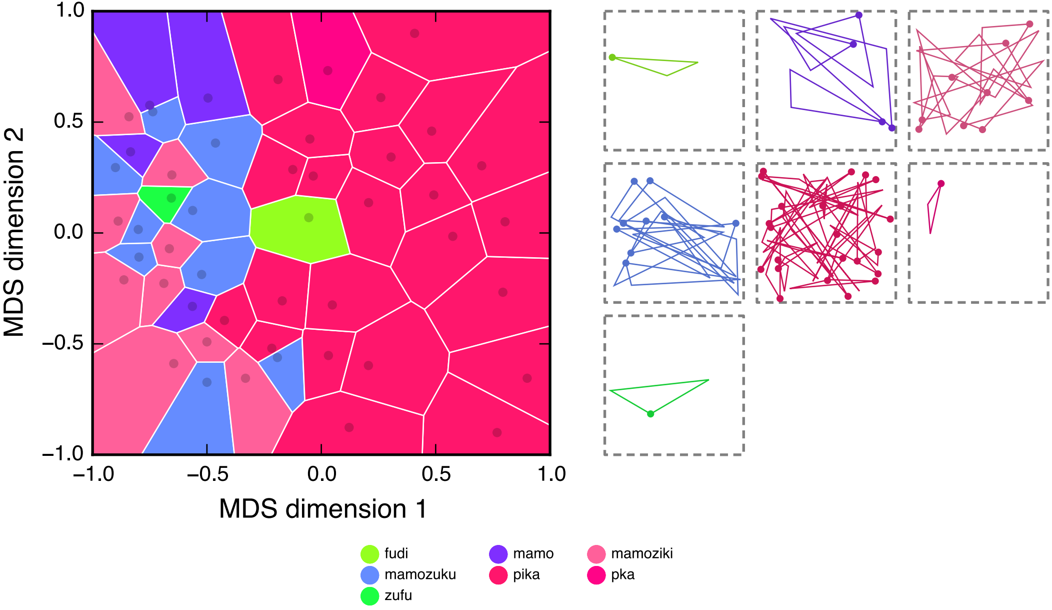

Experiment 1 Chain B Generation 6

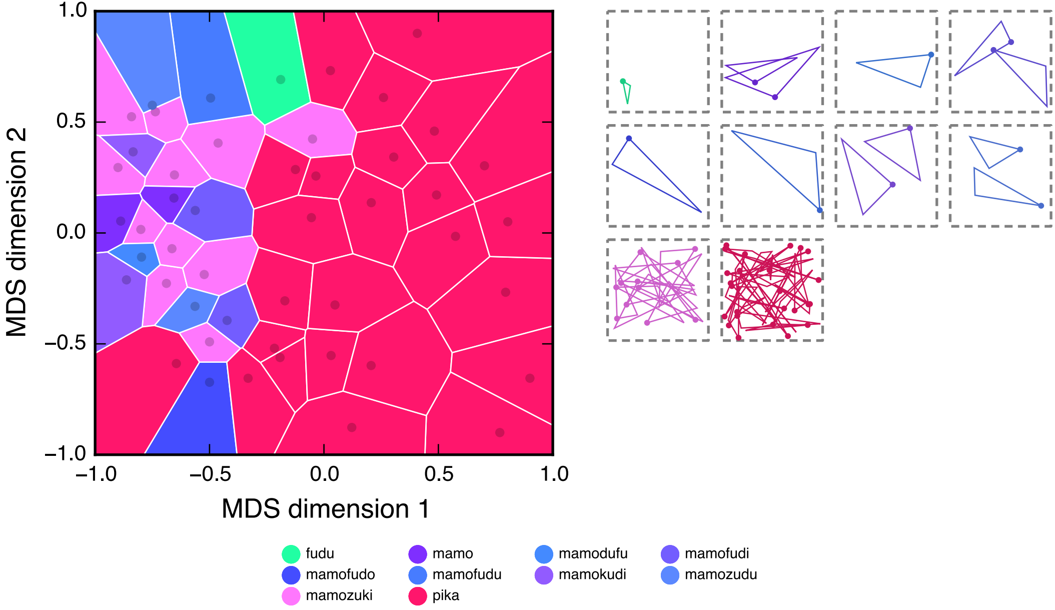

Experiment 1 Chain B Generation 7

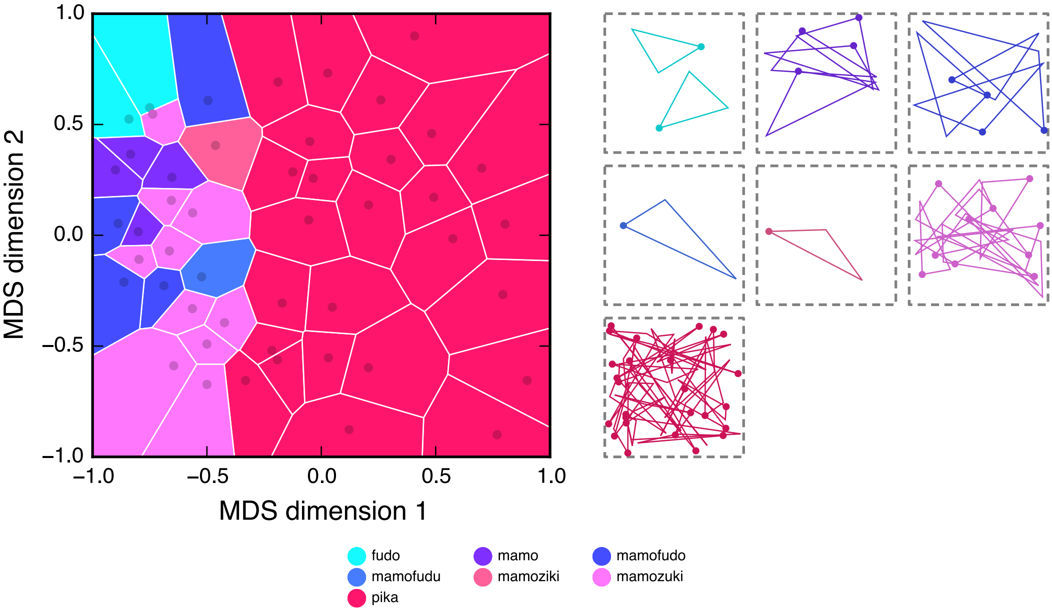

Experiment 1 Chain B Generation 8

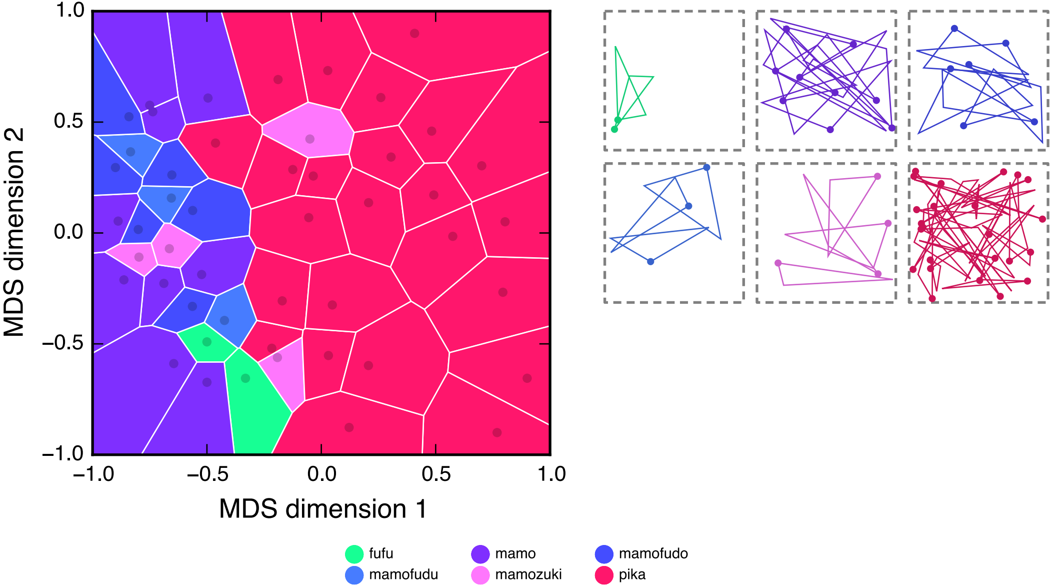

Experiment 1 Chain B Generation 9

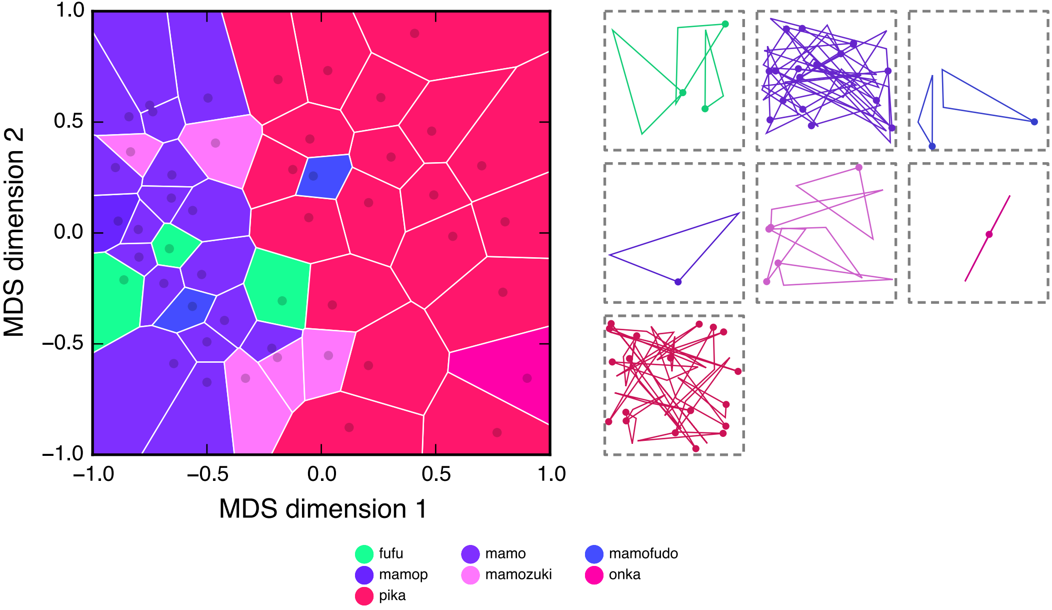

Experiment 1 Chain B Generation 10

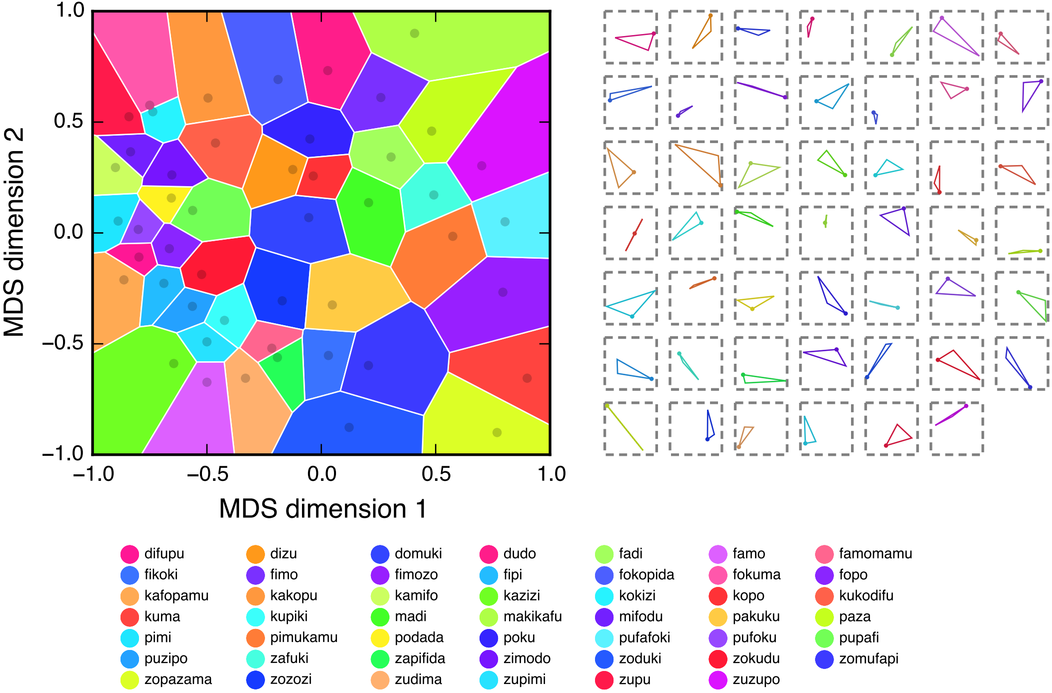

Experiment 1 Chain C Generation 0

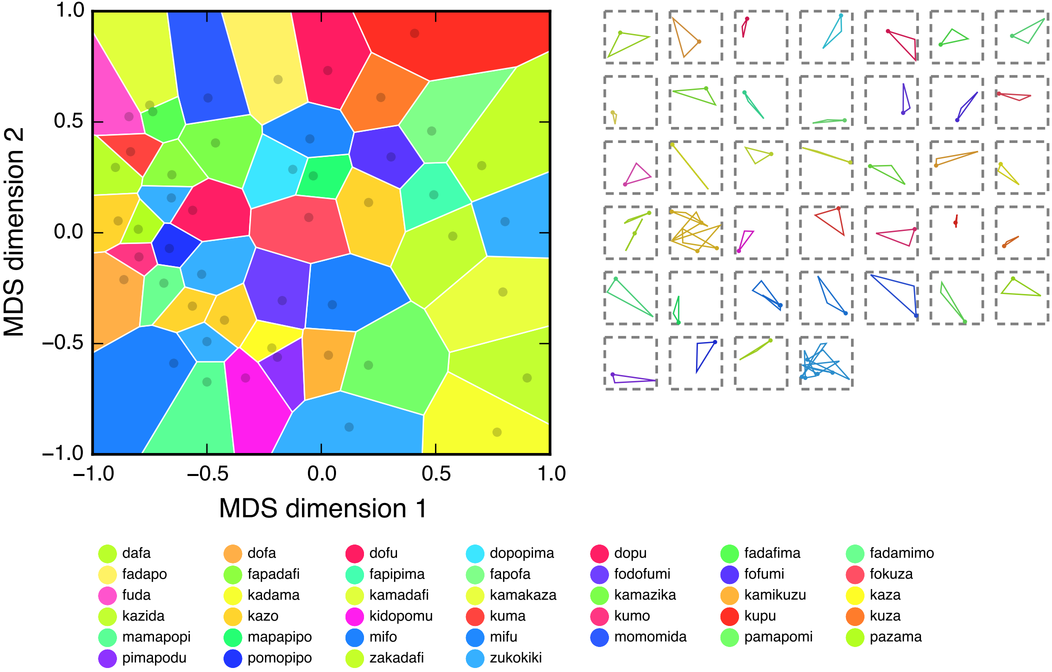

Experiment 1 Chain C Generation 1

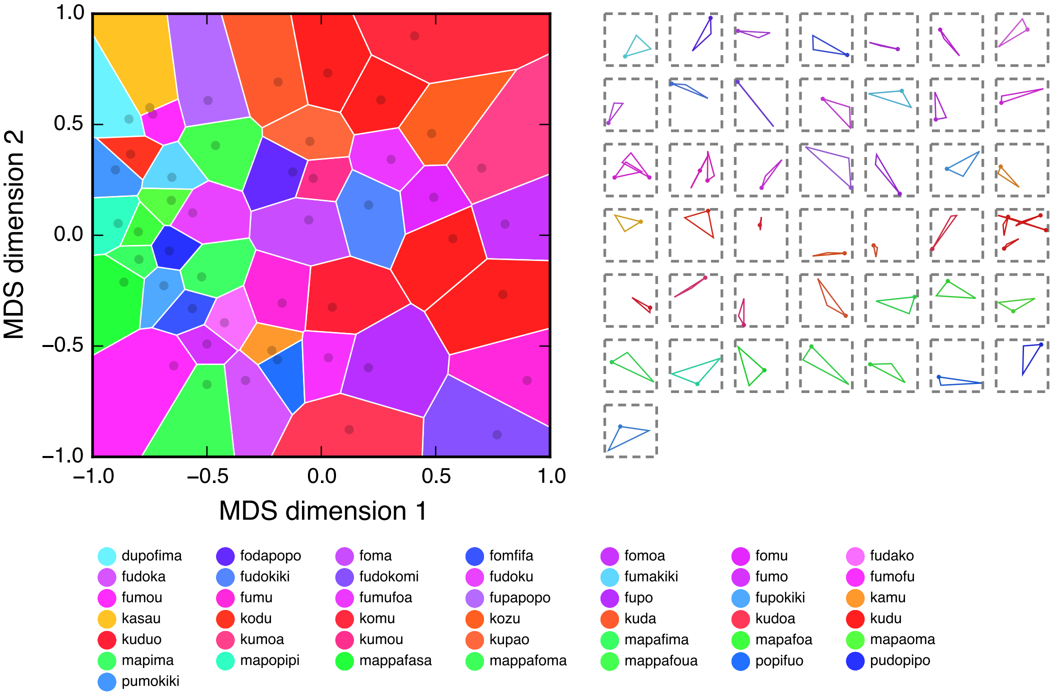

Experiment 1 Chain C Generation 2

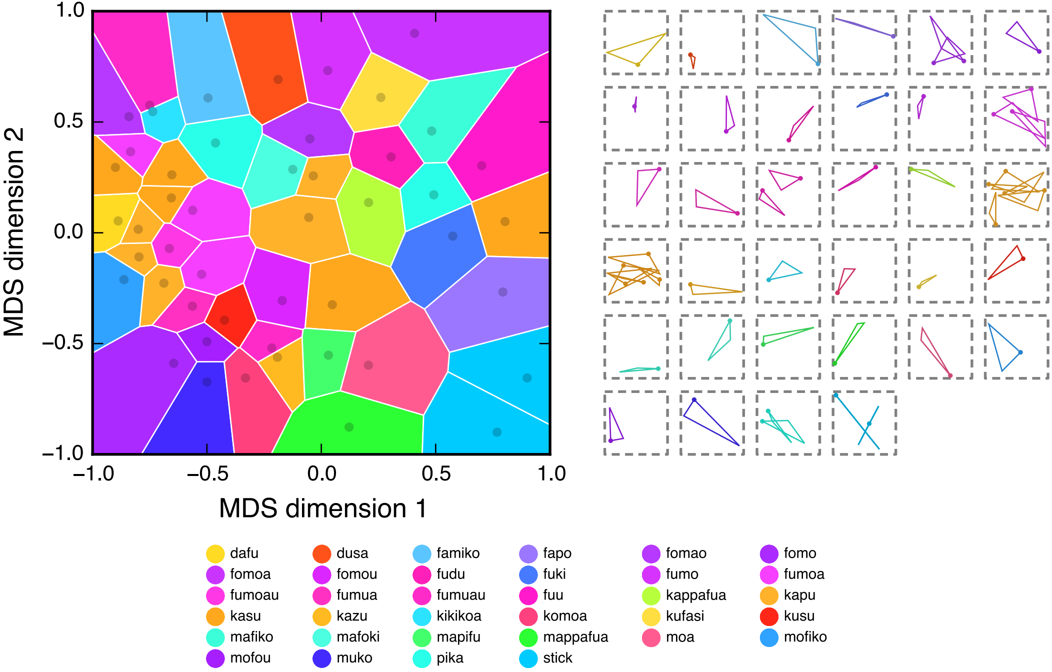

Experiment 1 Chain C Generation 3

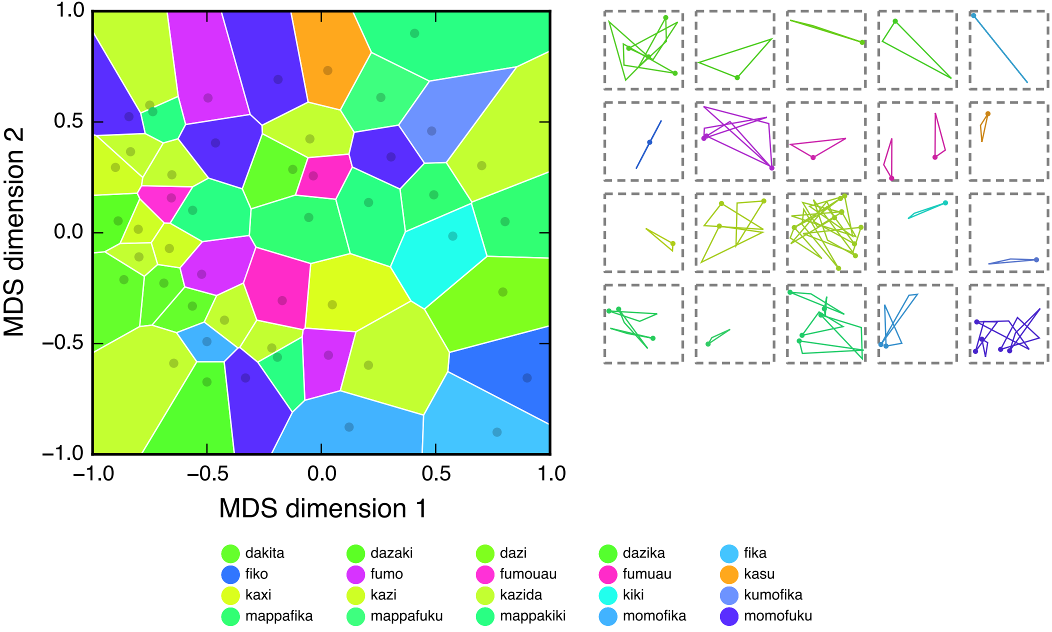

Experiment 1 Chain C Generation 4

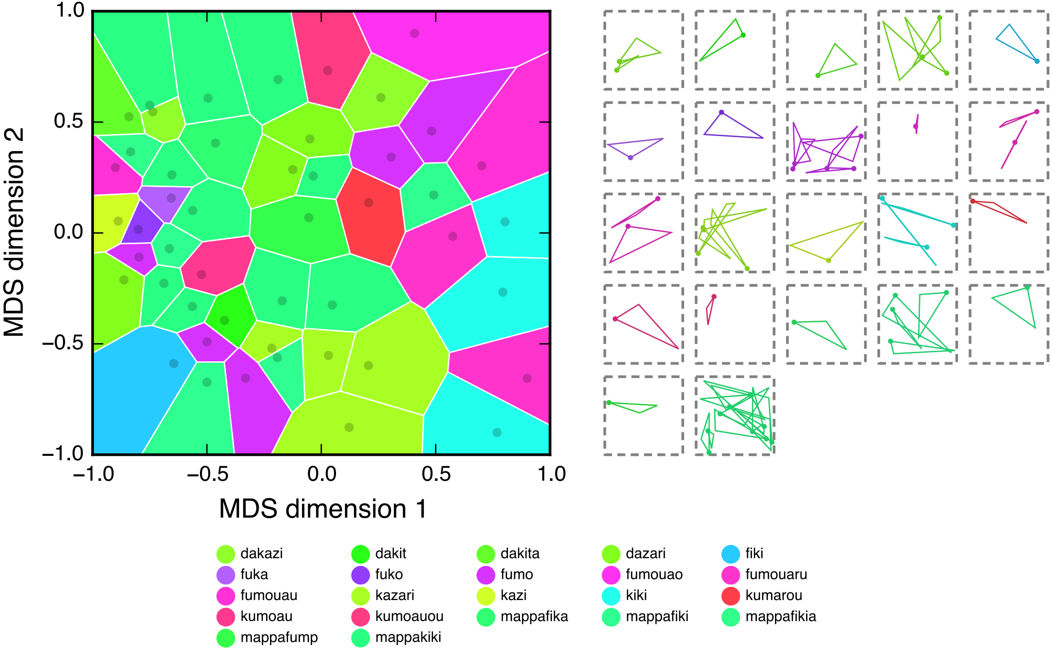

Experiment 1 Chain C Generation 5

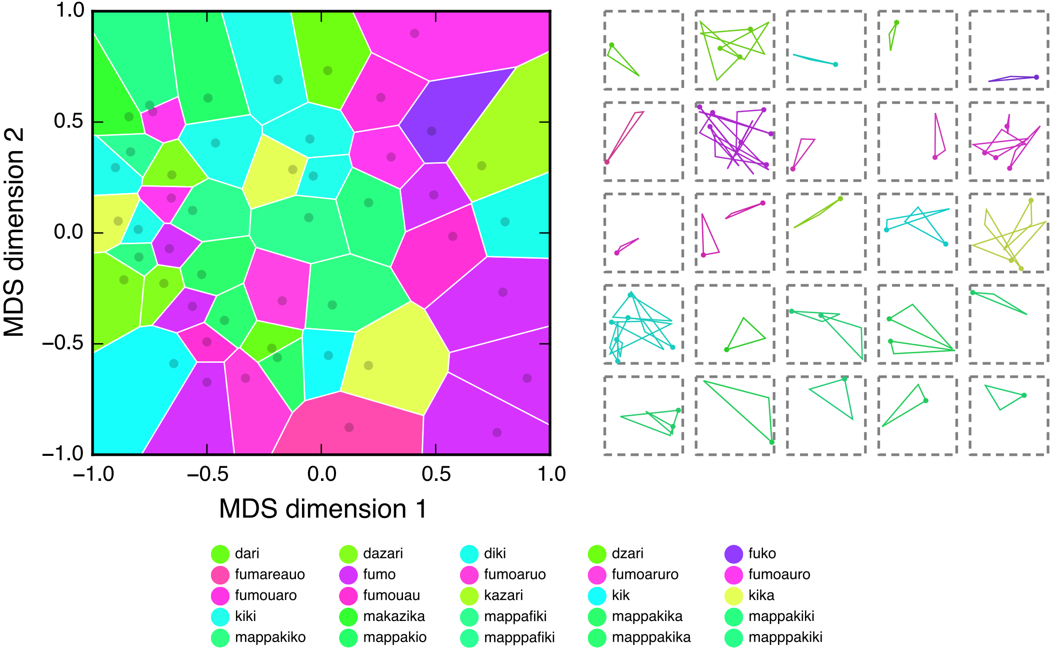

Experiment 1 Chain C Generation 6

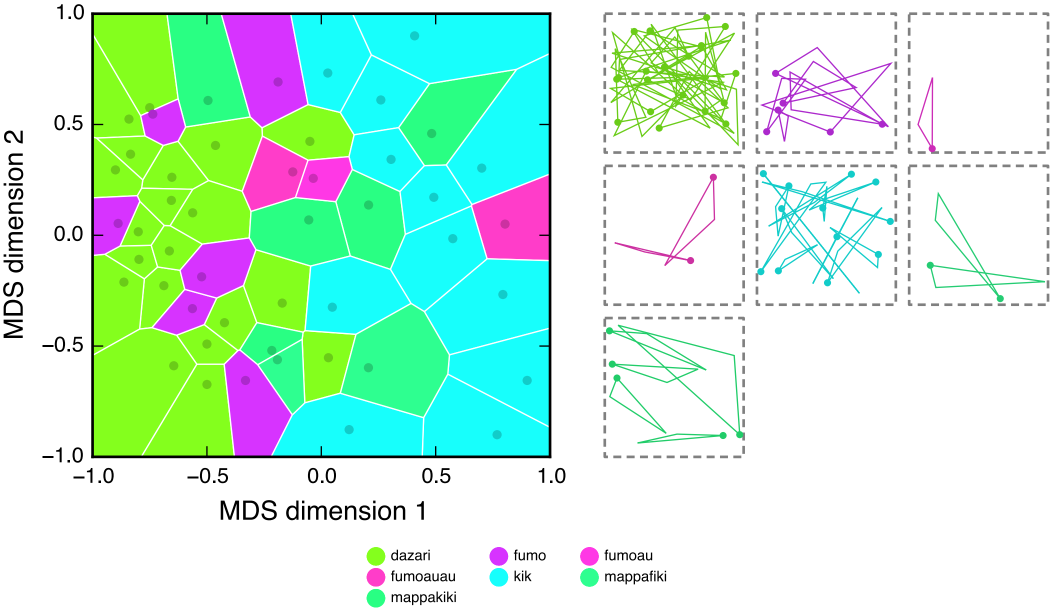

Experiment 1 Chain C Generation 7

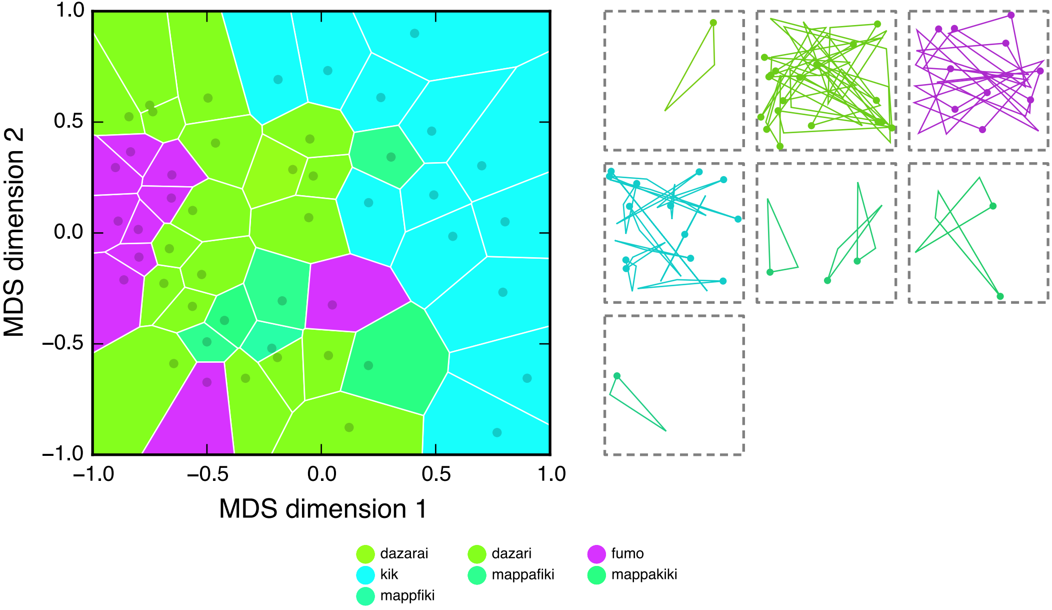

Experiment 1 Chain C Generation 8

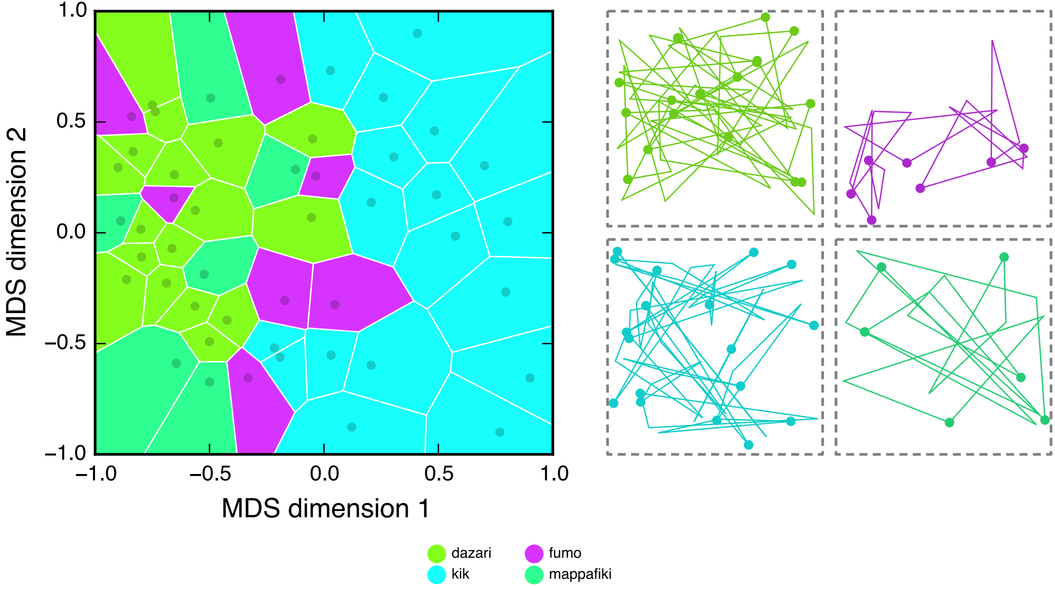

Experiment 1 Chain C Generation 9

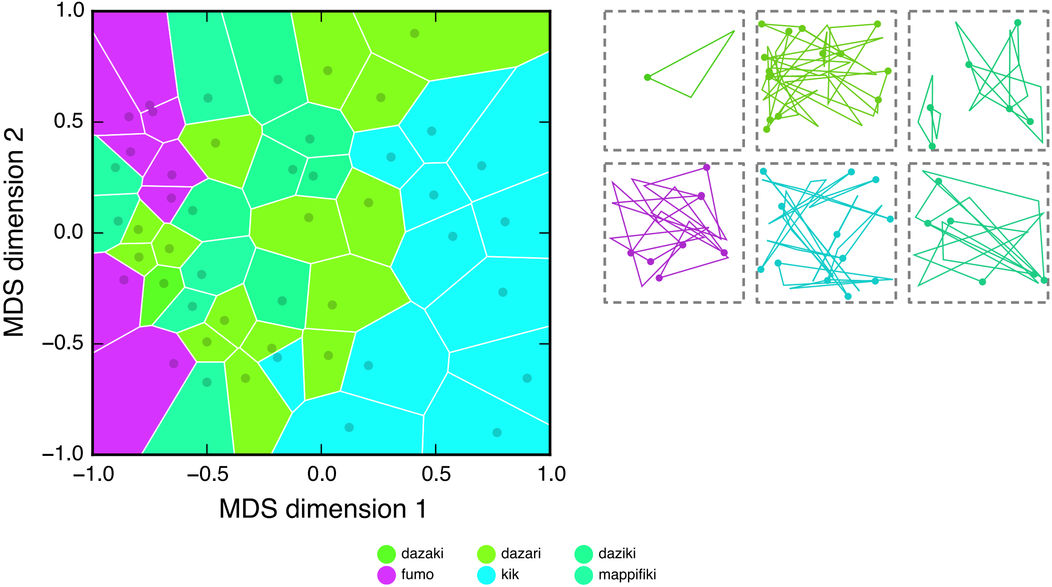

Experiment 1 Chain C Generation 10

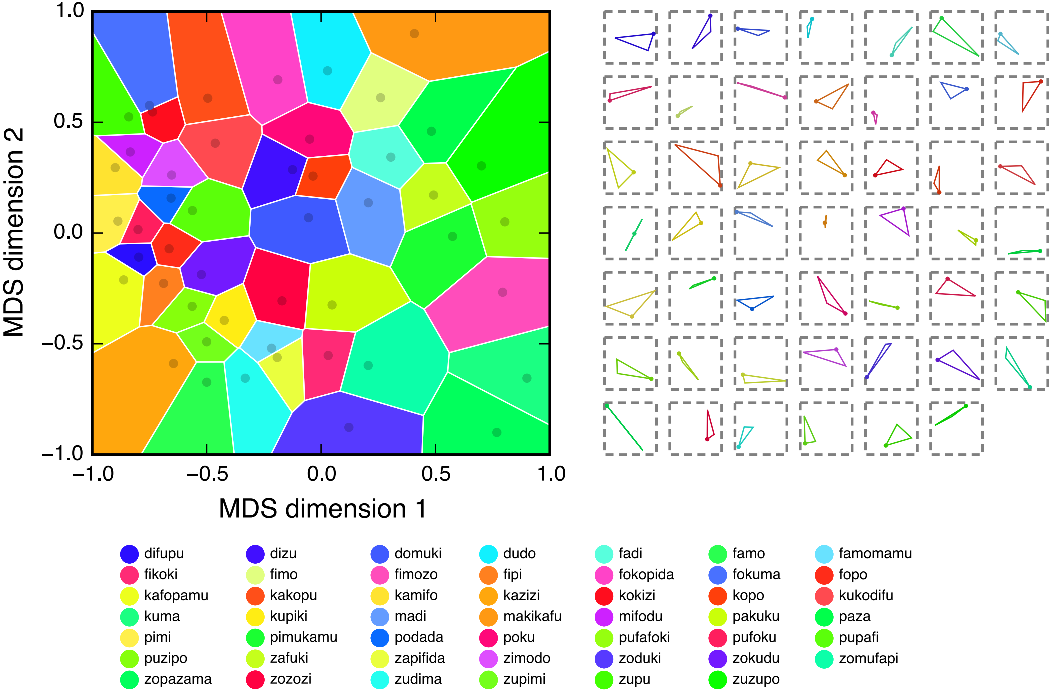

Experiment 1 Chain D Generation 0

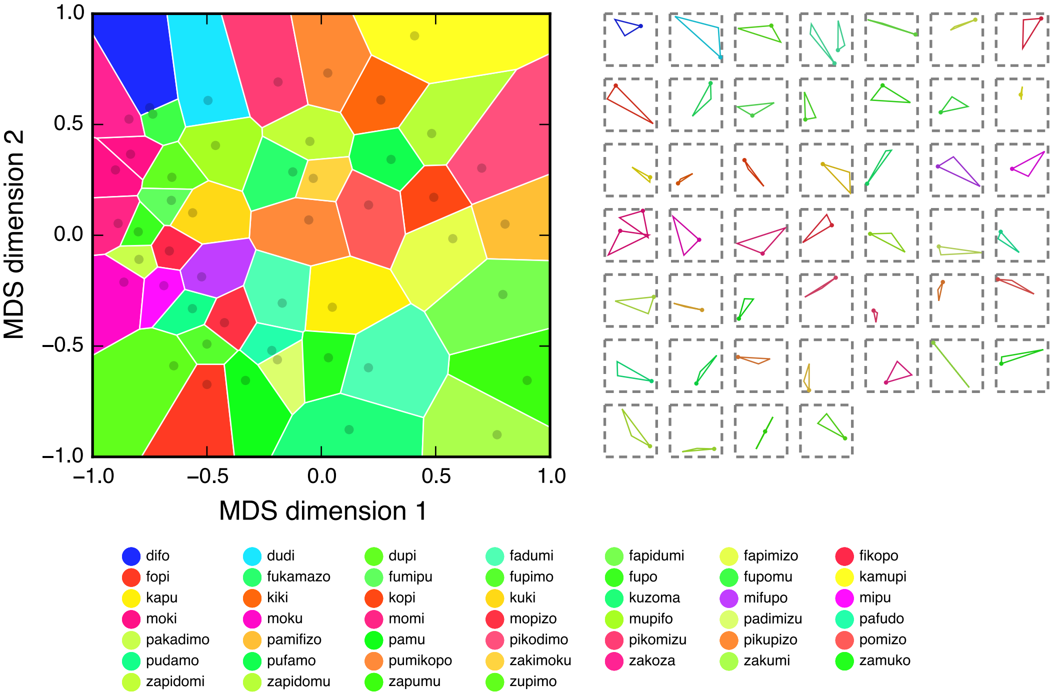

Experiment 1 Chain D Generation 1

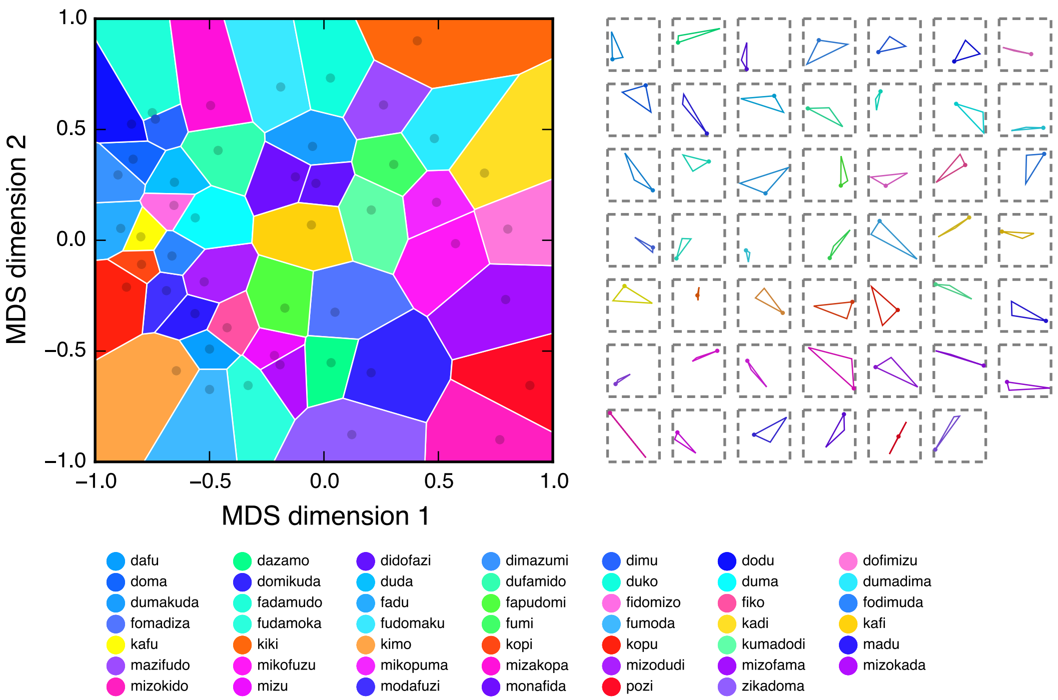

Experiment 1 Chain D Generation 2

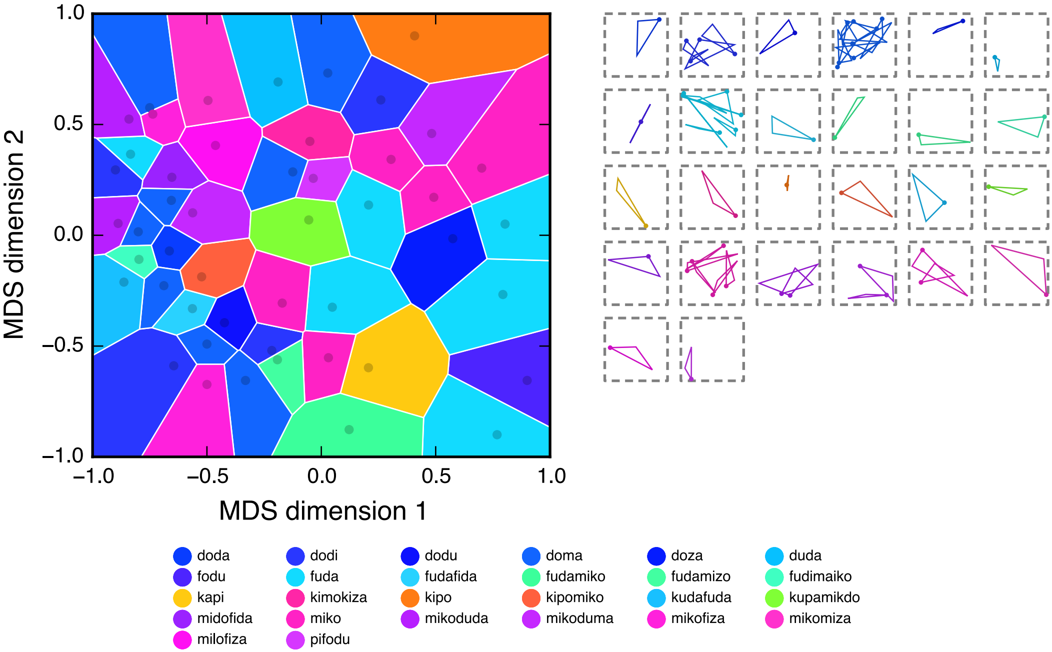

Experiment 1

Chain D

Generation 3

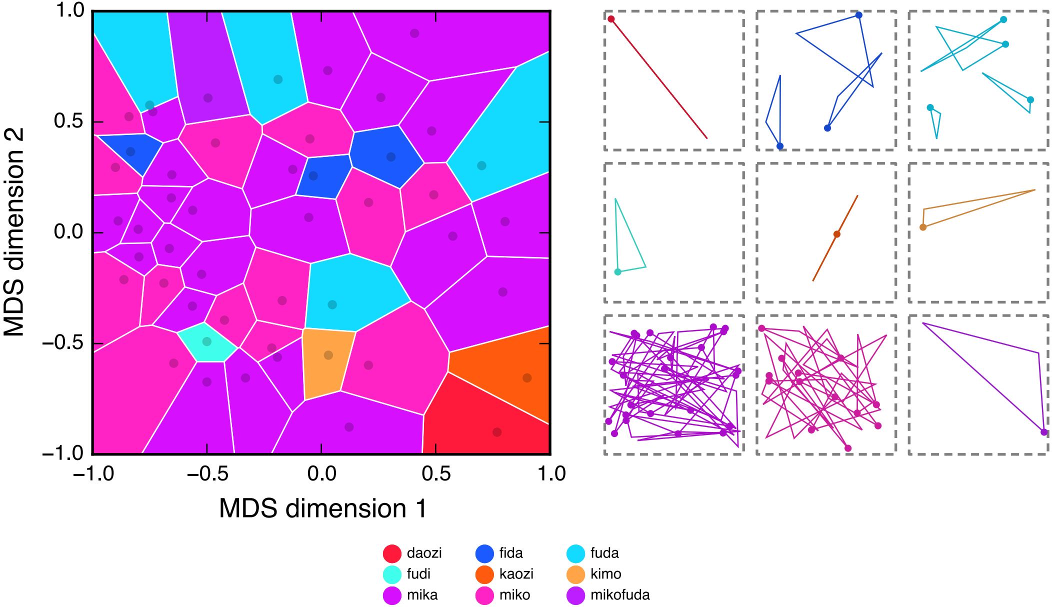

Experiment 1 Chain D Generation 4

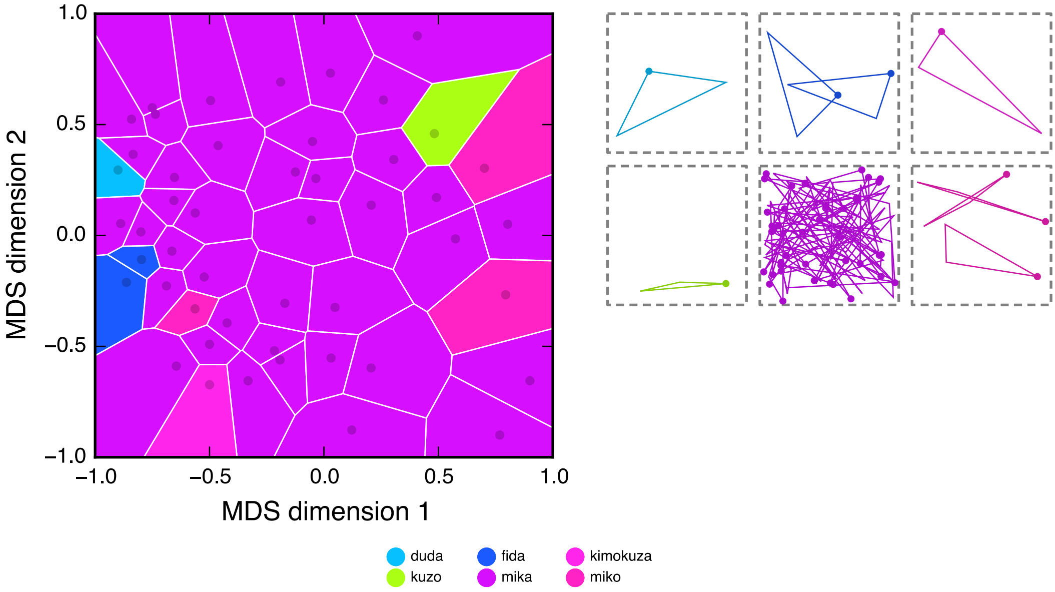

Experiment 1 Chain D Generation 5

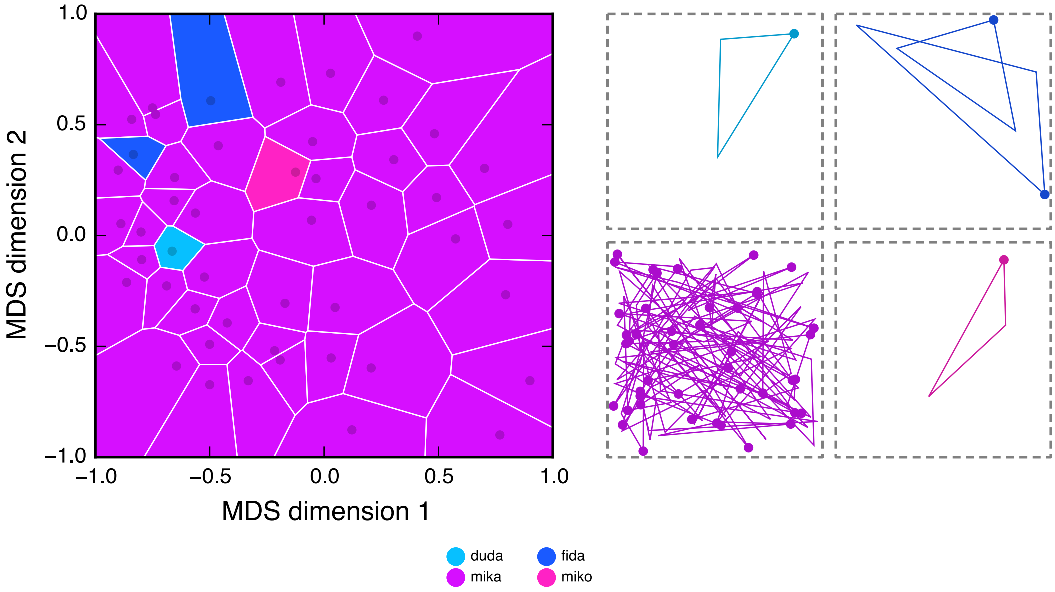

Experiment 1 Chain D Generation 6

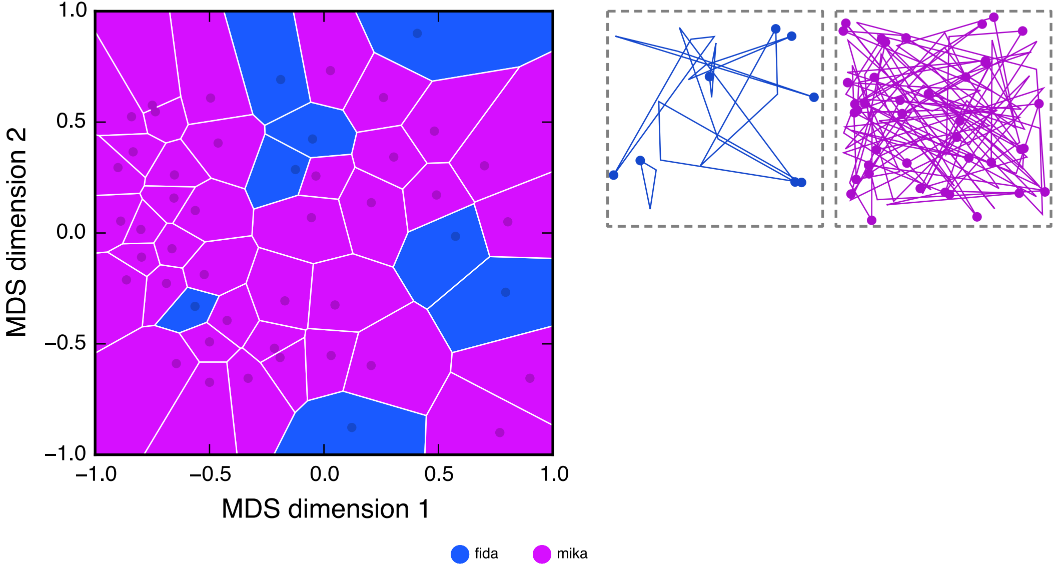

Experiment 1 Chain D Generation 7

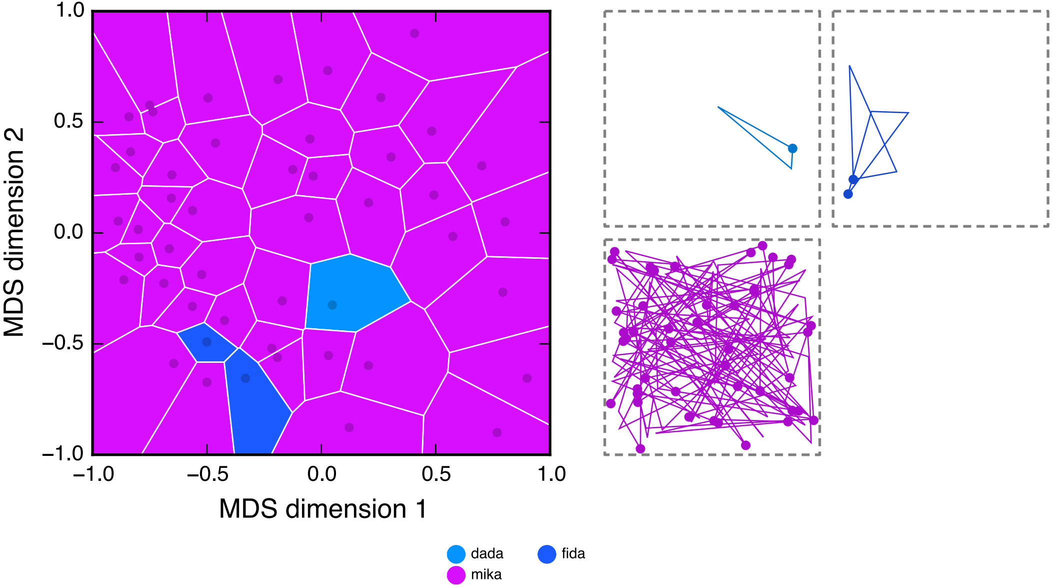

Experiment 1 Chain D Generation 8

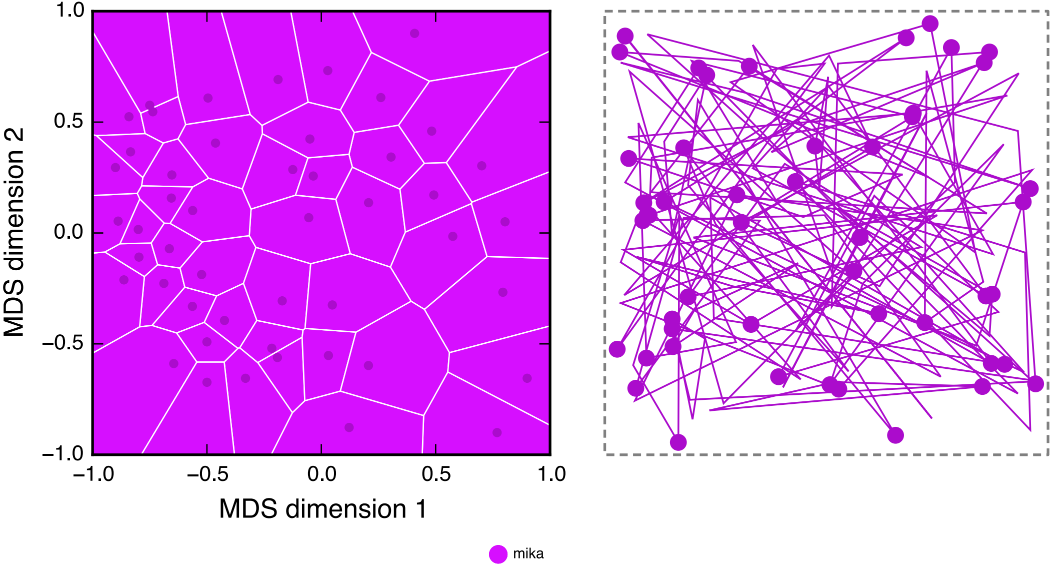

Experiment 1 Chain D Generation 9

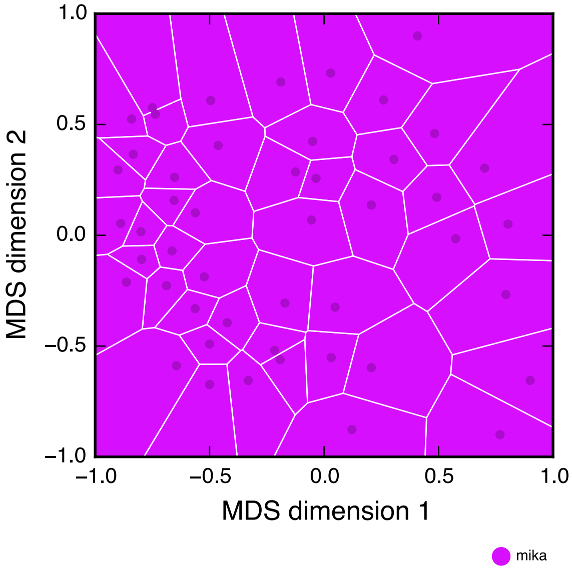

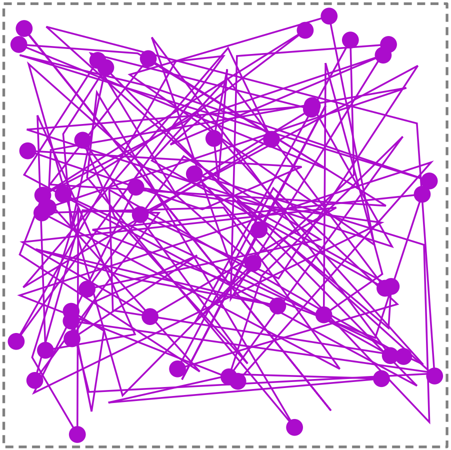

Experiment 1 Chain D Generation 10

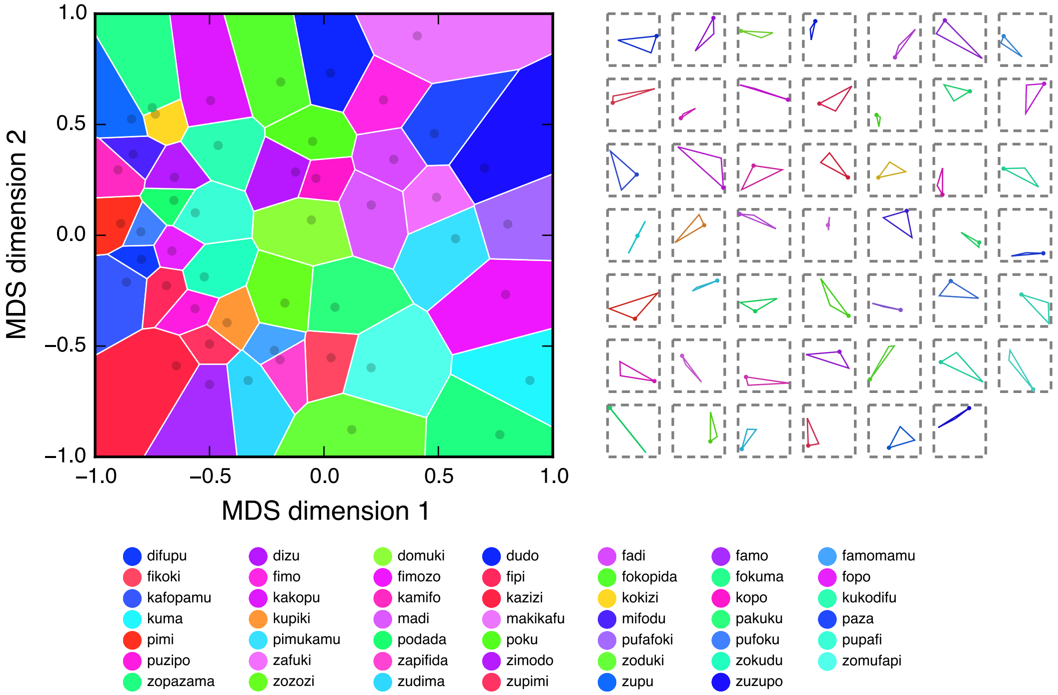

Experiment 2 Chain E Generation 0

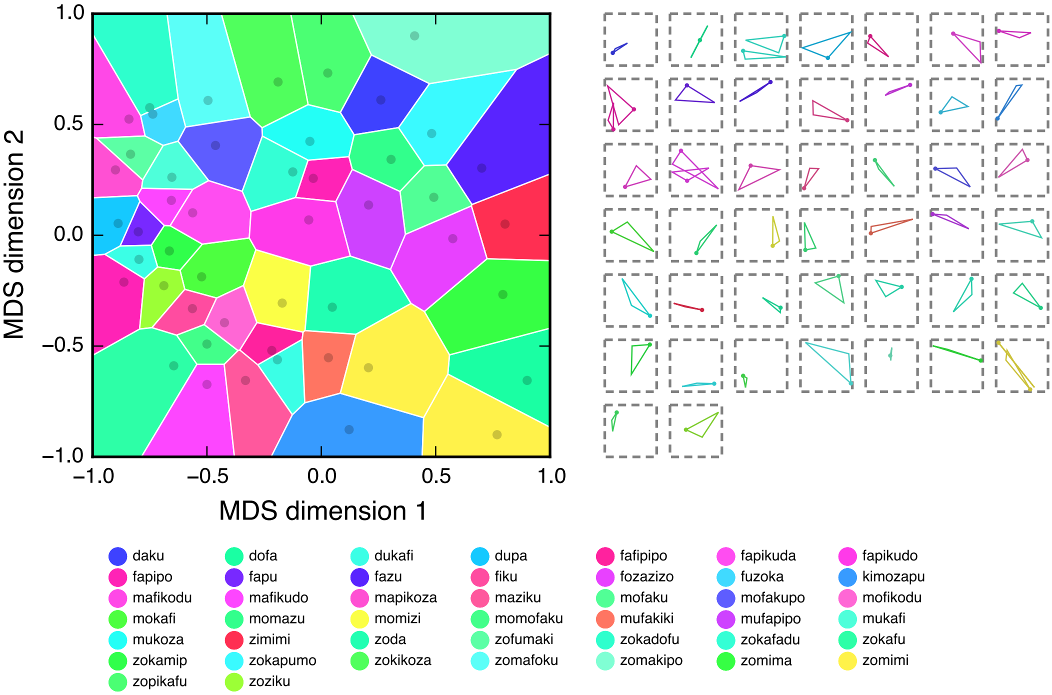

Experiment 2 Chain E Generation 1

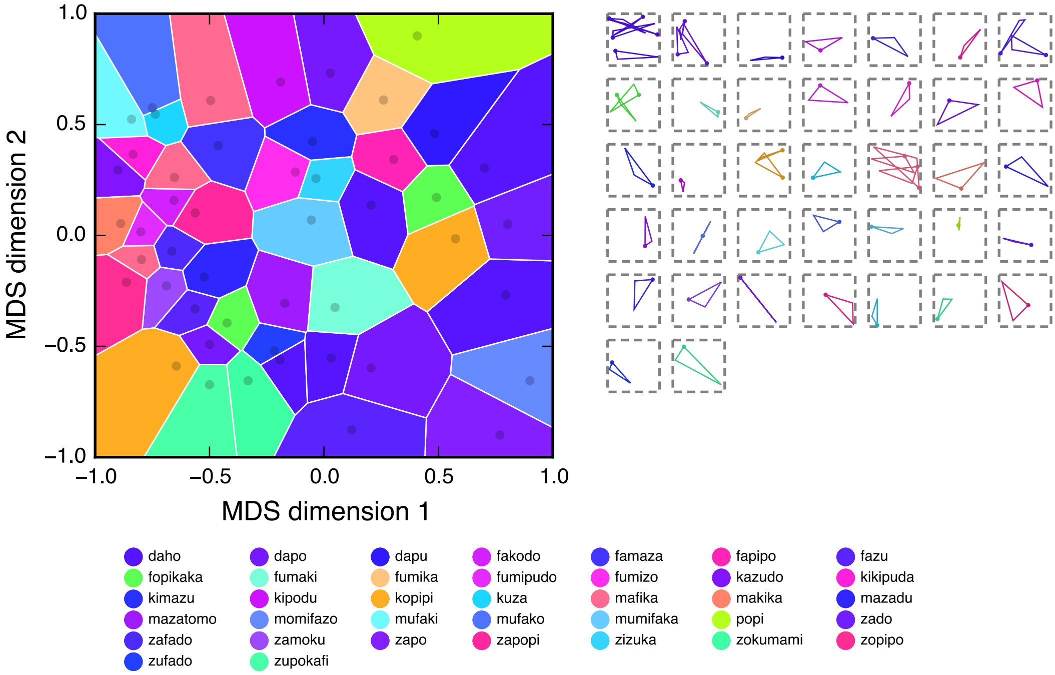

Experiment 2 Chain E Generation 2

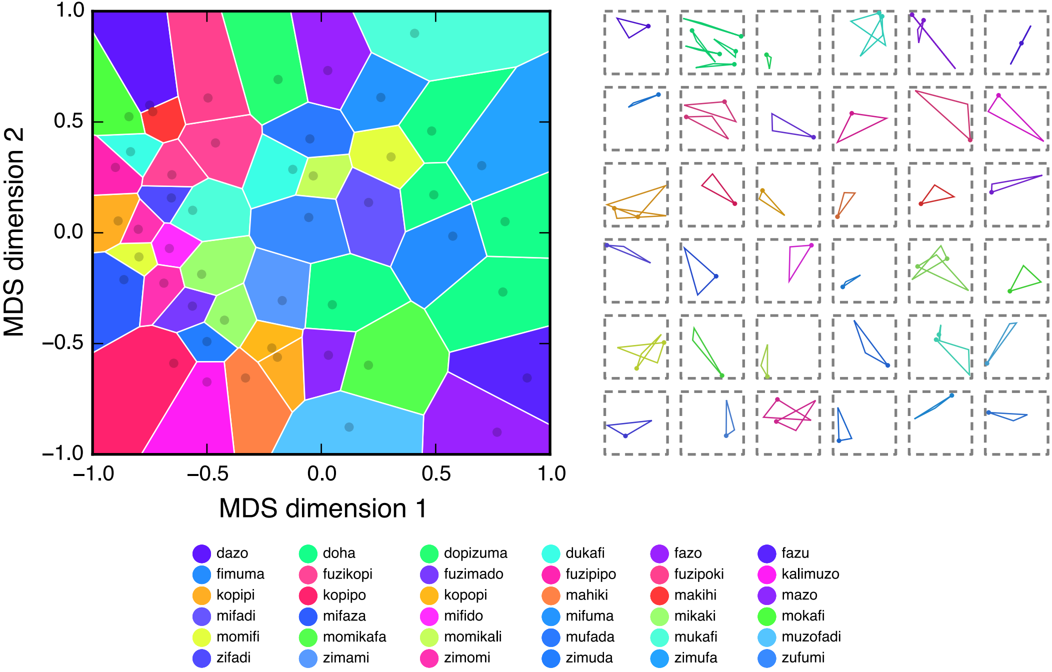

Experiment 2 Chain E Generation 3

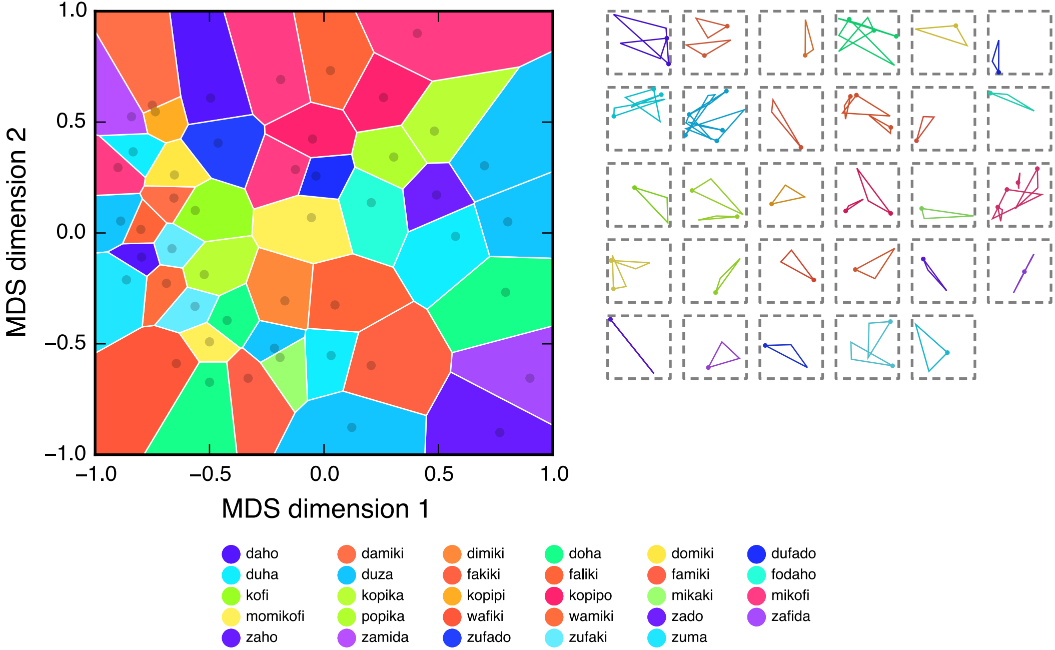

Experiment 2 Chain E Generation 4

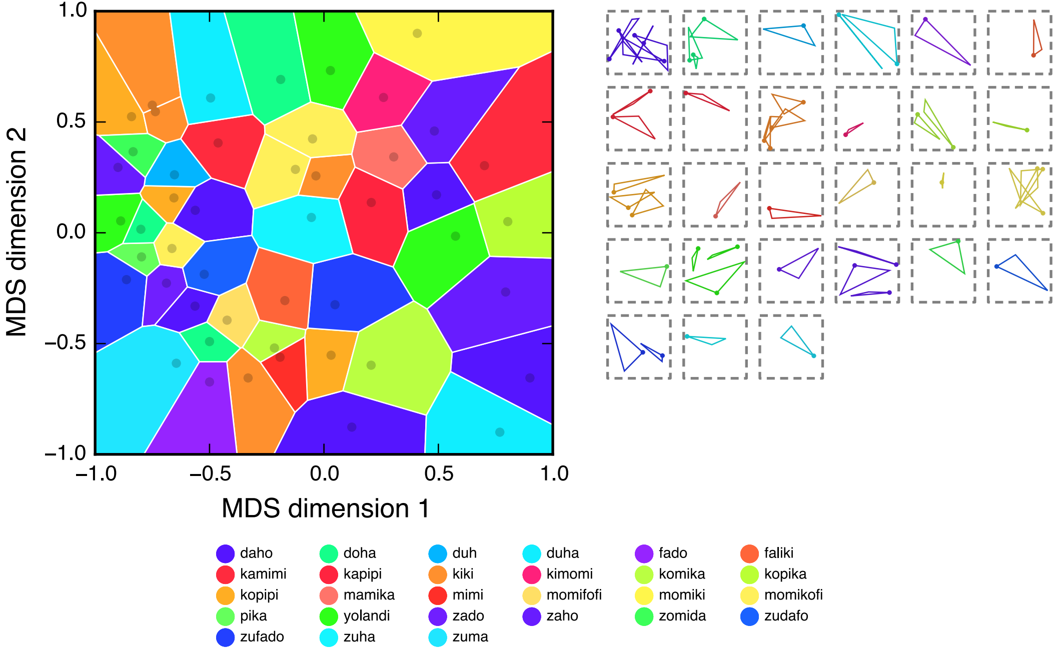

Experiment 2 Chain E Generation 5

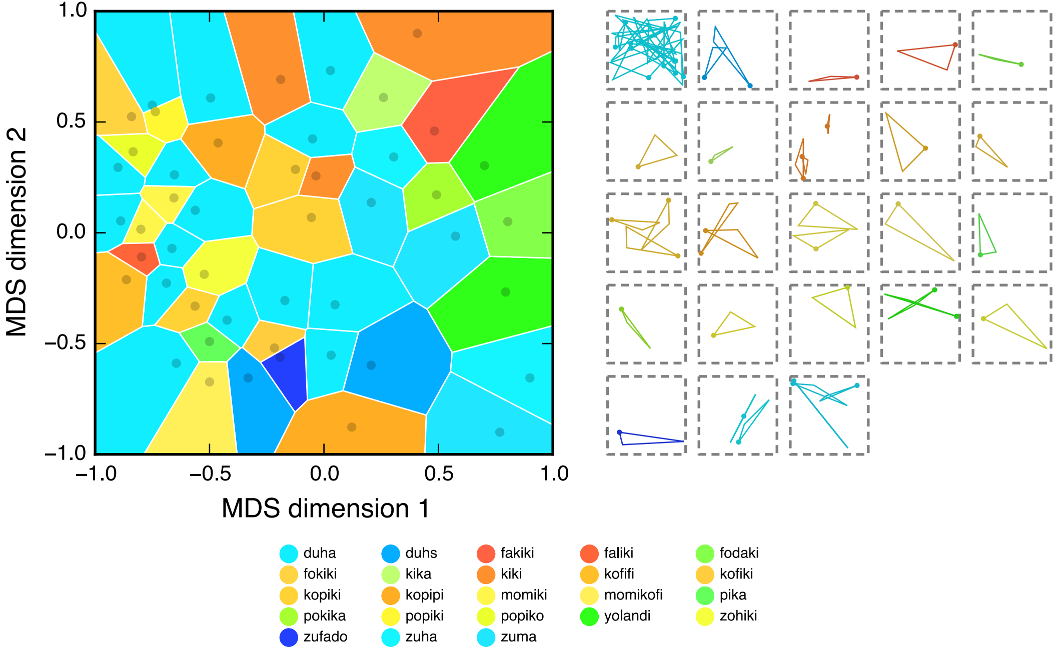

Experiment 2 Chain E Generation 6

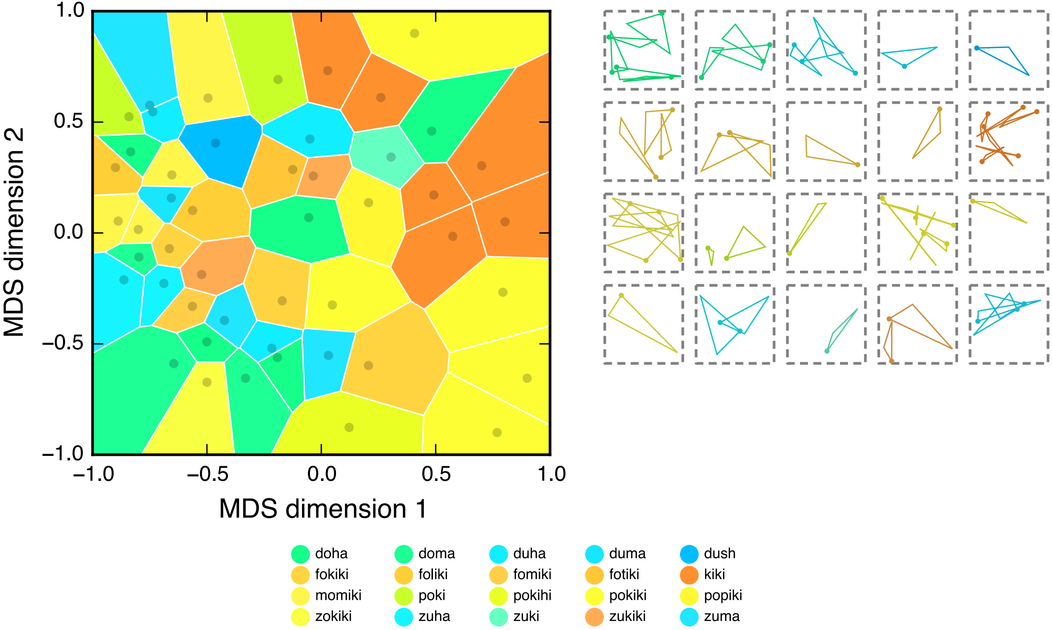

Experiment 2 Chain E Generation 7

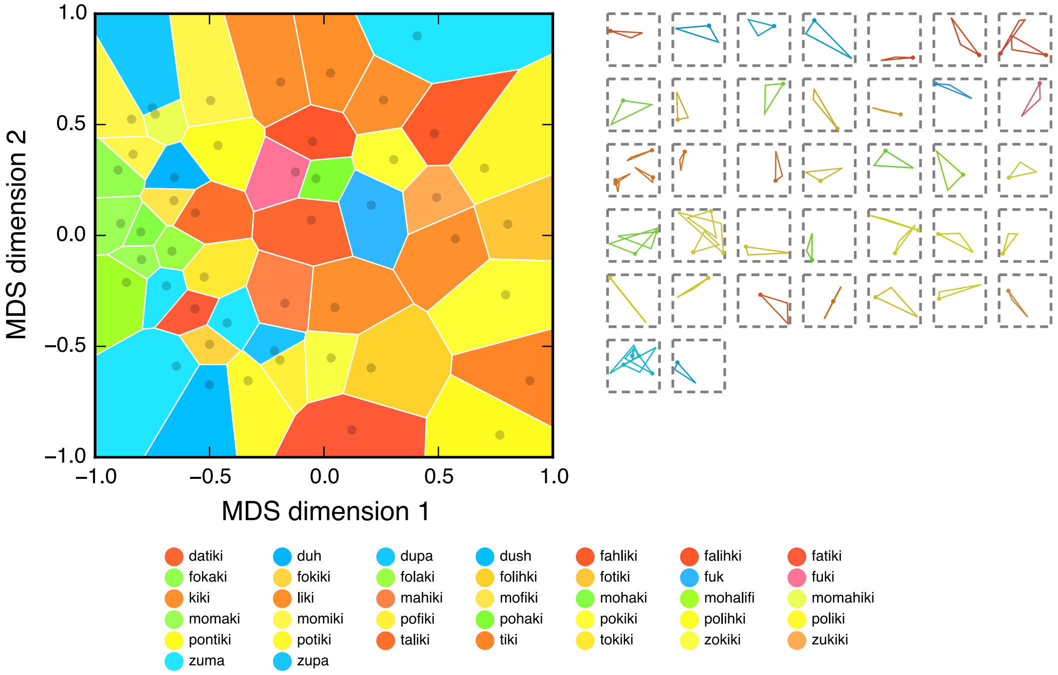

Experiment 2 Chain E Generation 8

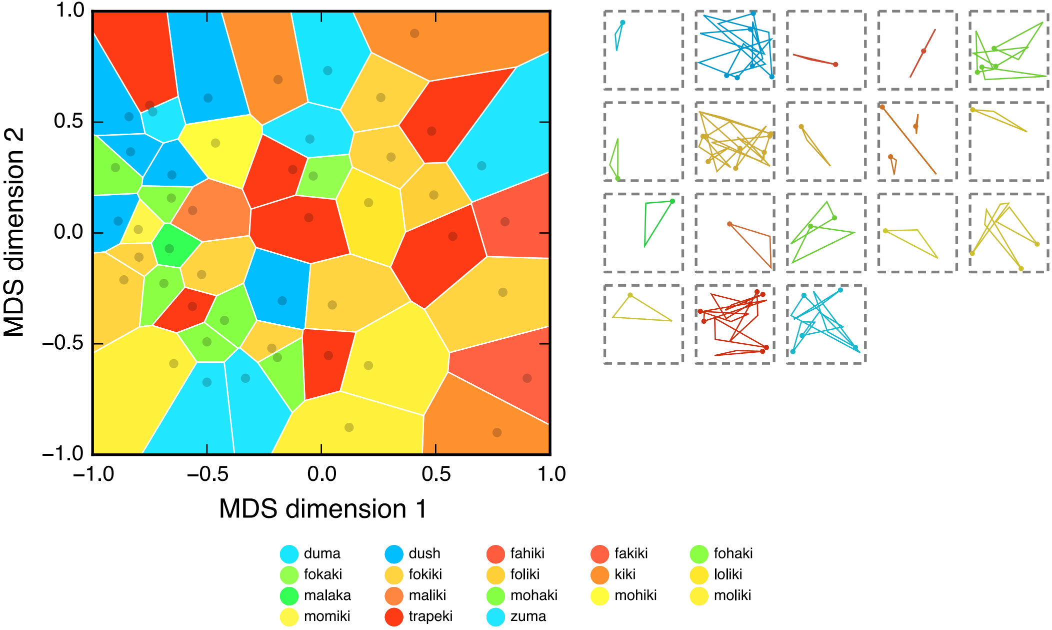

Experiment 2 Chain E Generation 9

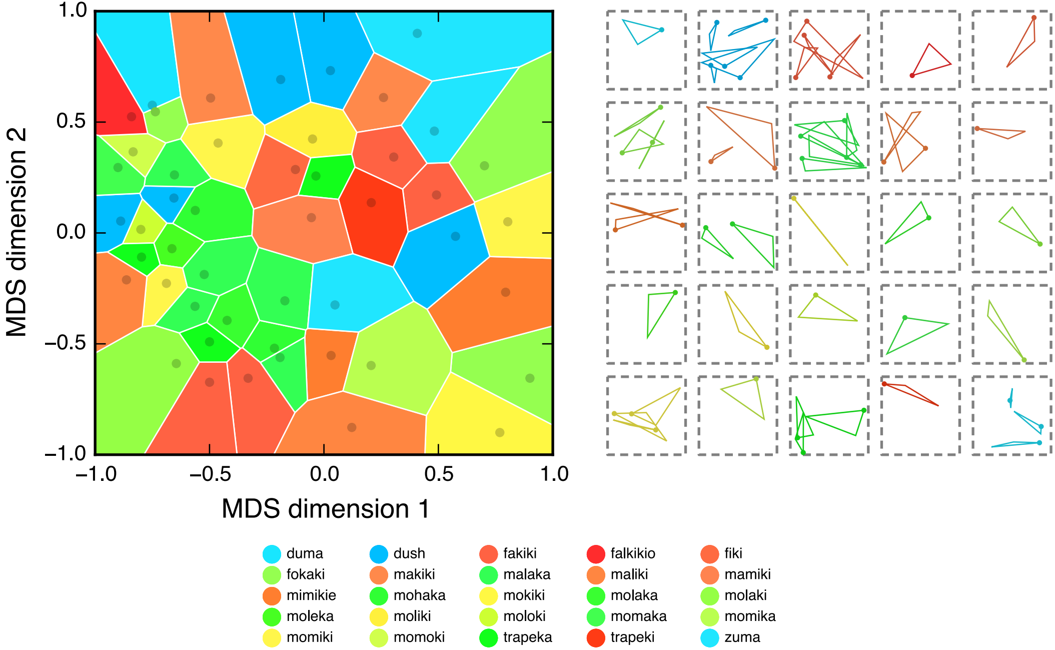

Experiment 2 Chain E Generation 10

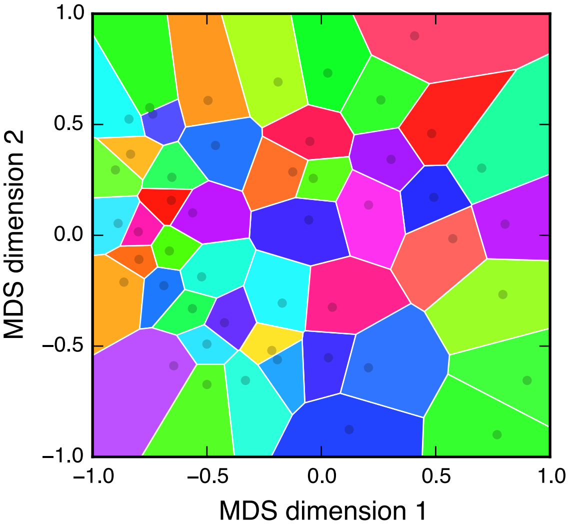

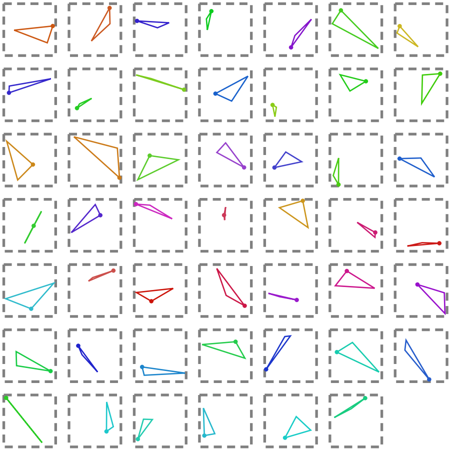

difupu	dizu	domuki	dudo	fadi	famo	famomamu
fikoki	fimo	fimozo	fipi	fokopida	fokuma	fopo
kafopamu	kakopu	kamifo	kazizi	kokizi	kopo	kukodifu
kuma	kupiki	madi	makikafu	mifodu	pakuku	paza
pimi	pimukamu	podada	poku	pufafoki	pufoku	pupafi
puzipo	zafuki	zapifida	zimodo	zoduki	zokudu	zomufapi
zopazama	zozozi	zudima	zupimi	zupu	zuzupo	

Experiment 2 Chain F Generation 0

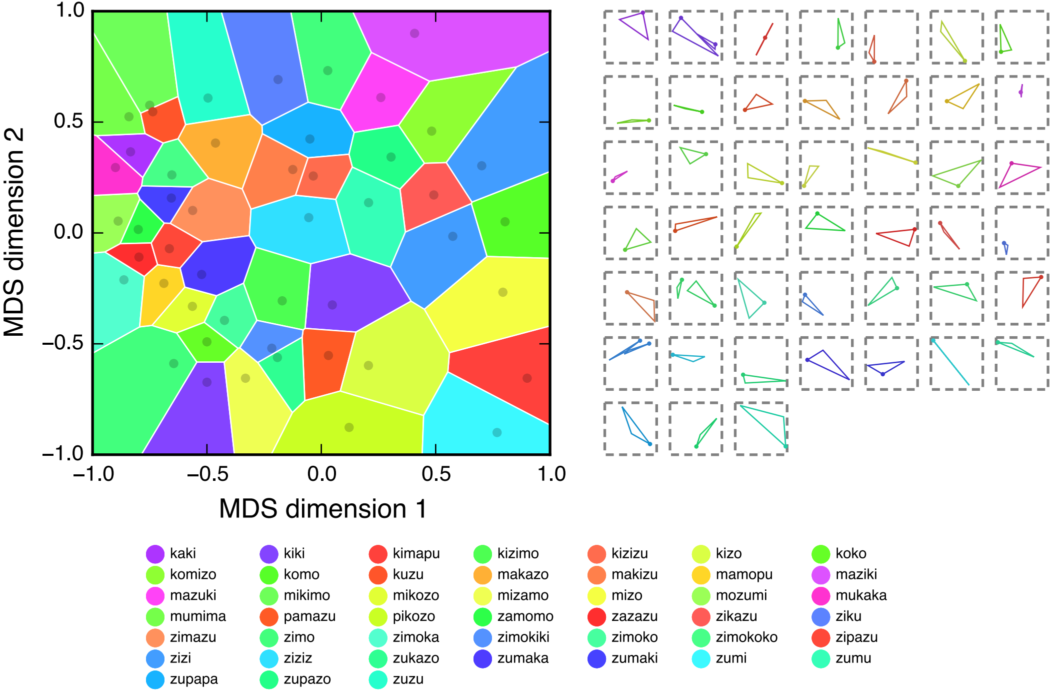

Experiment 2 Chain F Generation 1

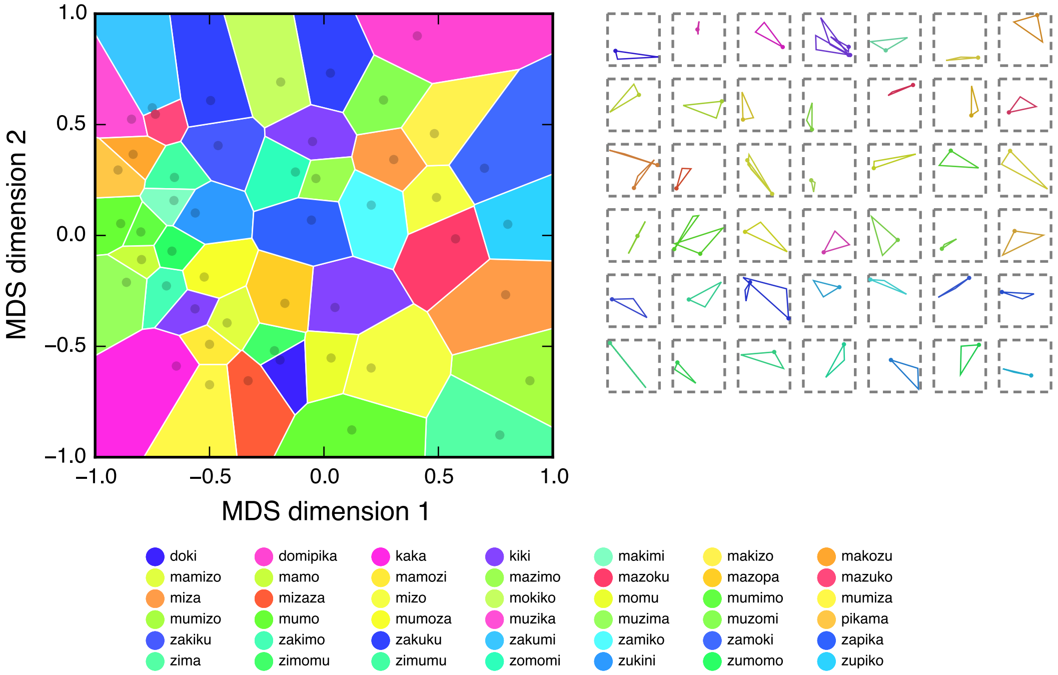

Experiment 2 Chain F Generation 2

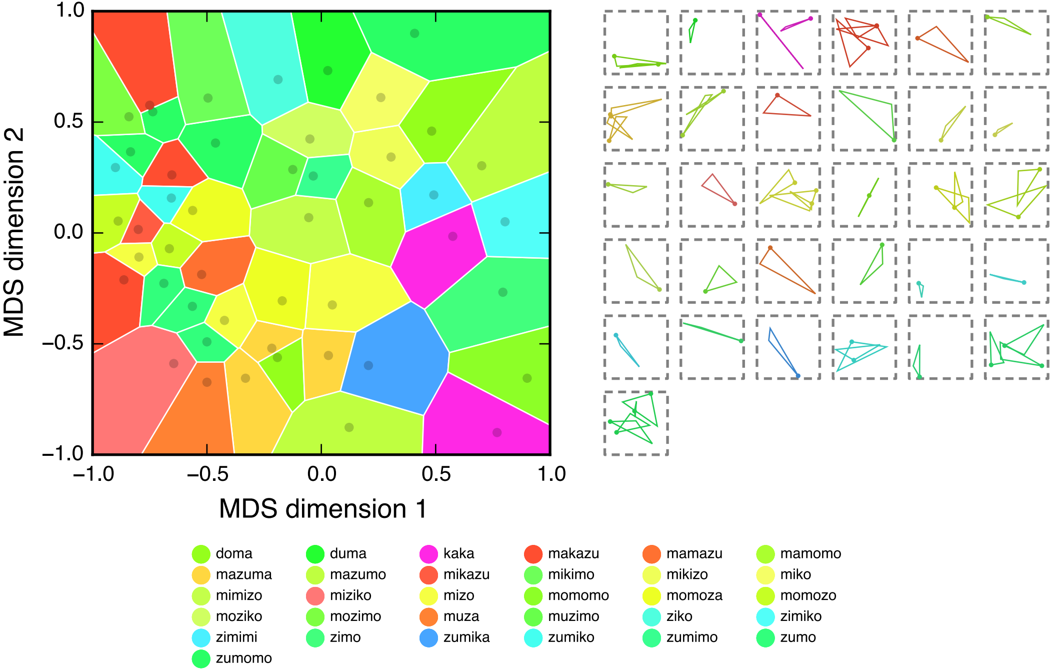

Experiment 2 Chain F Generation 3

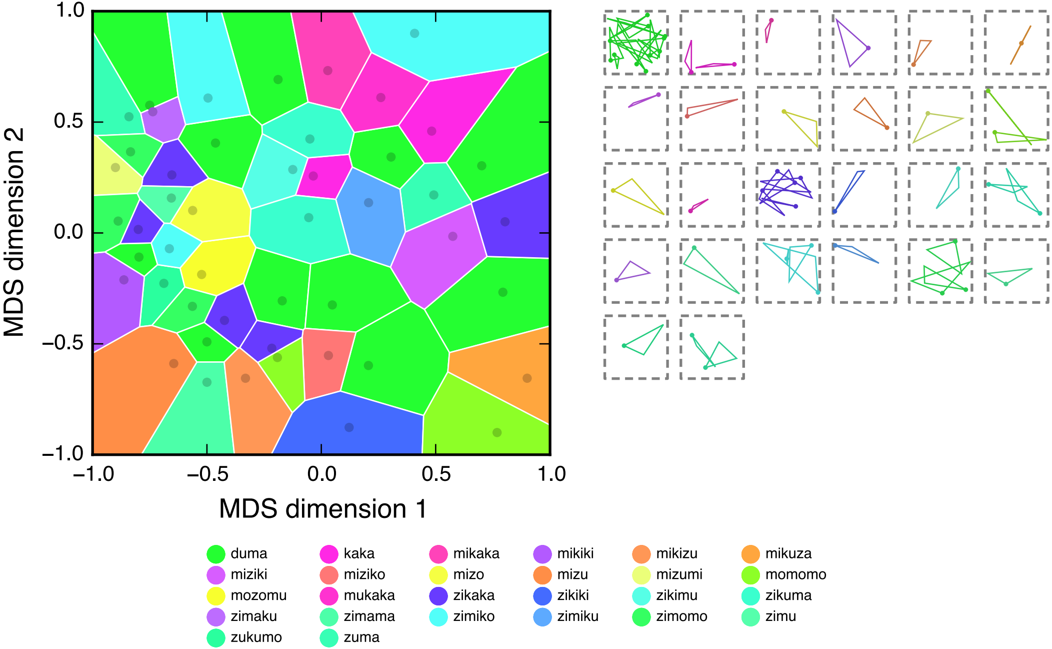

Experiment 2 Chain F Generation 4

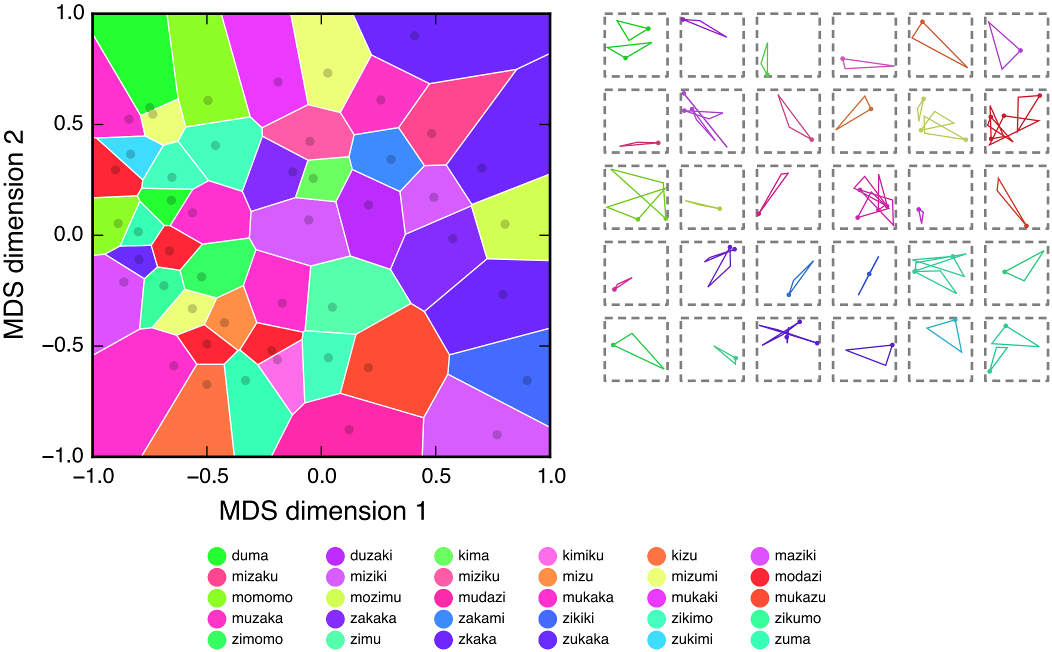

Experiment 2 Chain F Generation 5

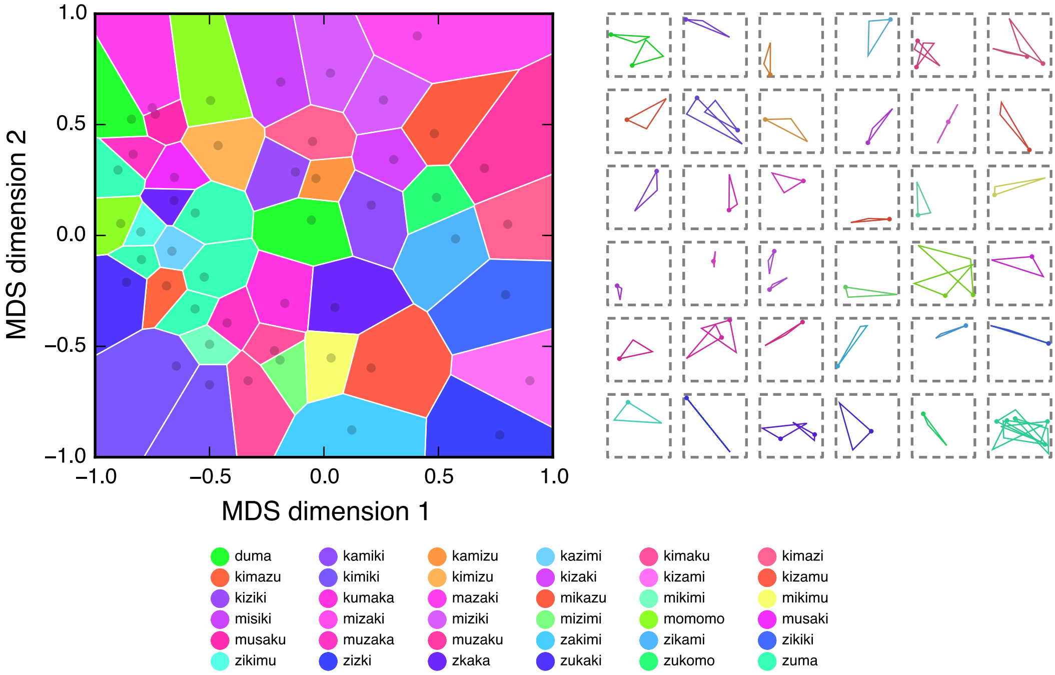

Experiment 2 Chain F Generation 6

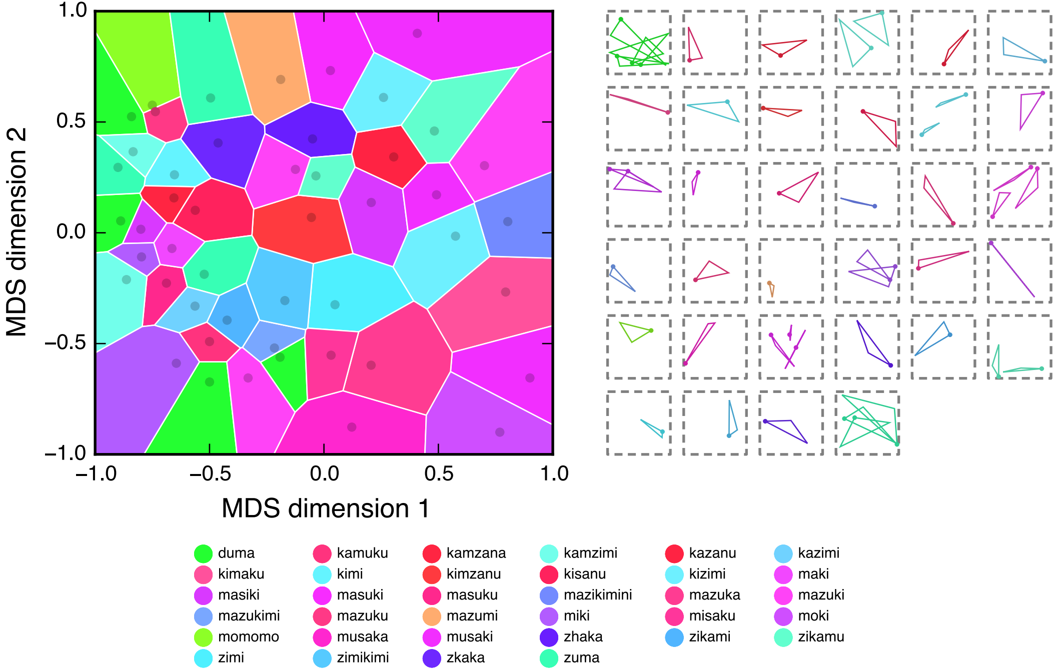

Experiment 2 Chain F Generation 7

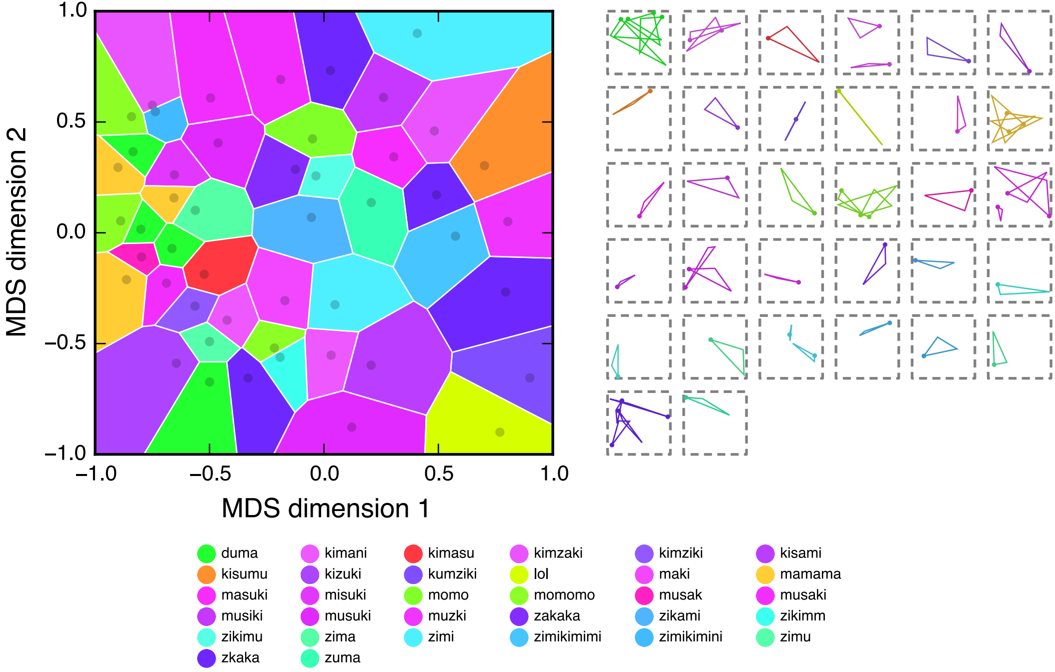

Experiment 2 Chain F Generation 8

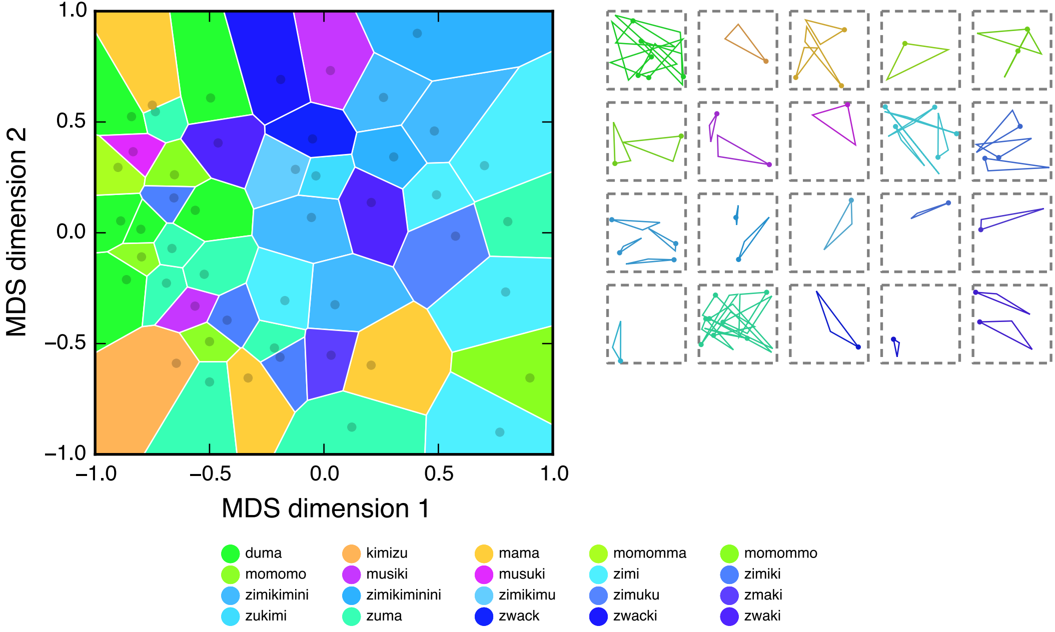

Experiment 2 Chain F Generation 9

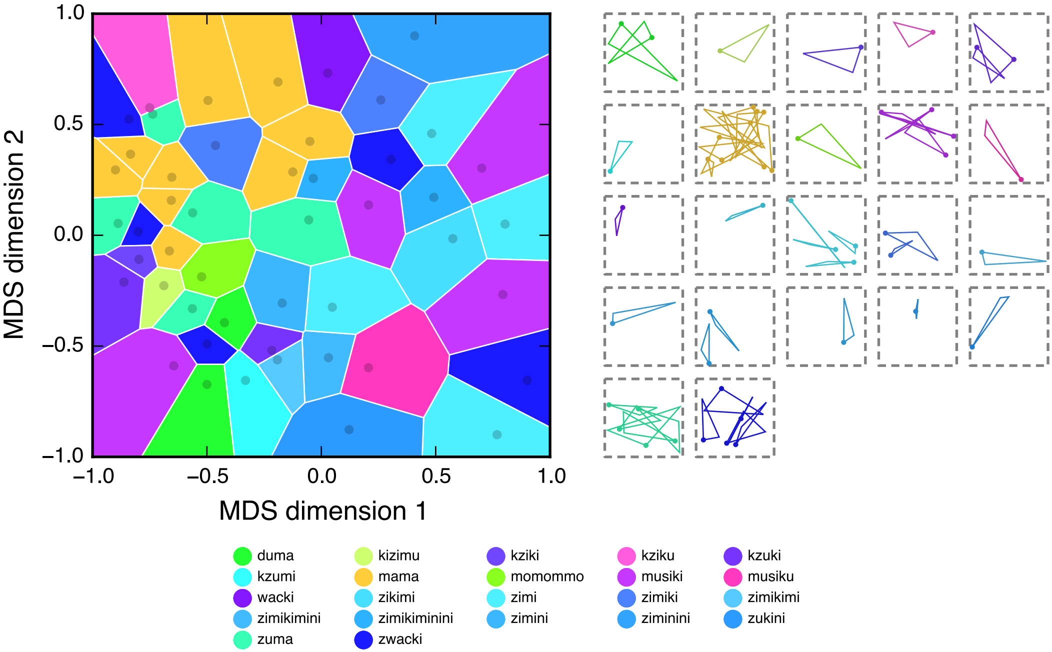

Experiment 2 Chain F Generation 10

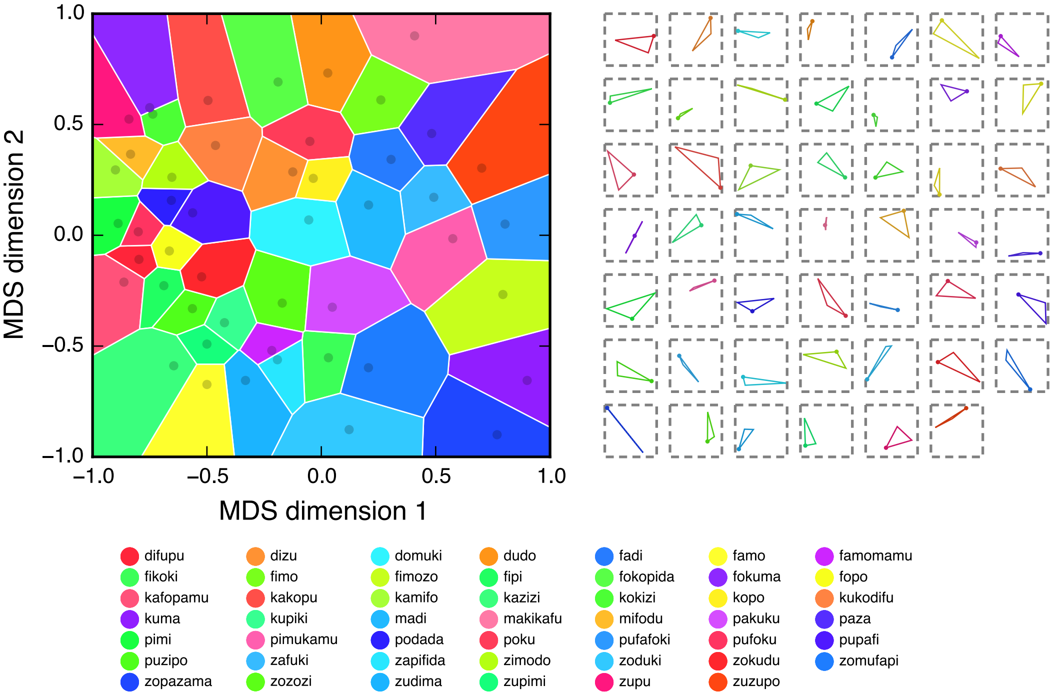

Experiment 2 Chain G Generation 0

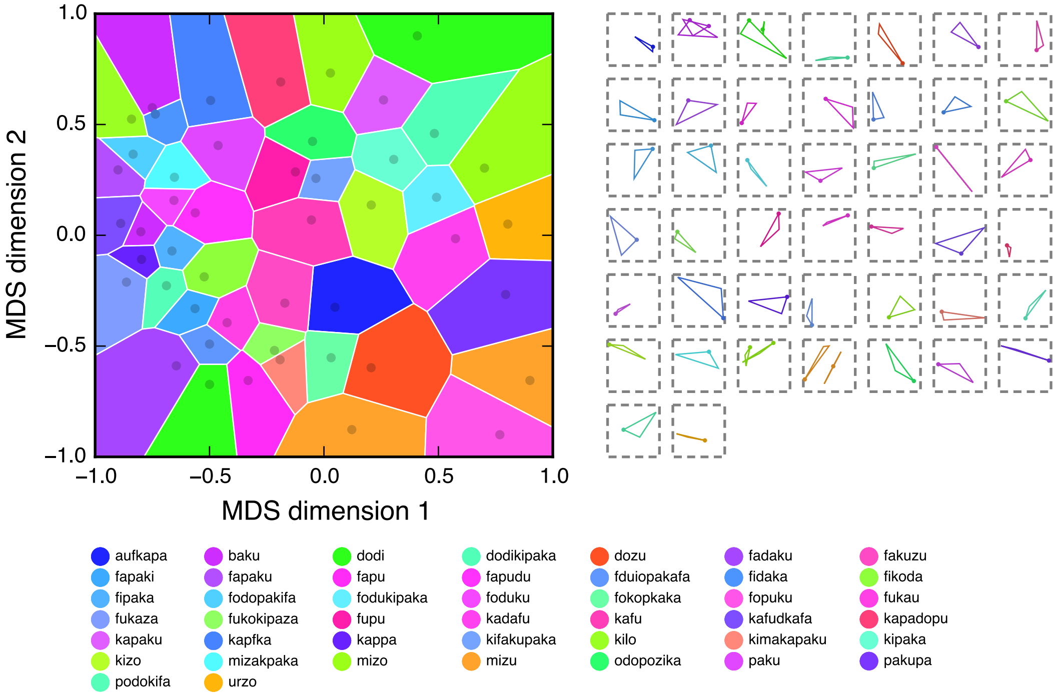

Experiment 2 Chain G Generation 1

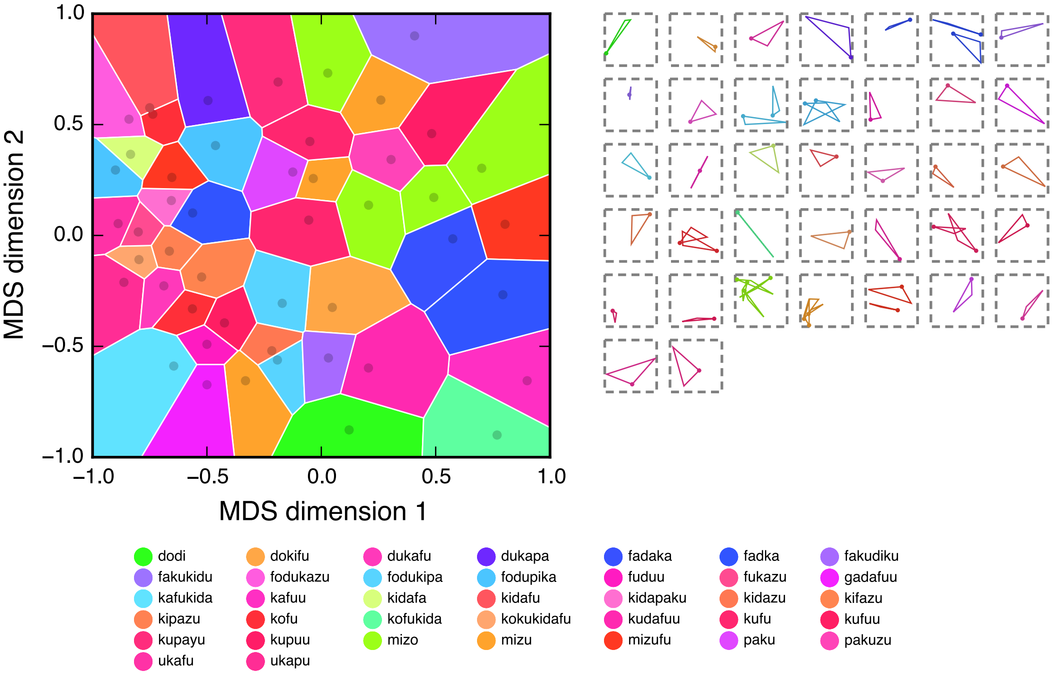

Experiment 2 Chain G Generation 2

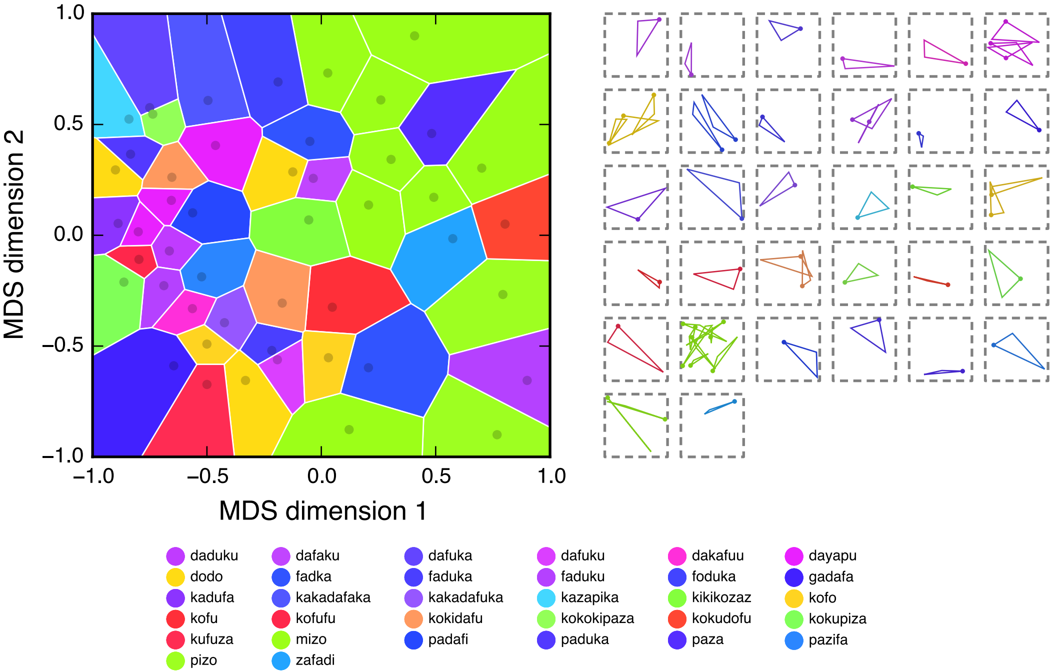

Experiment 2 Chain G Generation 3

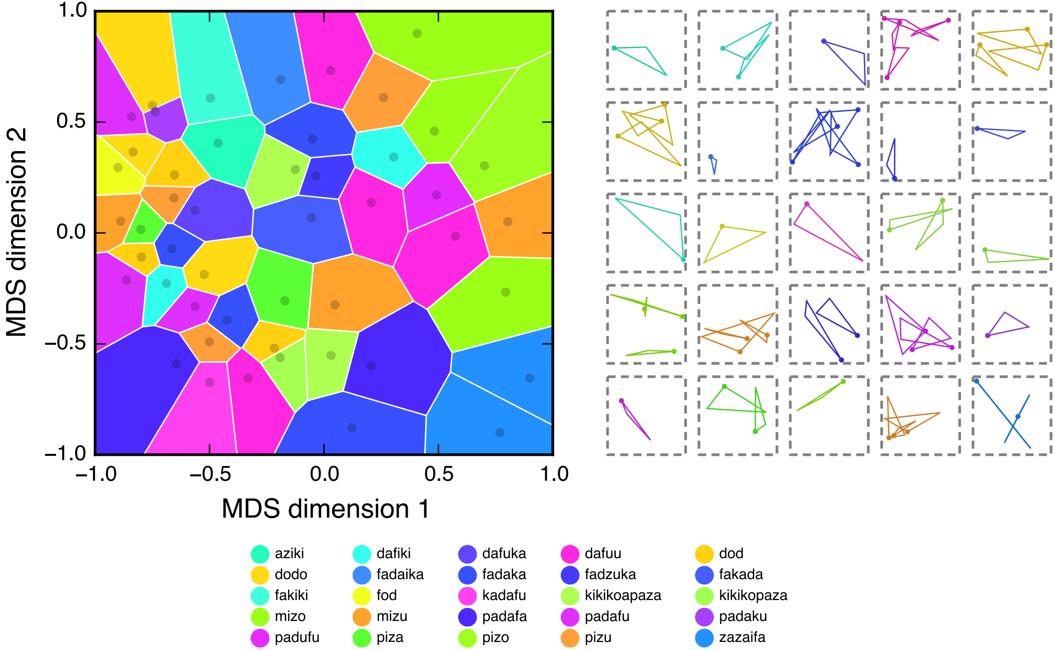

Experiment 2 Chain G Generation 4

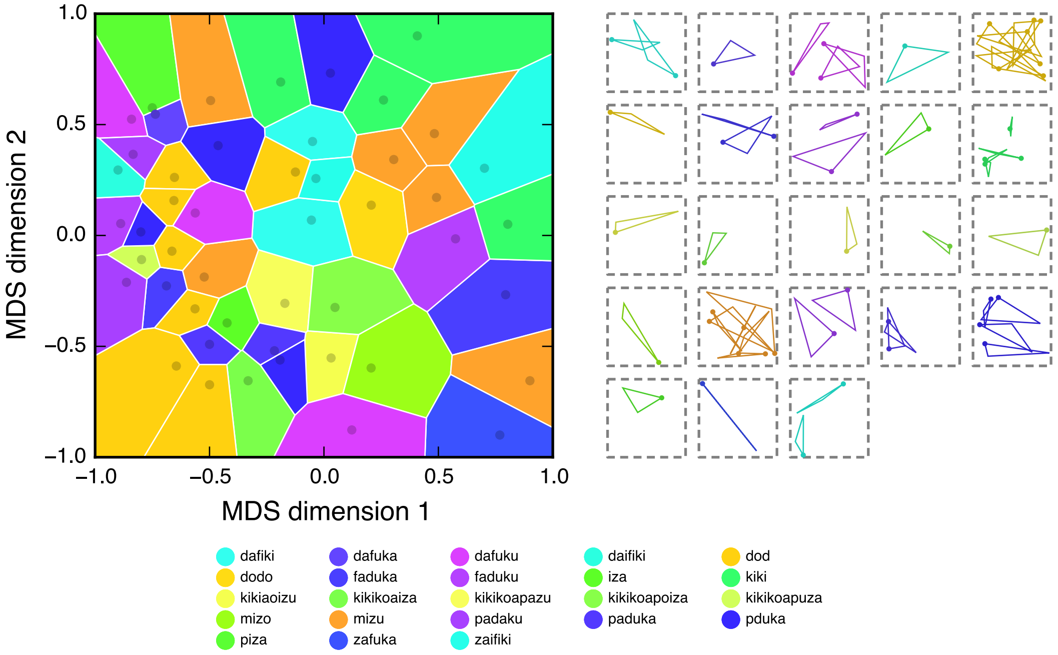

Experiment 2 Chain G Generation 5

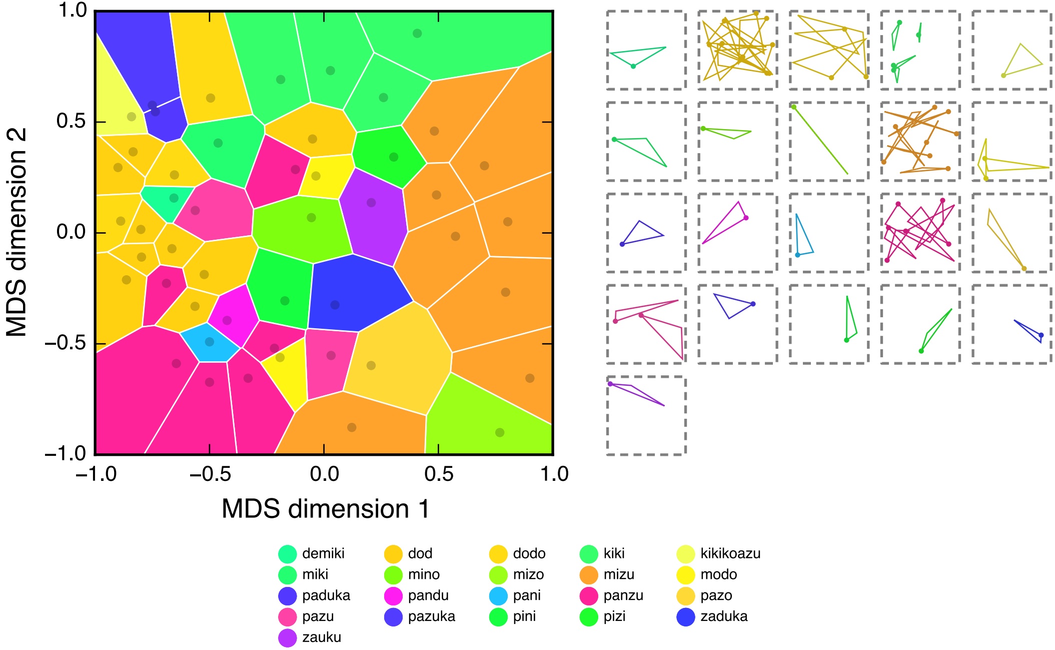

Experiment 2 Chain G Generation 6

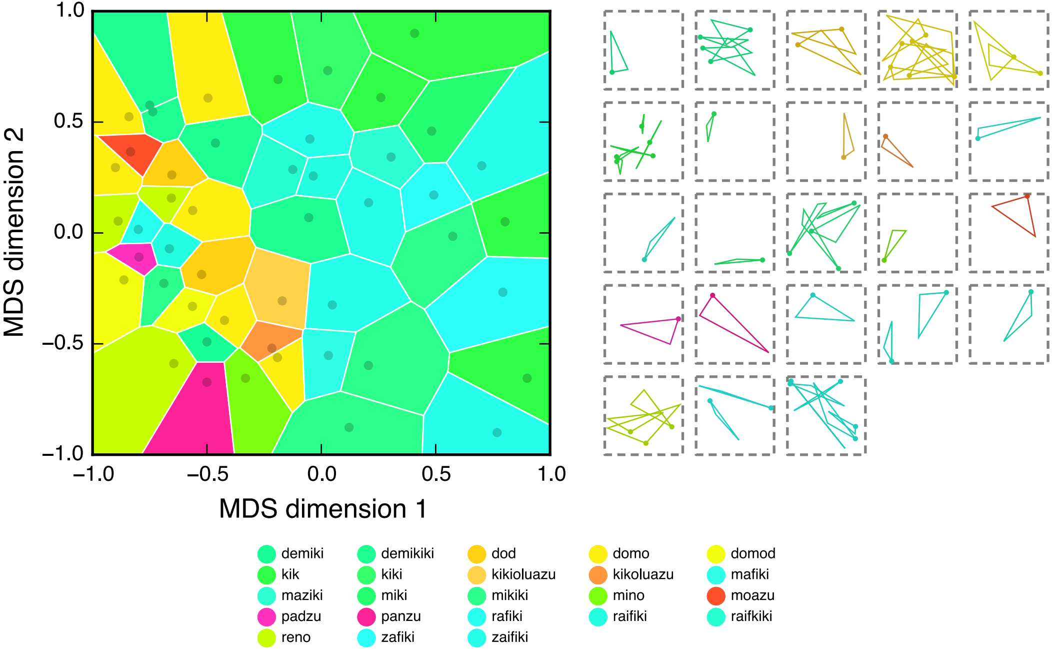

Experiment 2 Chain G Generation 7

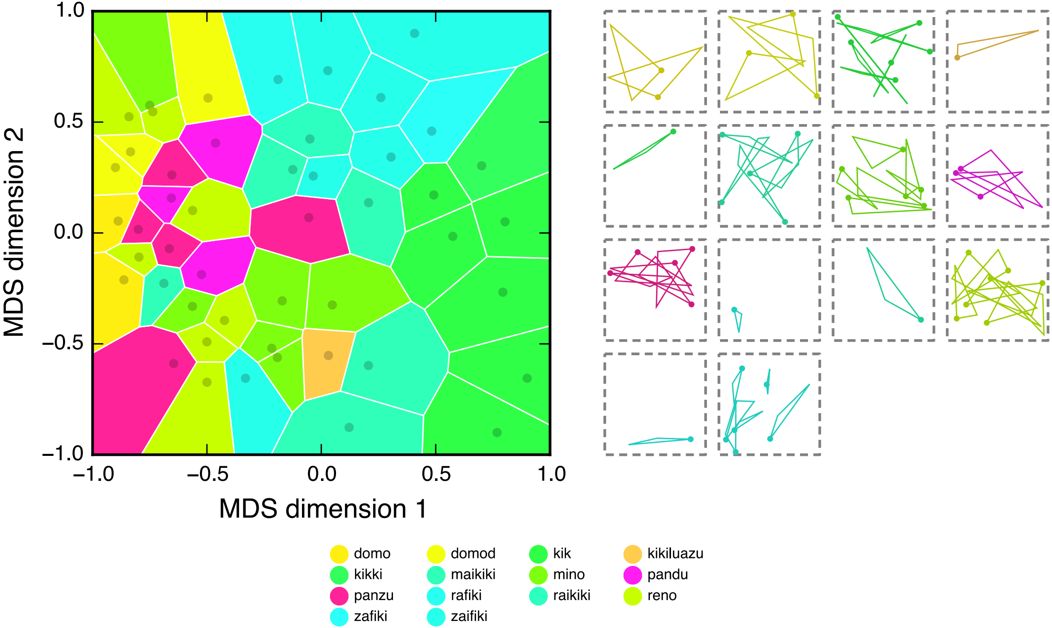

Experiment 2 Chain G Generation 8

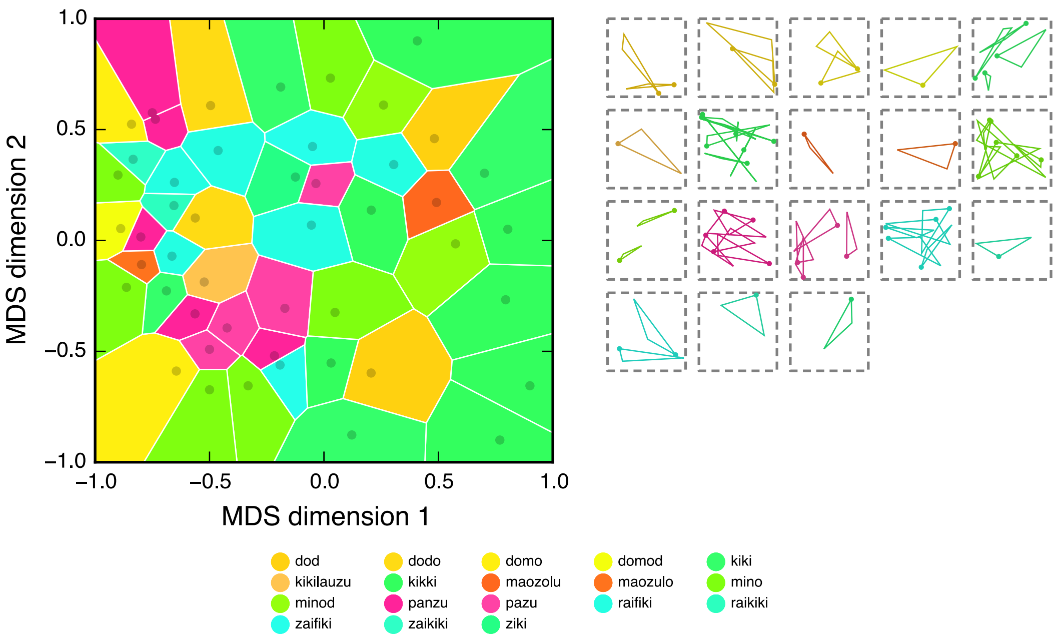

Experiment 2 Chain G Generation 9

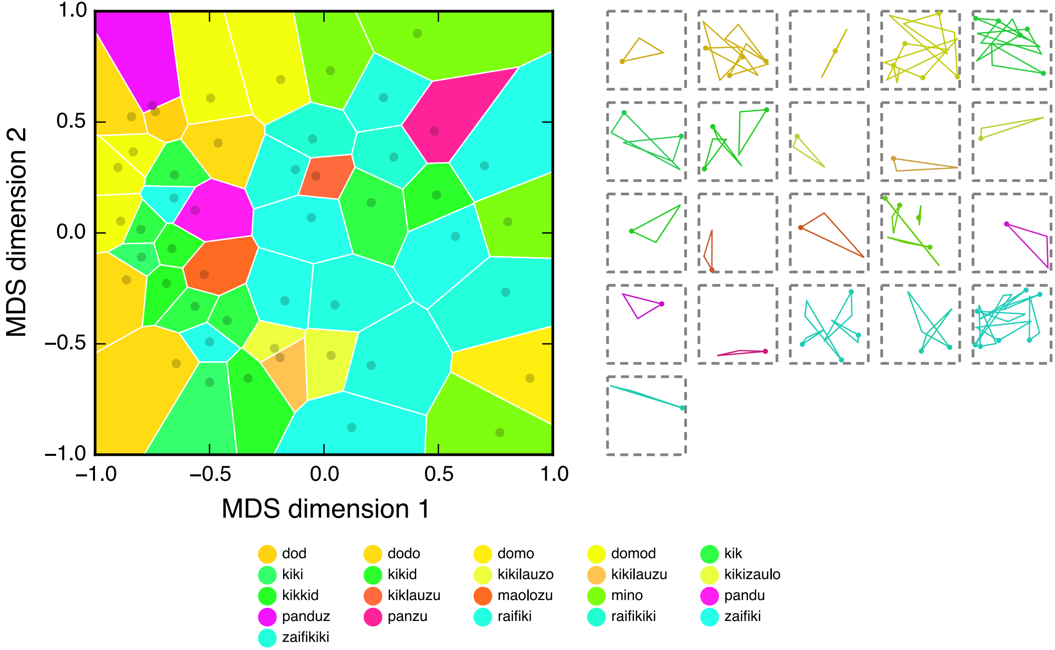

Experiment 2 Chain G Generation 10

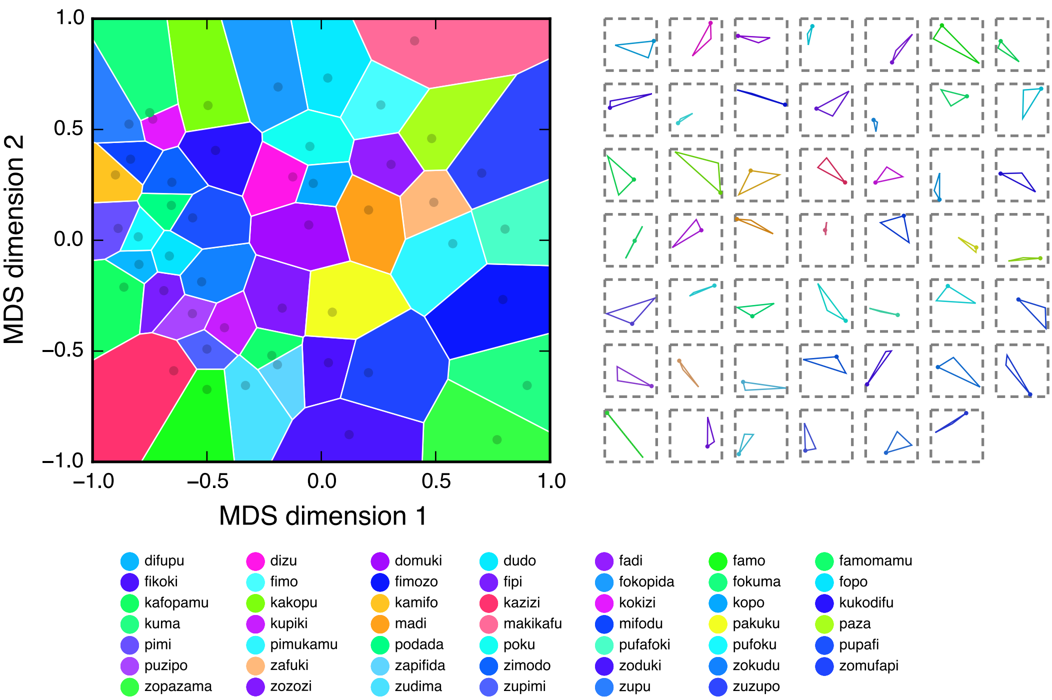

Experiment 2 Chain H Generation 0

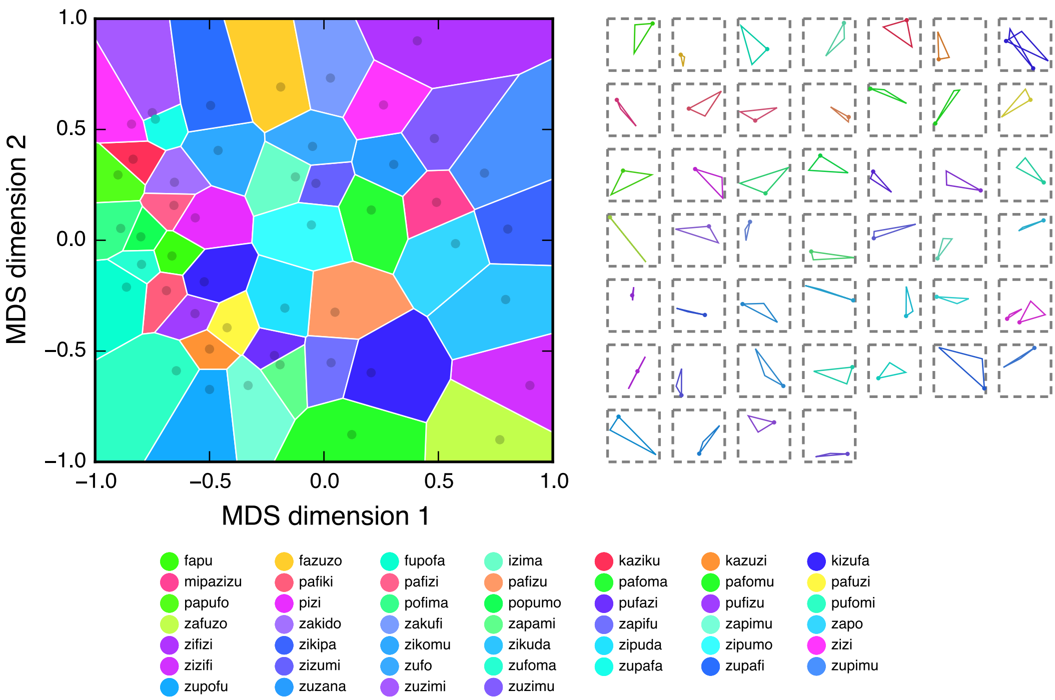

Experiment 2 Chain H Generation 1

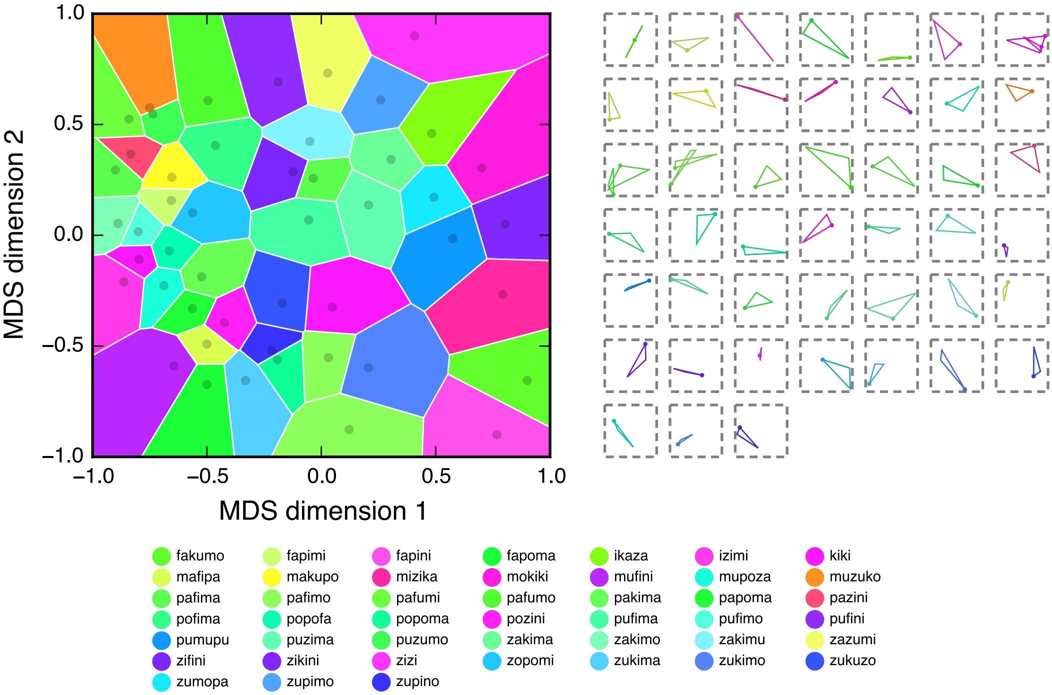

Experiment 2

Chain H

Generation 2

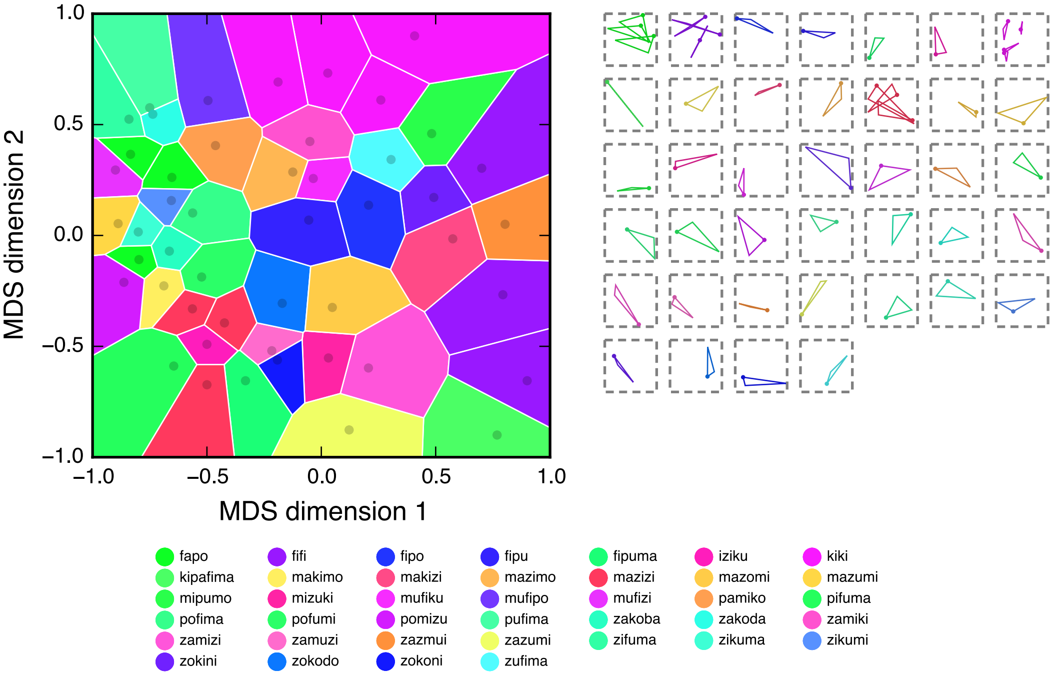

Experiment 2 Chain H Generation 3

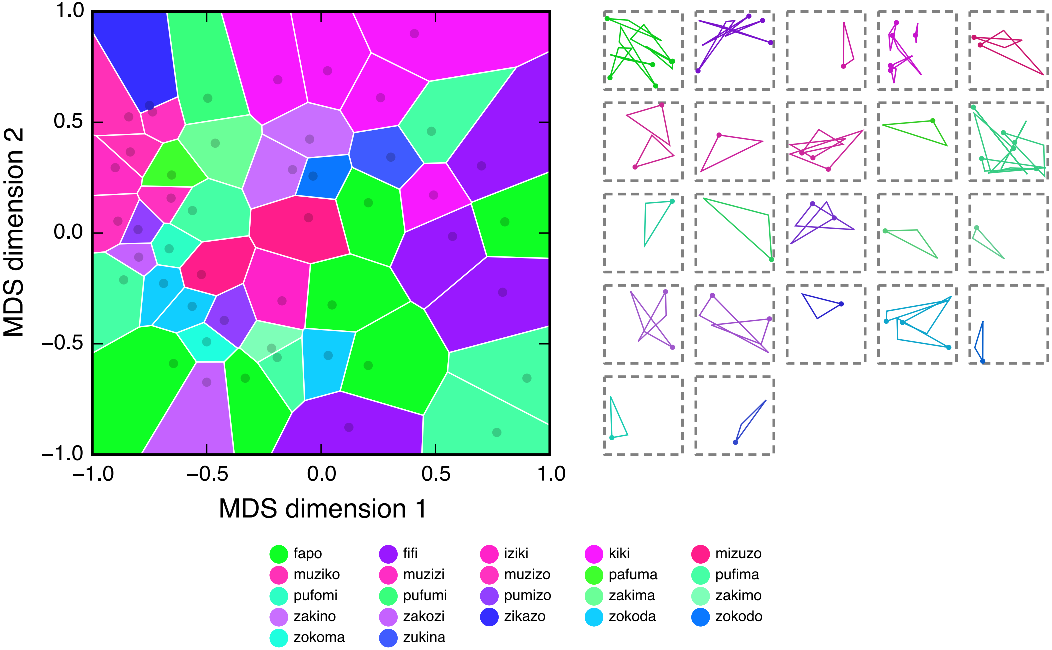

Experiment 2 Chain H Generation 4

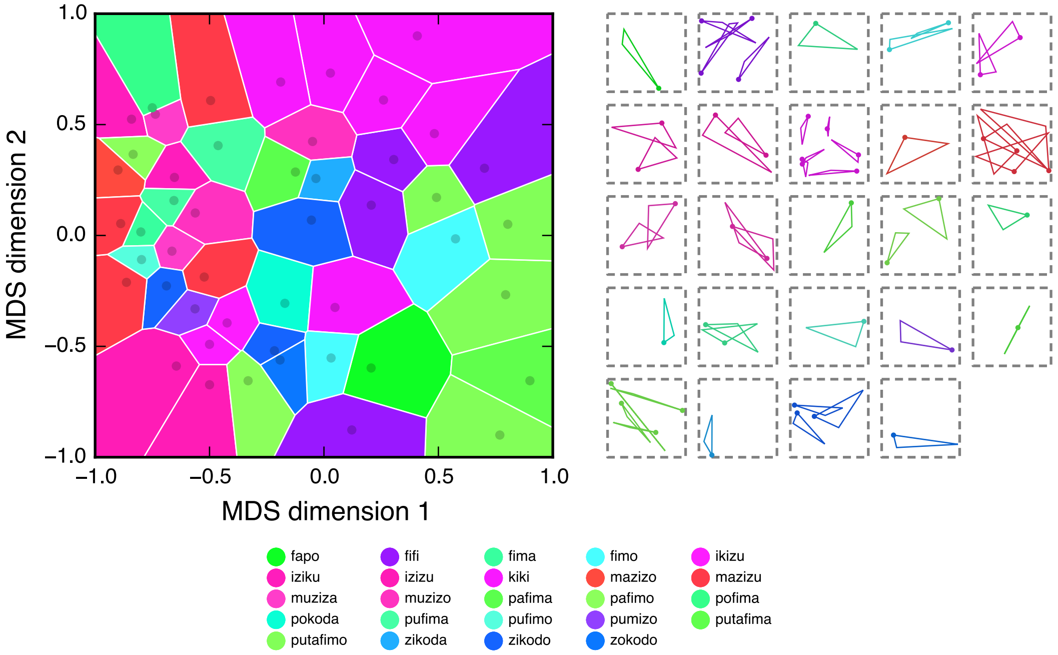

Experiment 2 Chain H Generation 5

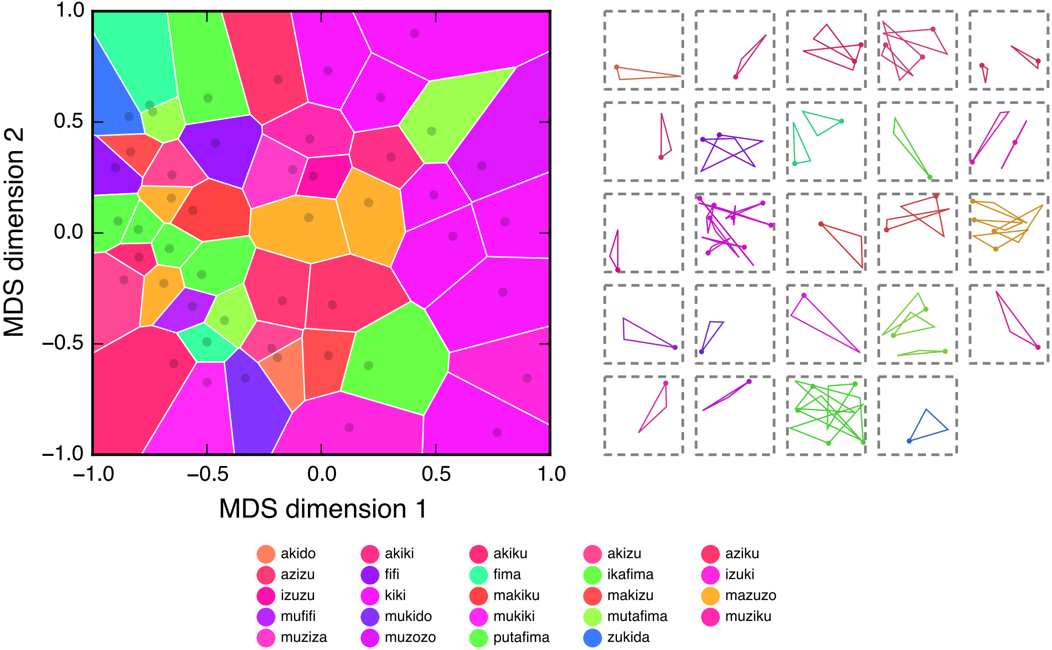

Experiment 2 Chain H Generation 6

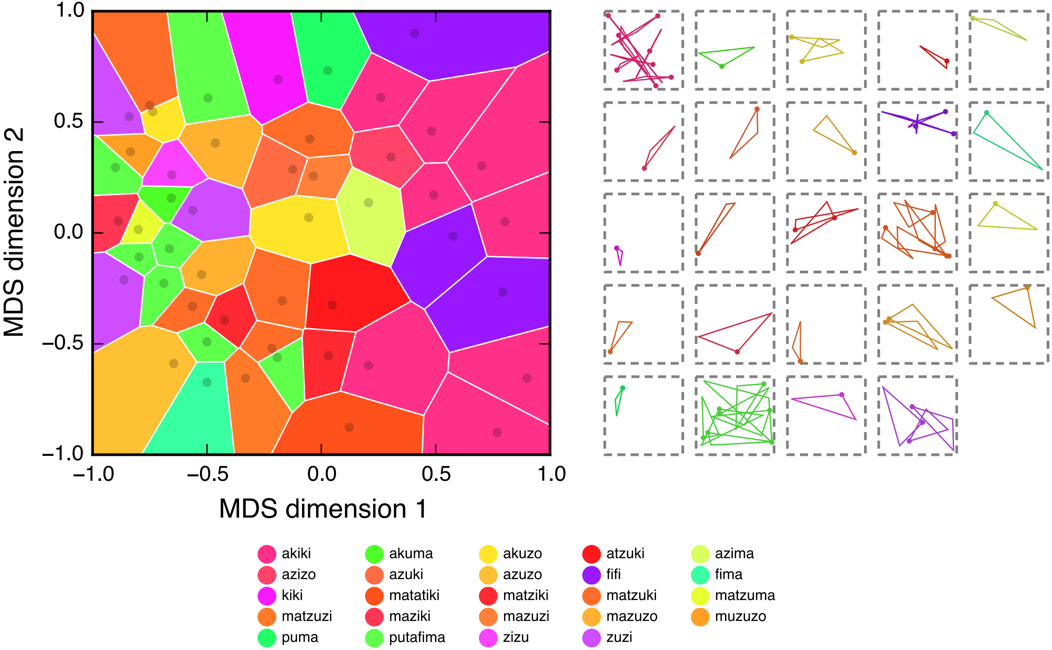

Experiment 2 | Chain H | Generation 7

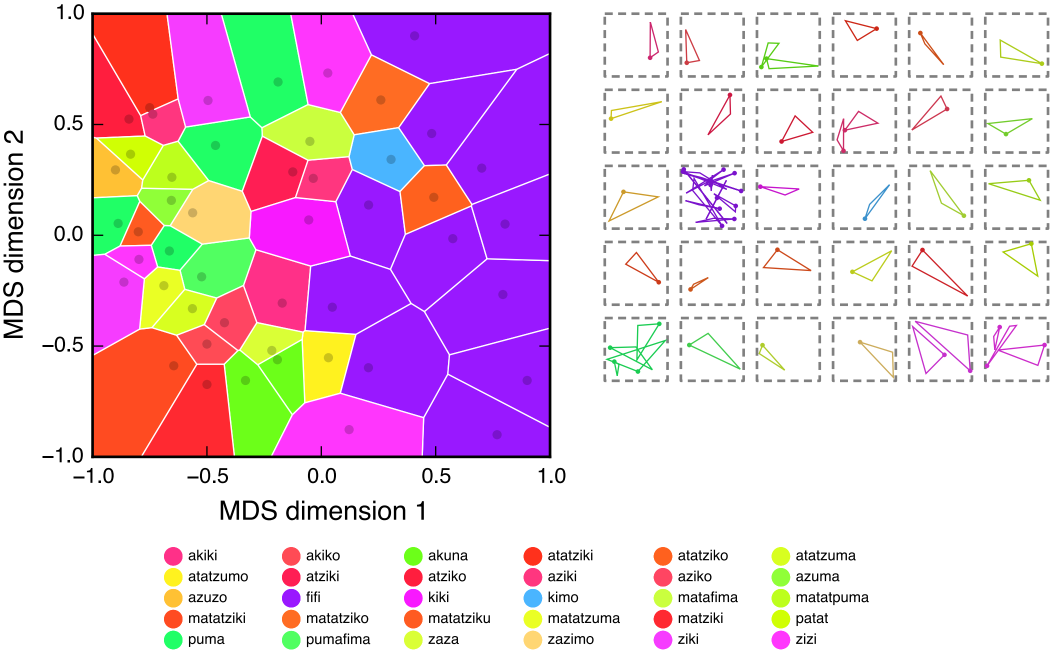

Experiment 2 Chain H Generation 8

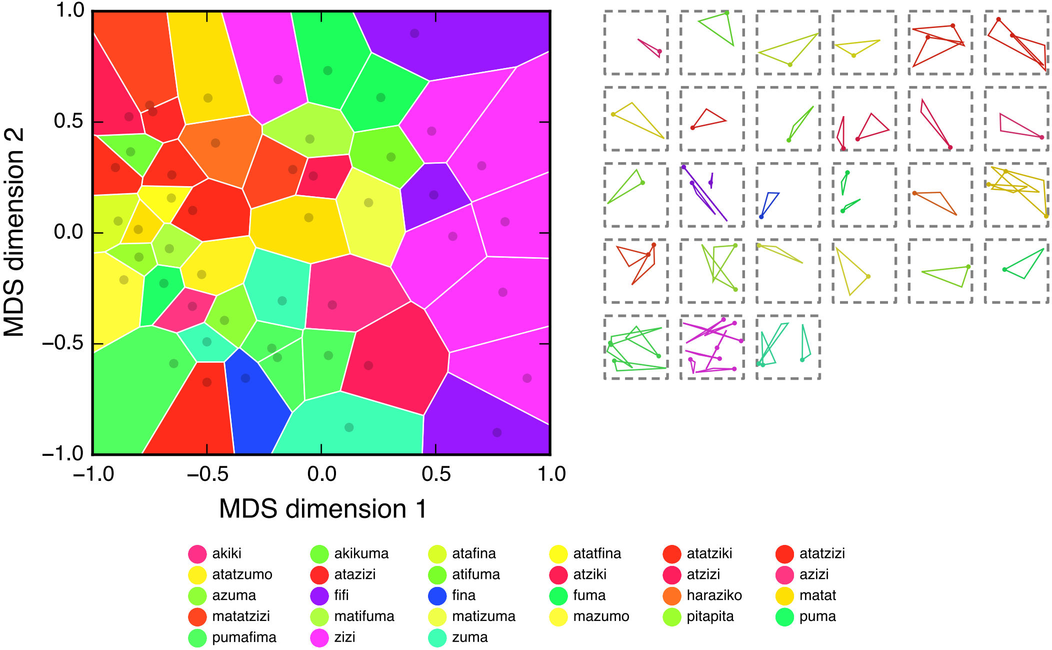

Experiment 2 Chain H Generation 9

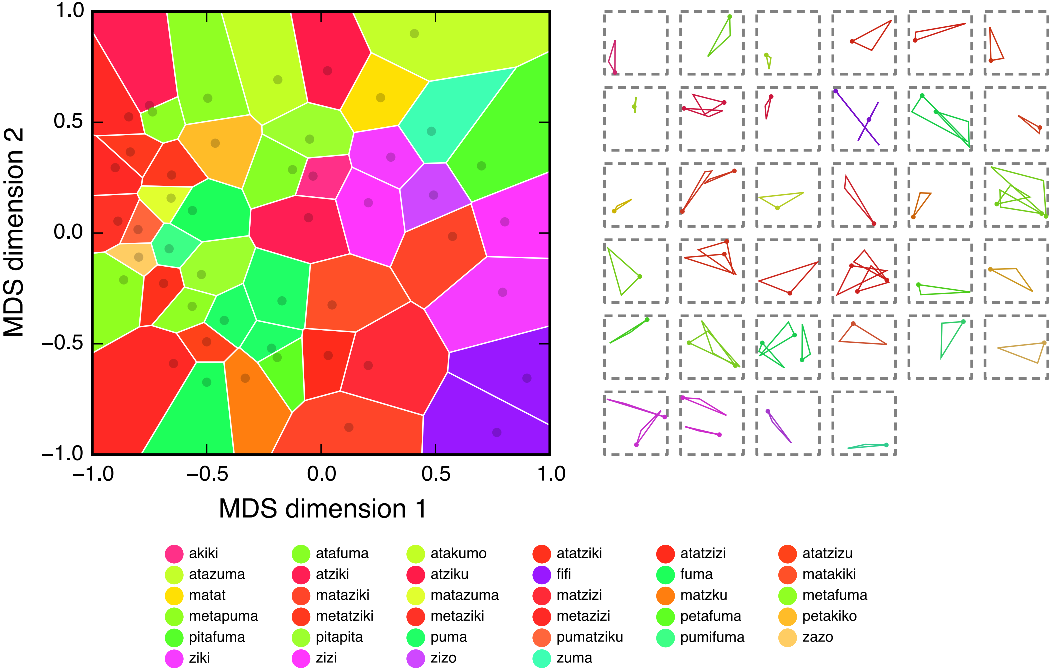

Experiment 2 Chain H Generation 10

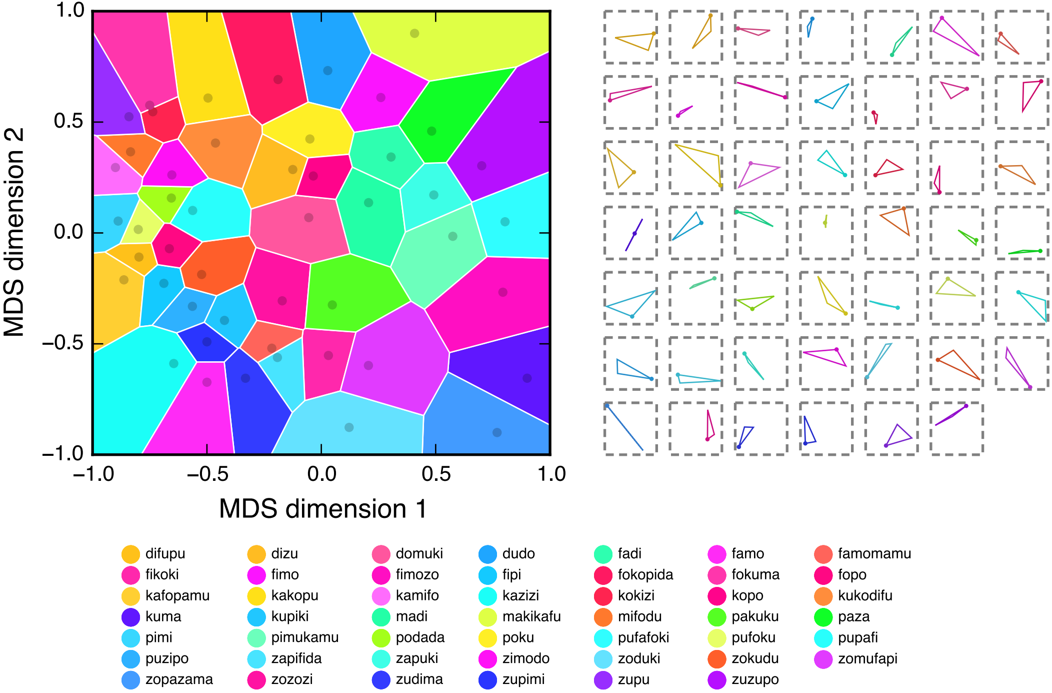

Experiment 3 Chain I Generation 0

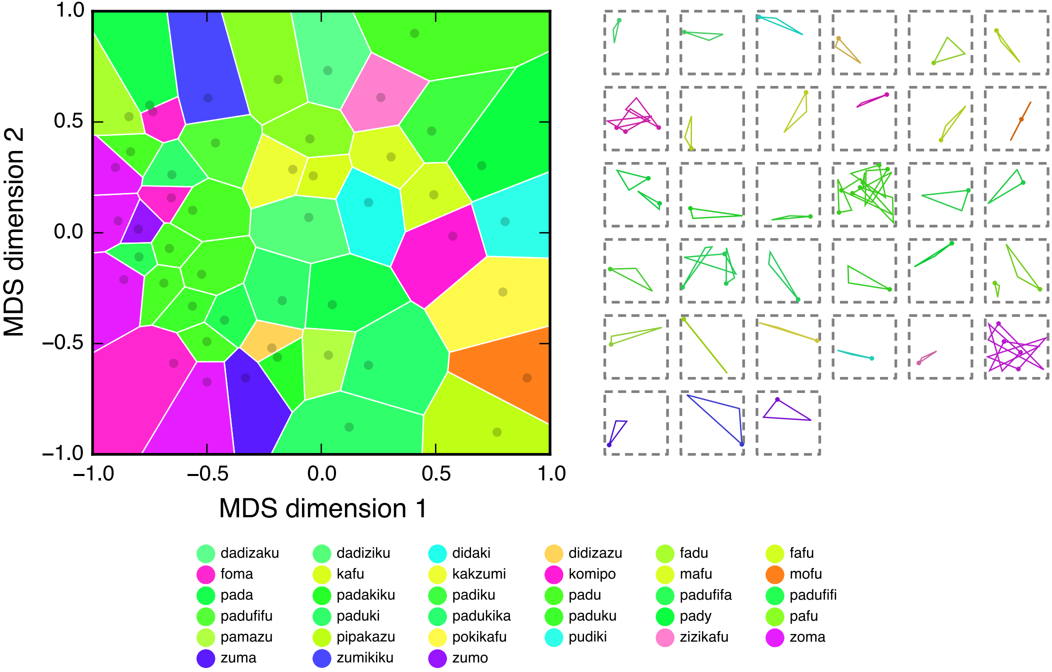

Experiment 3 Chain I Generation 1

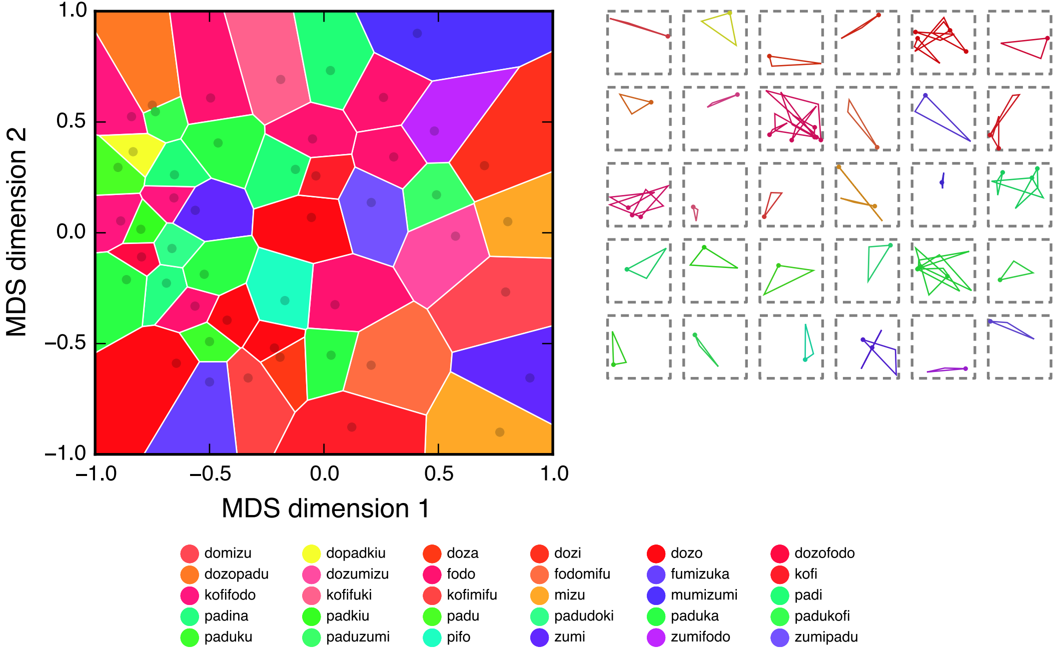

Experiment 3 Chain I Generation 2

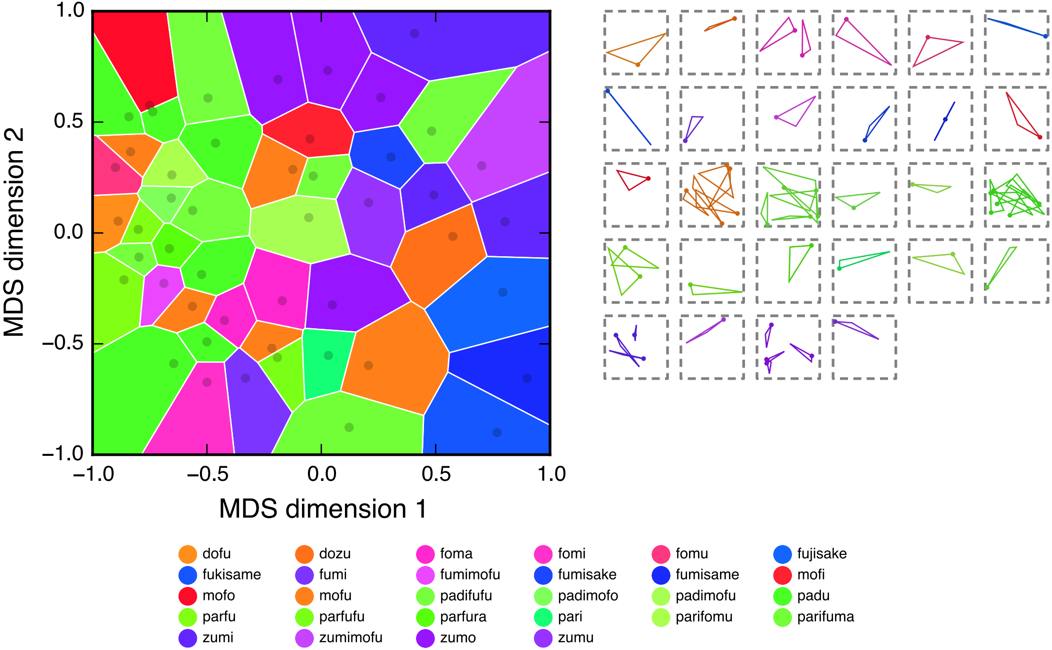

Experiment 3 Chain I Generation 3

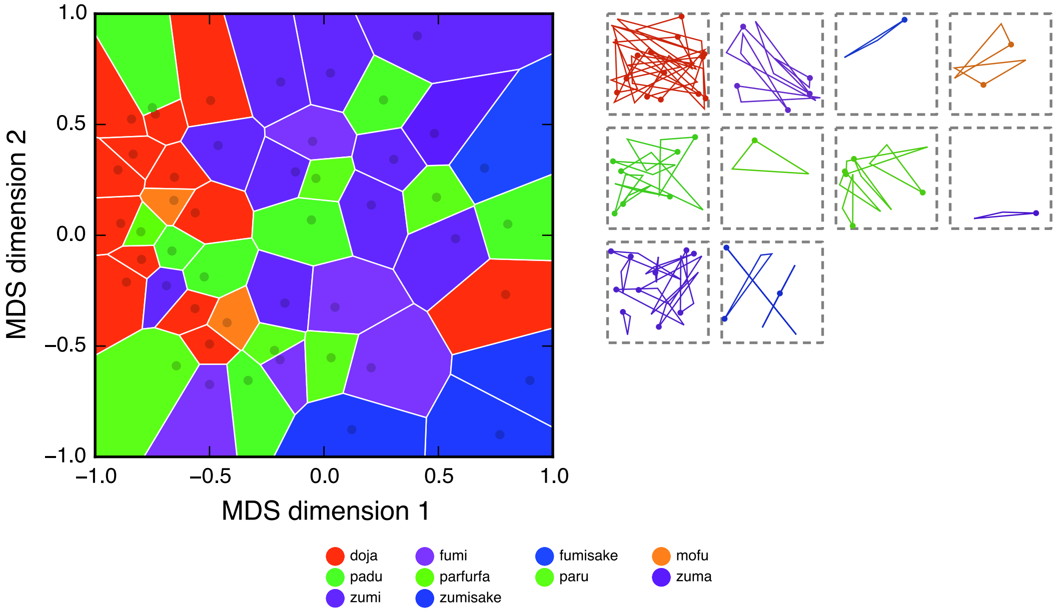

Experiment 3 Chain I Generation 4

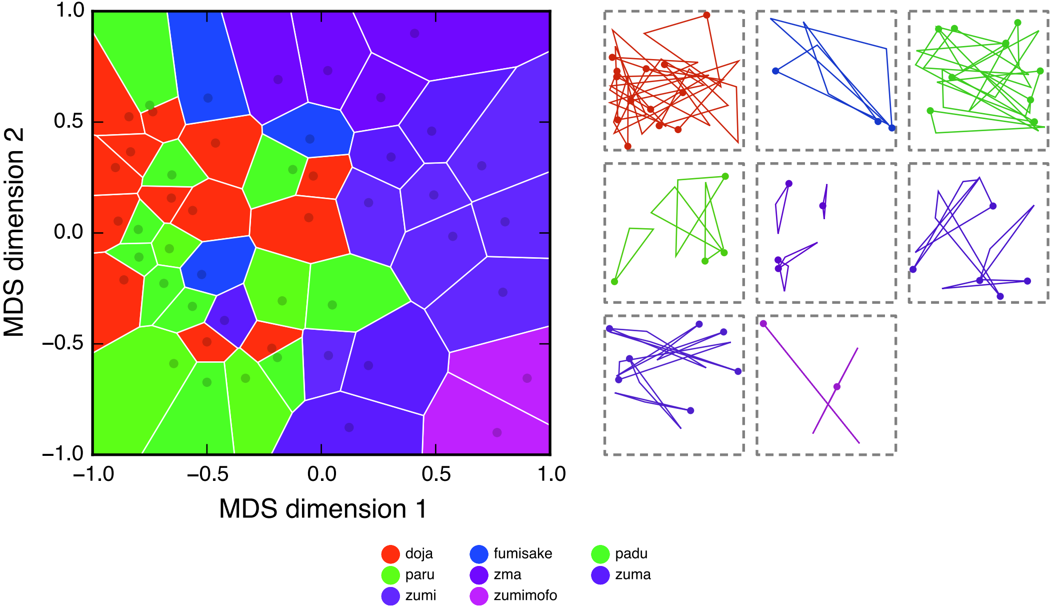

Experiment 3 Chain I Generation 5

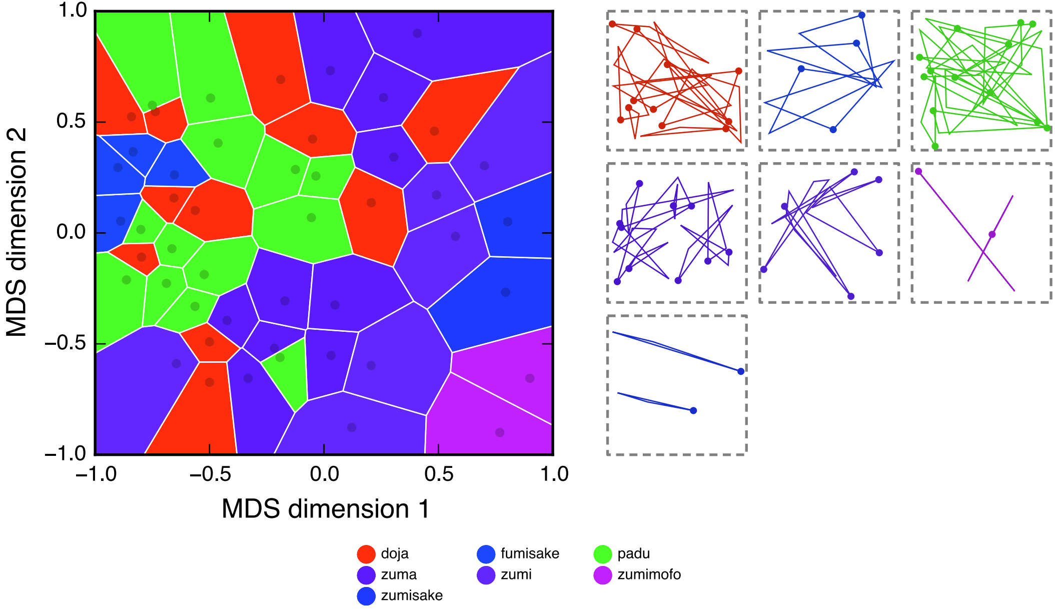

Experiment 3 Chain I Generation 6

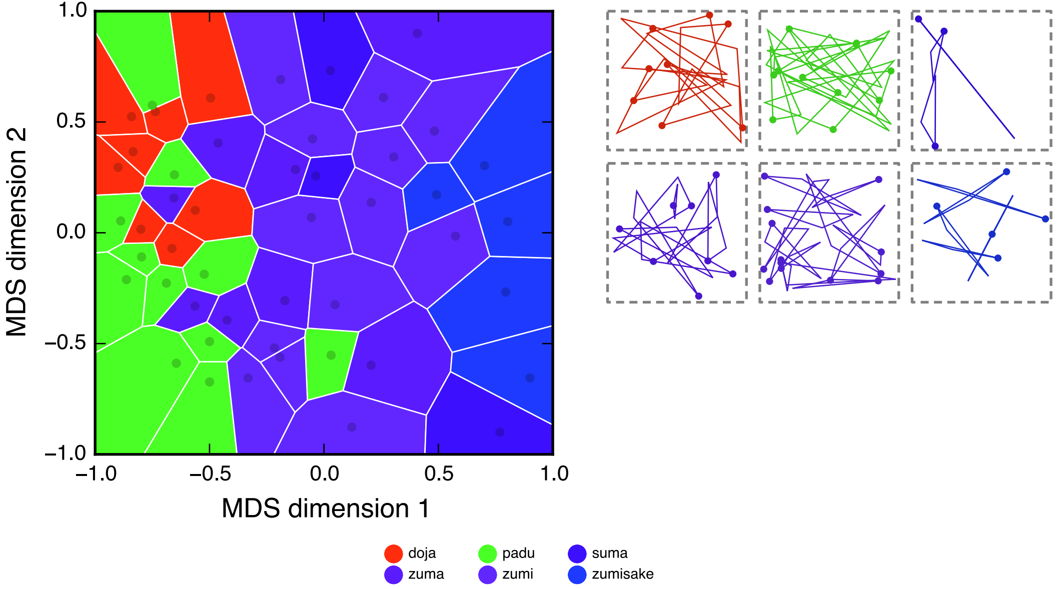

Experiment 3 Chain I Generation 7

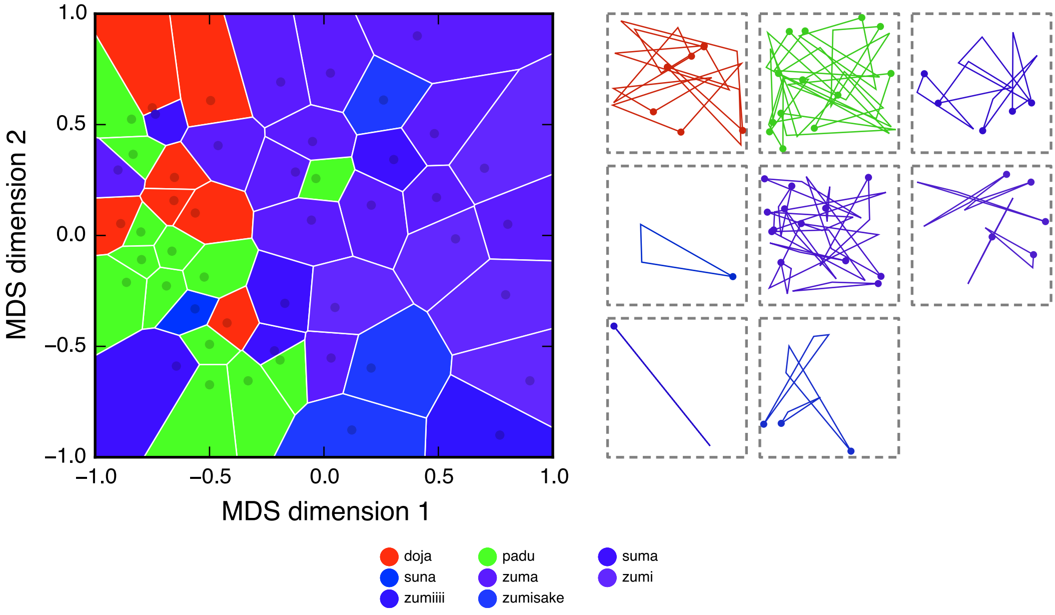

Experiment 3 Chain I Generation 8

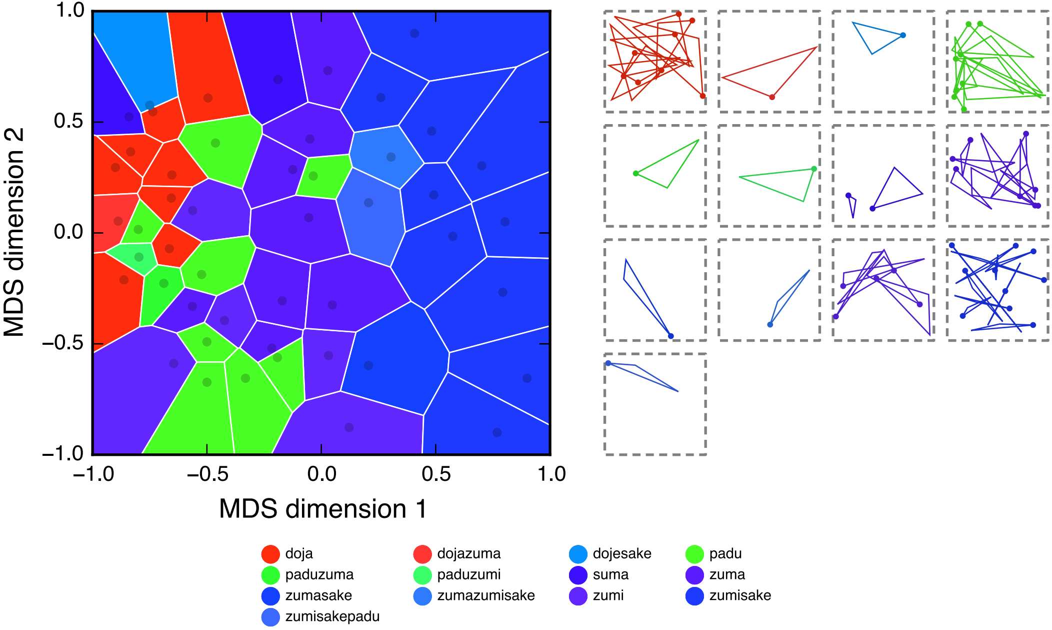

Experiment 3 Chain I Generation 9

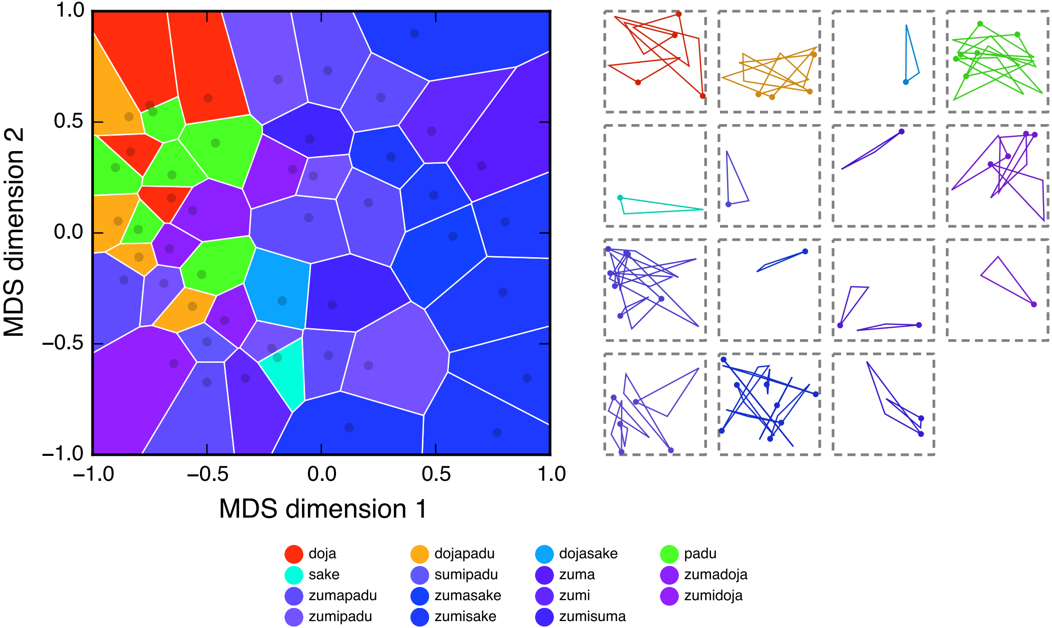

Experiment 3 Chain I Generation 10

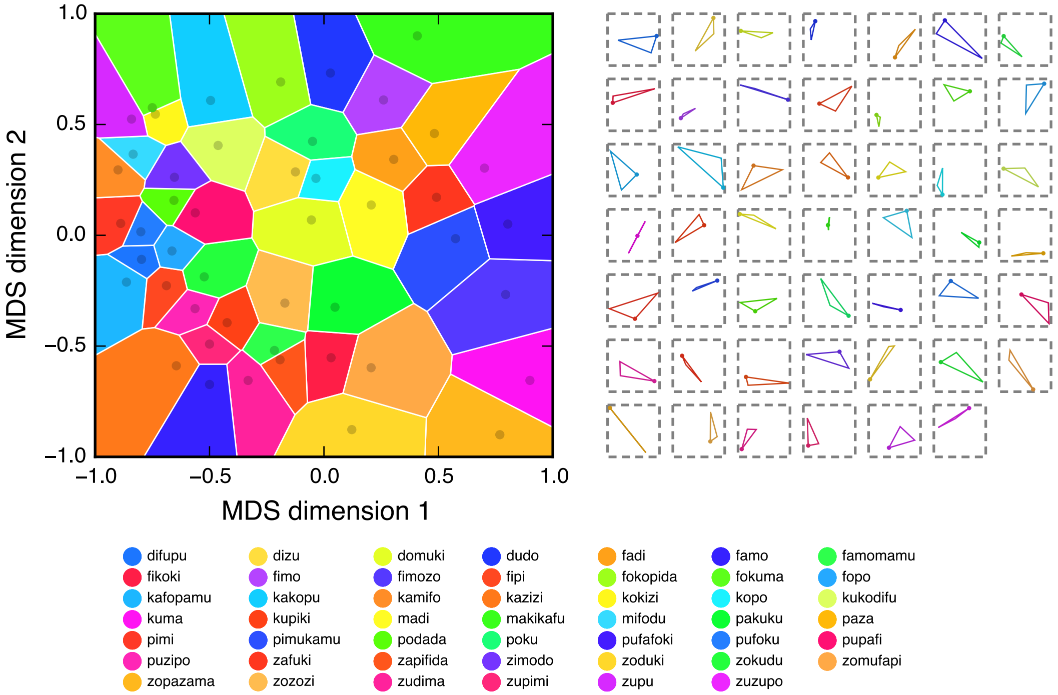

Experiment 3 Chain J Generation 0

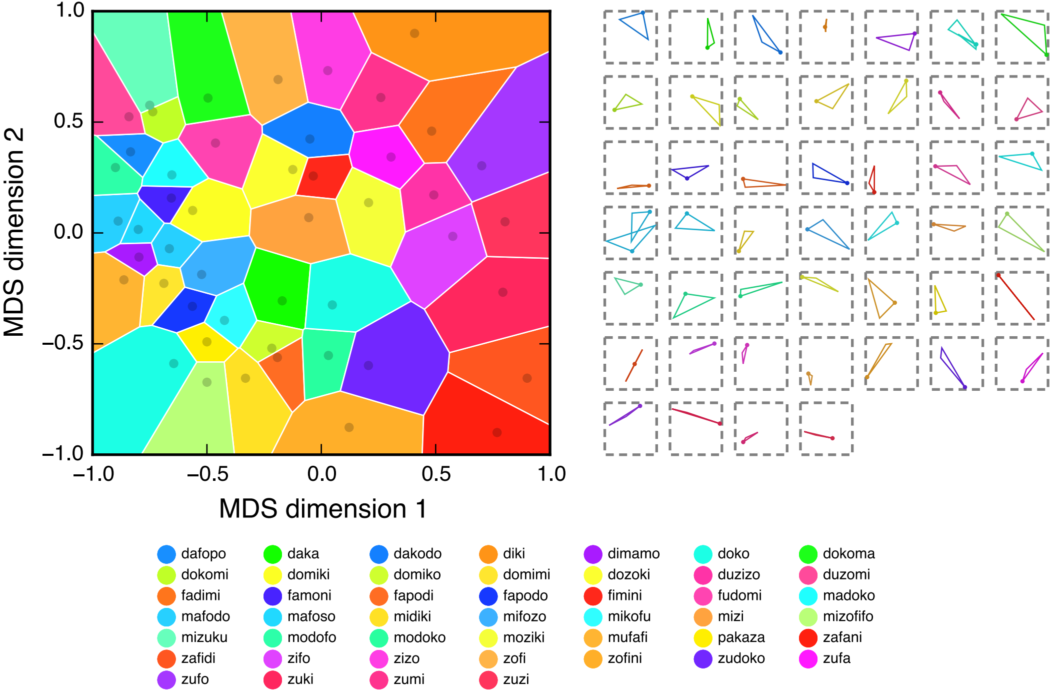

Experiment 3 Chain J Generation 1

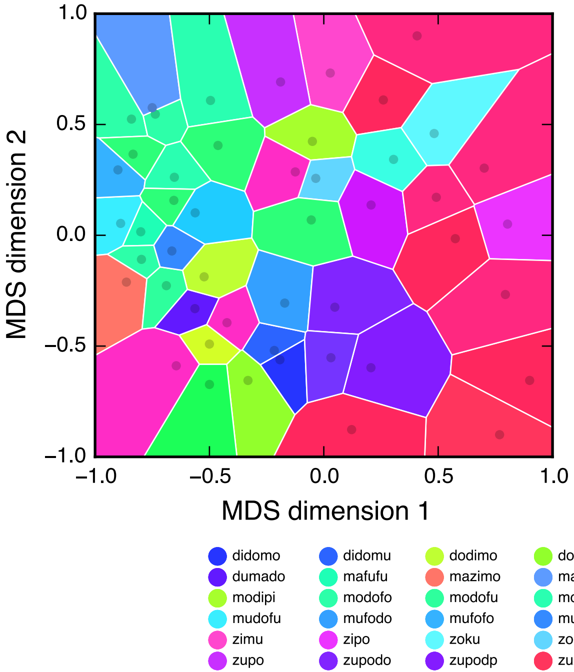

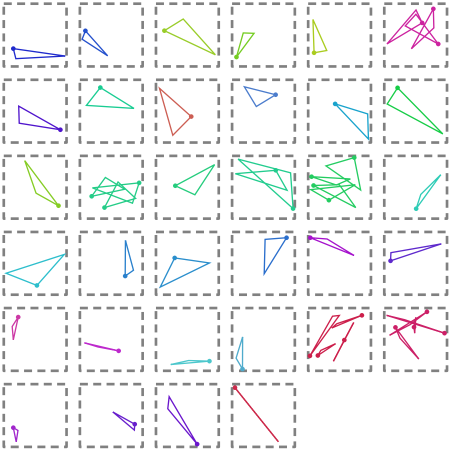

Experiment 3 Chain J Generation 2

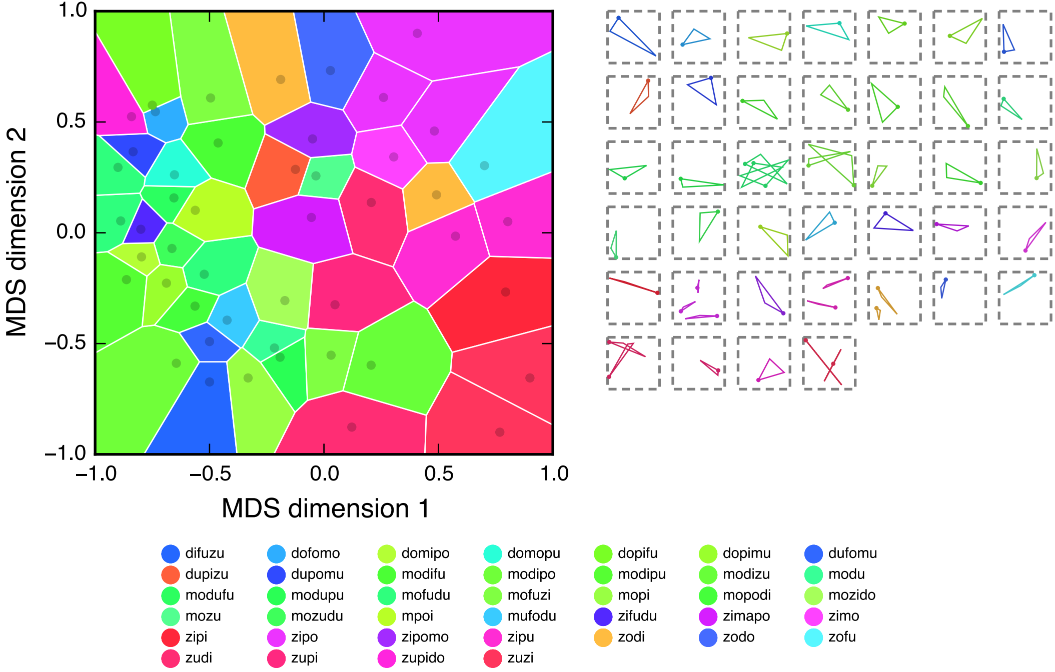

Experiment 3 Chain J Generation 3

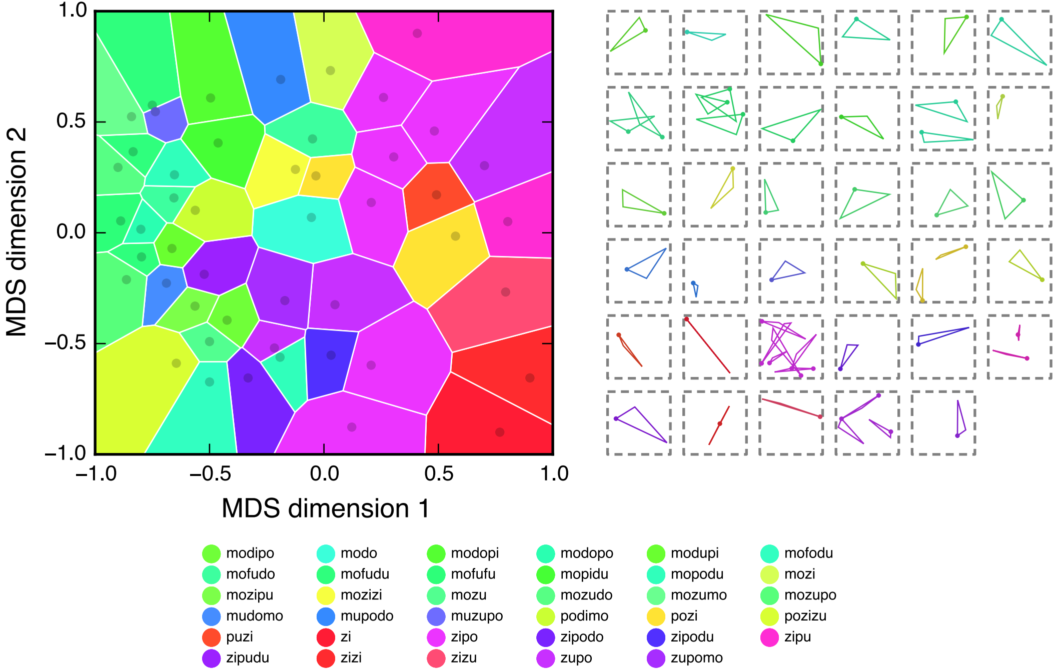

Experiment 3 Chain J Generation 4

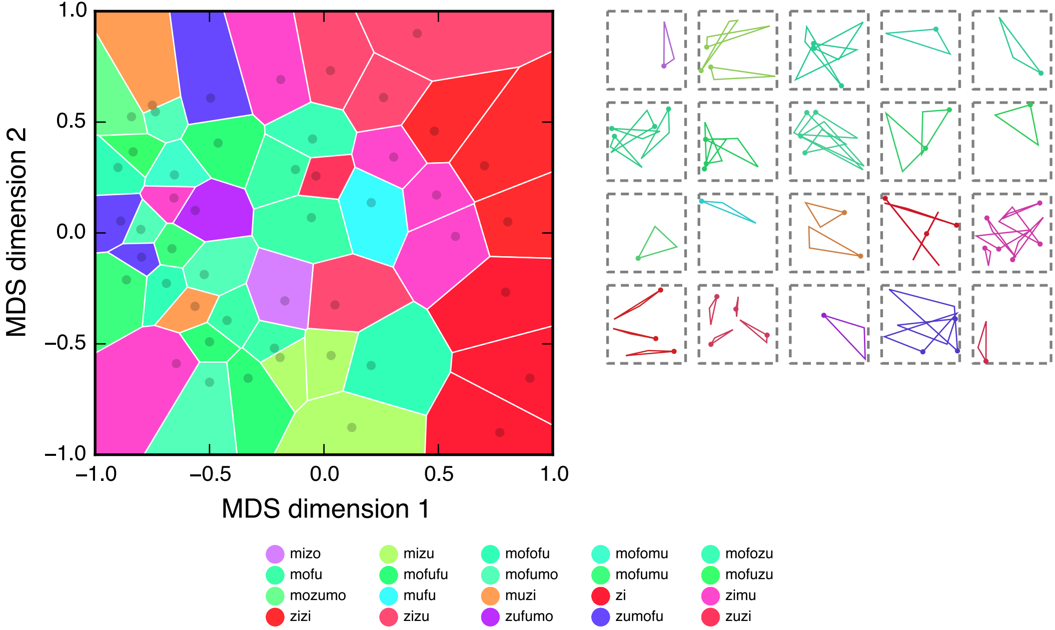

Experiment 3 Chain J Generation 5

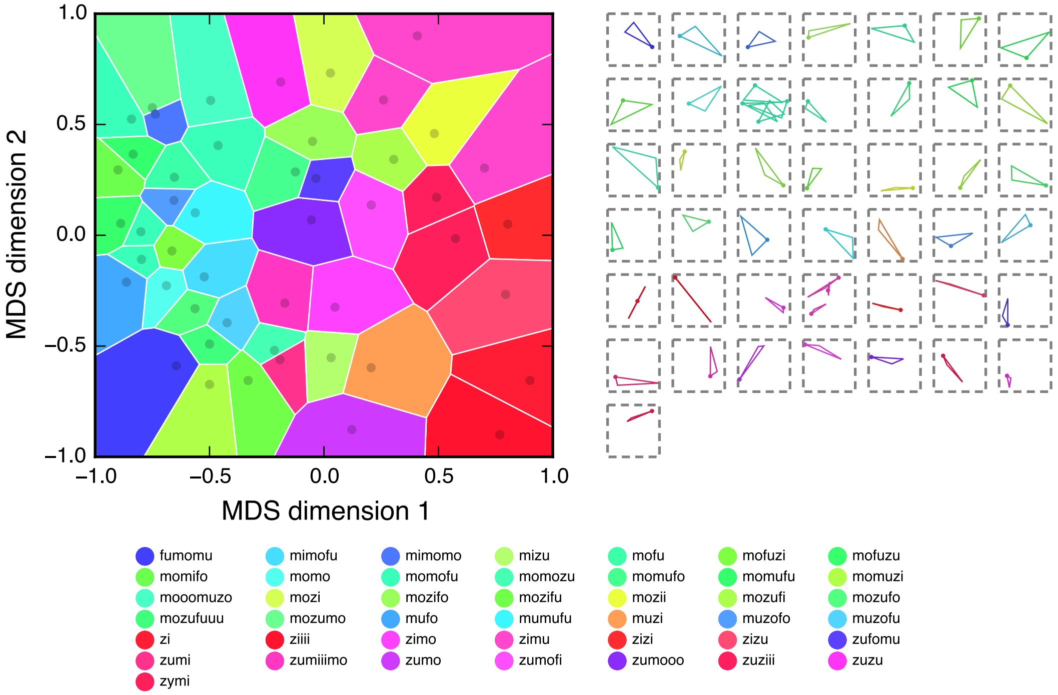

Experiment 3 Chain J Generation 6

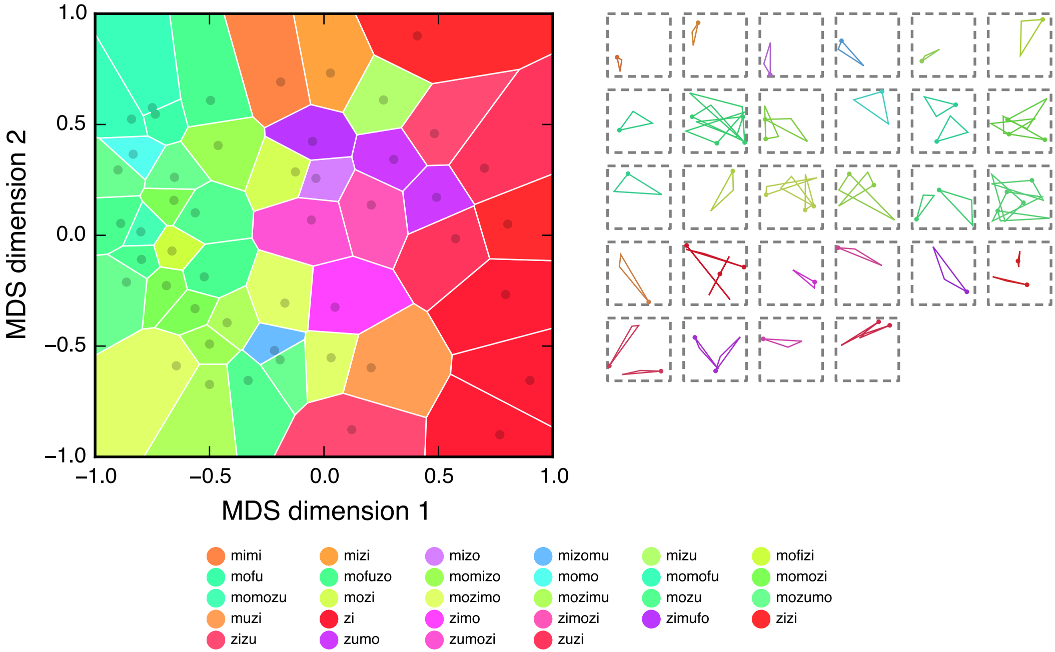

Experiment 3 Chain J Generation 7

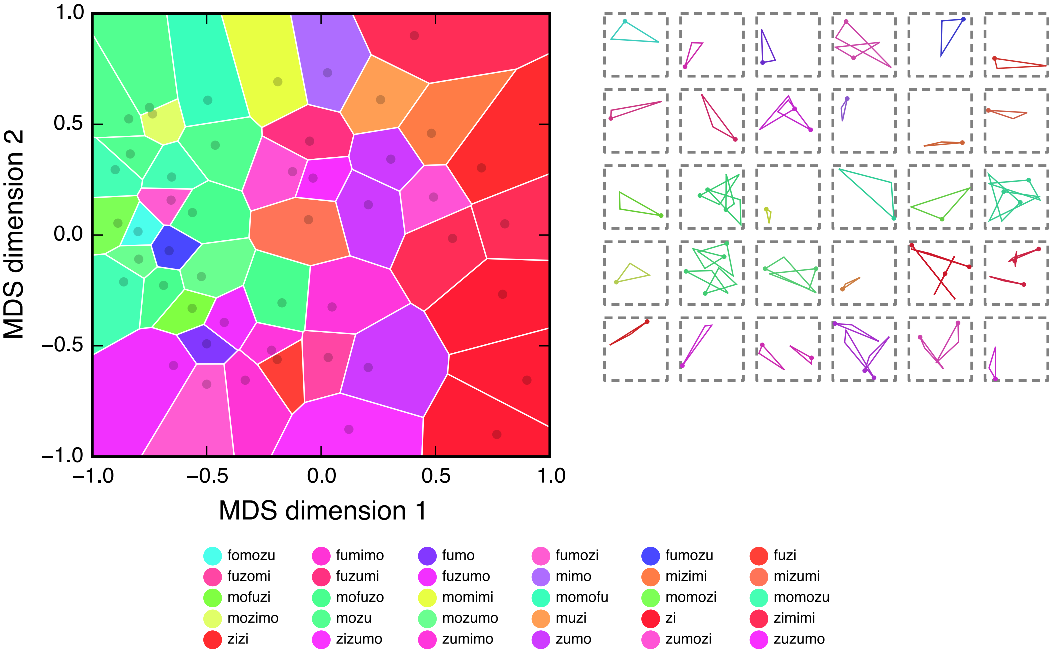

Experiment 3 Chain J Generation 8

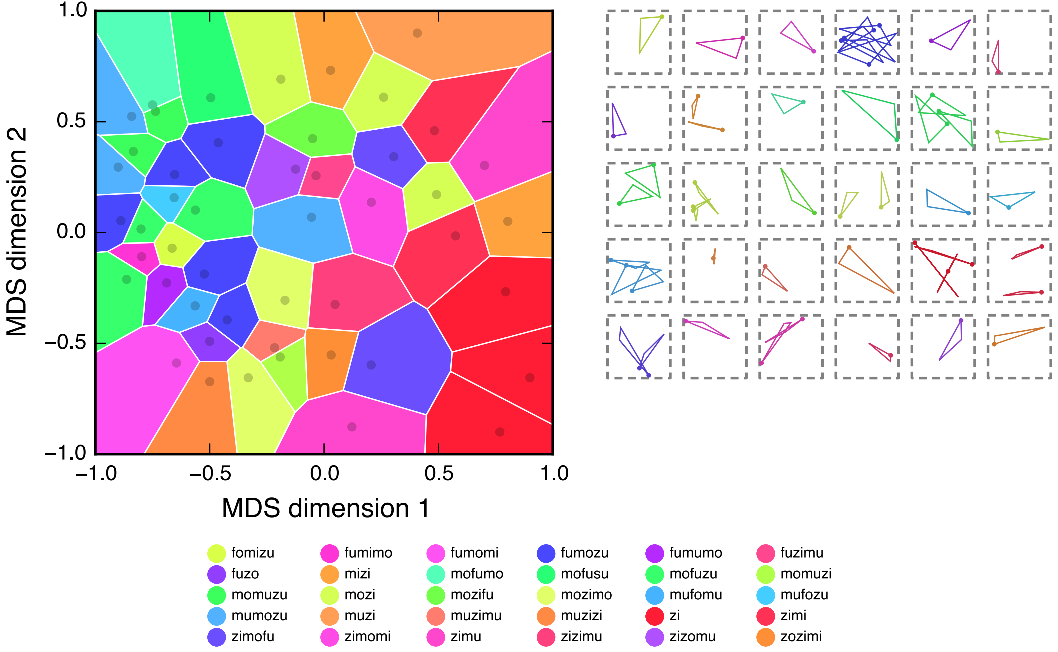

Experiment 3 Chain J Generation 9

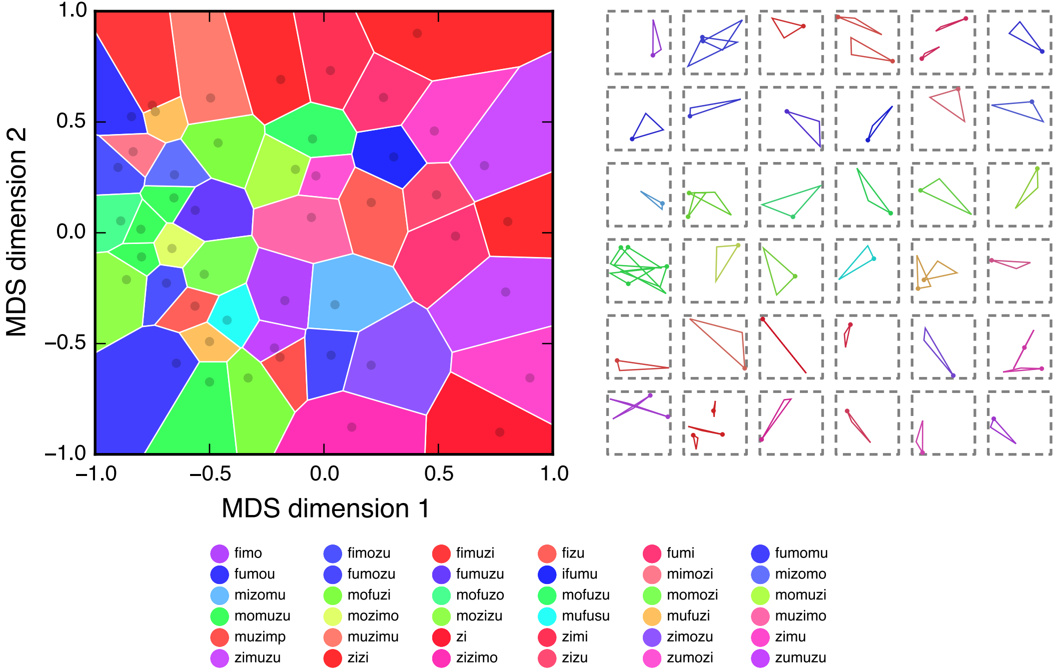

Experiment 3 Chain J Generation 10

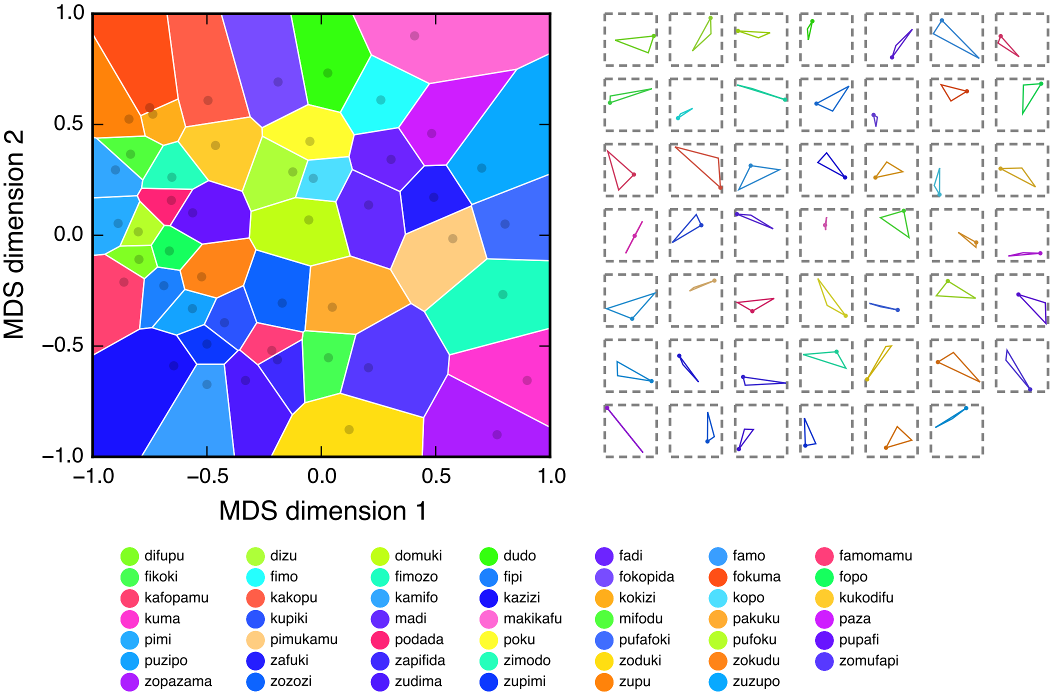

Experiment 3 Chain K Generation 0

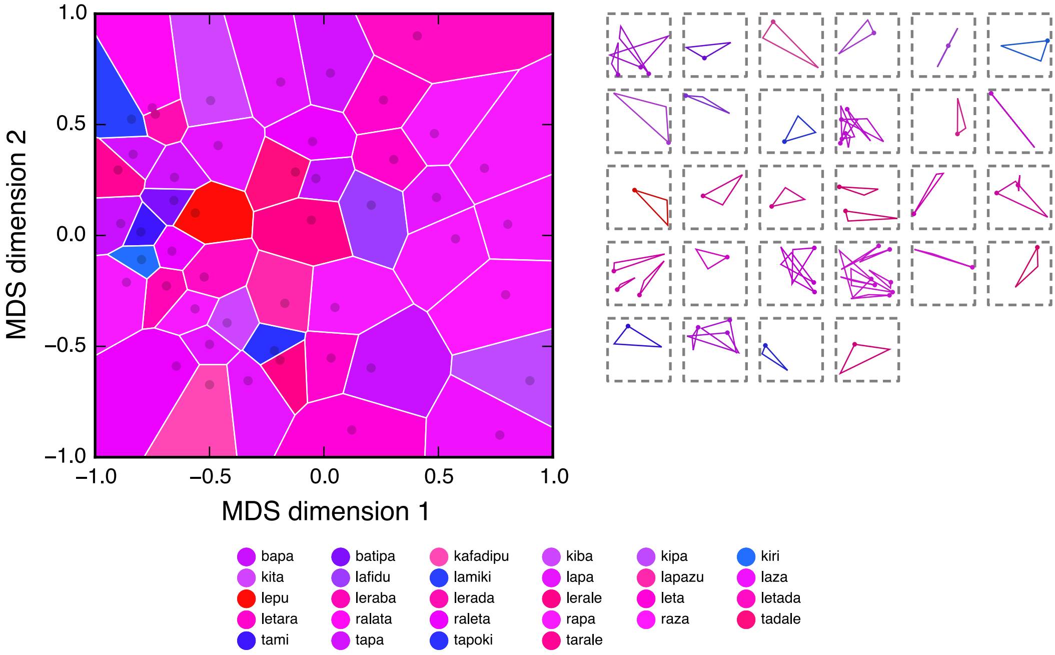

Experiment 3 Chain K Generation 1

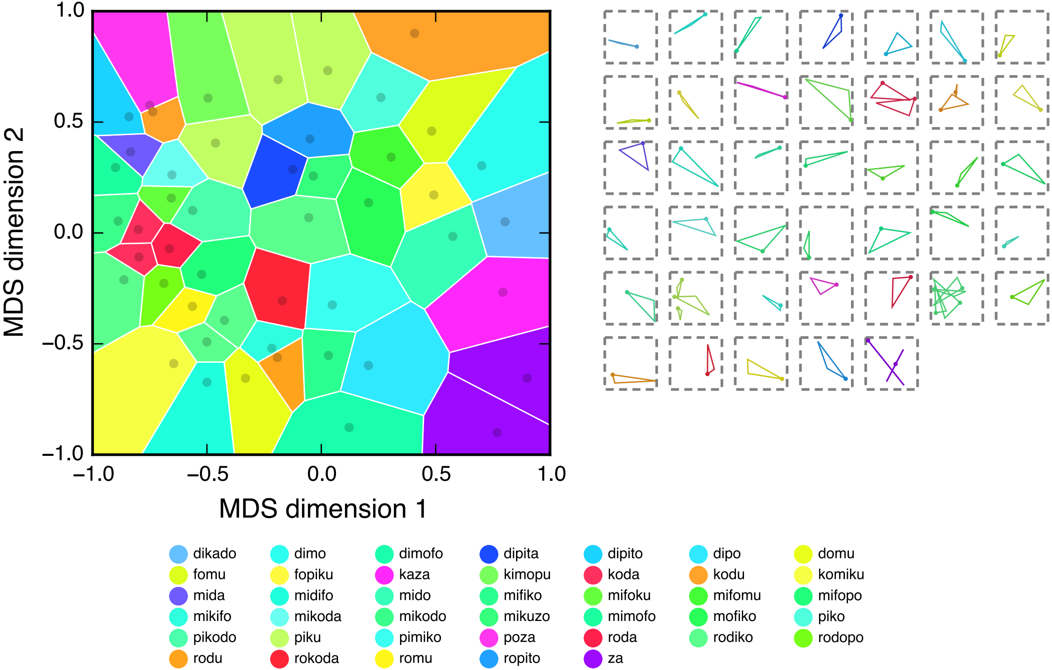

Experiment 3 Chain K Generation 2

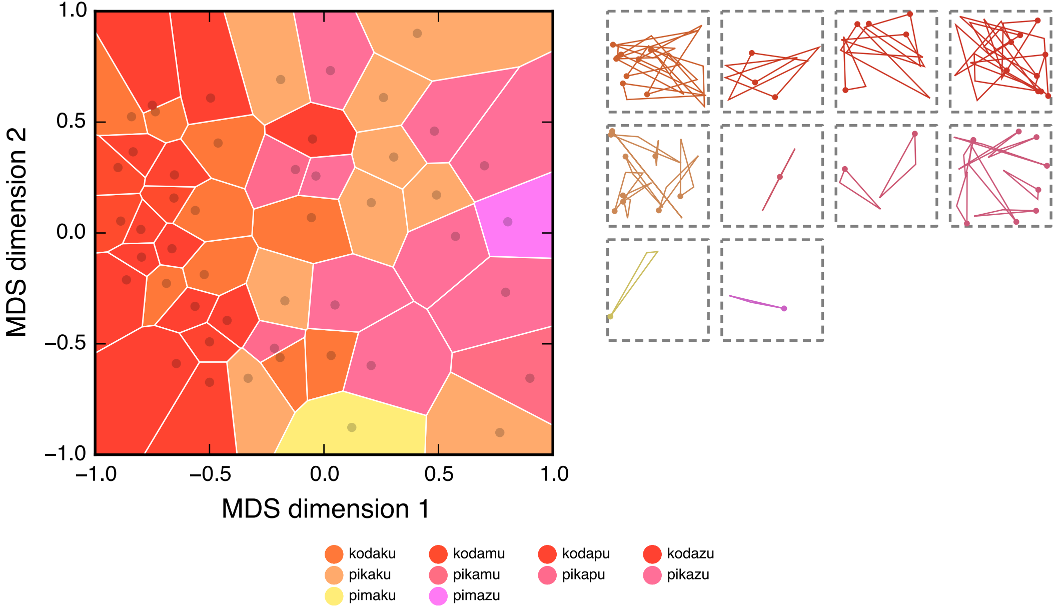

Experiment 3 Chain K Generation 3

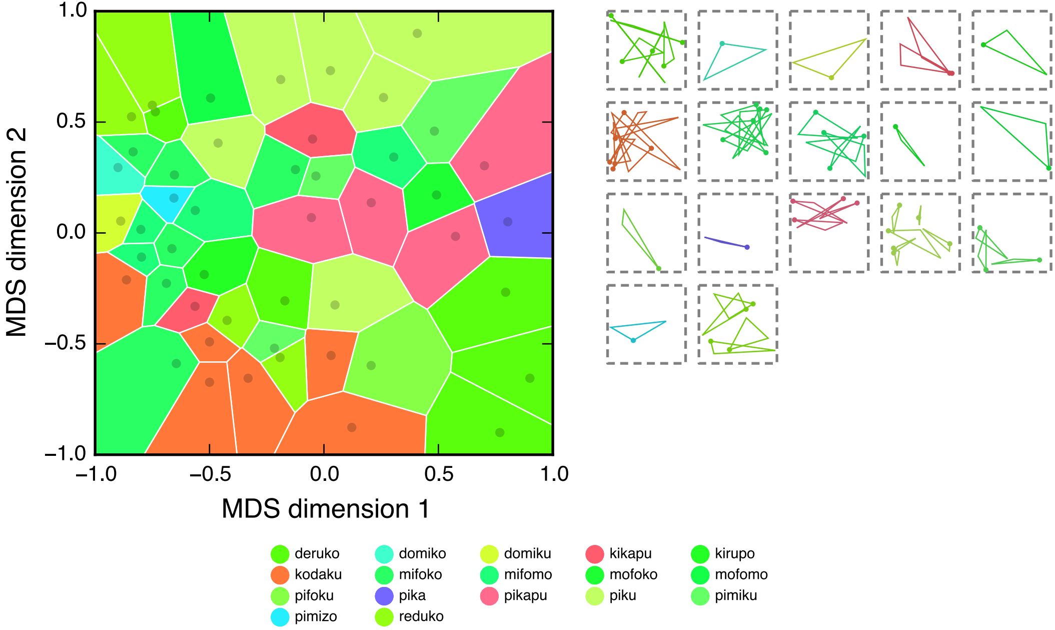

Experiment 3 Chain K Generation 4

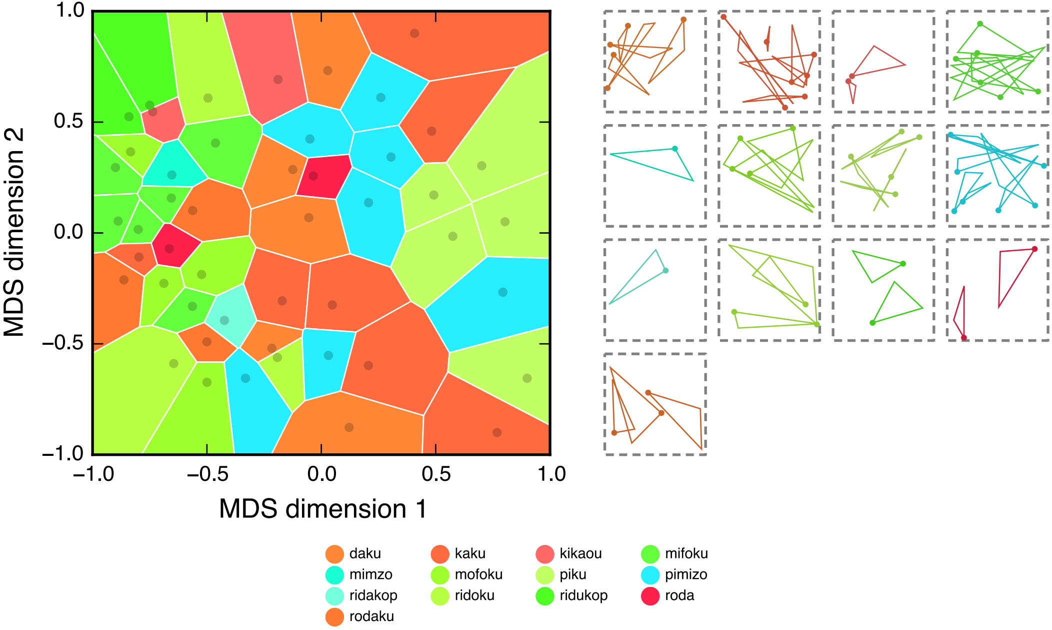

Experiment 3 Chain K Generation 5

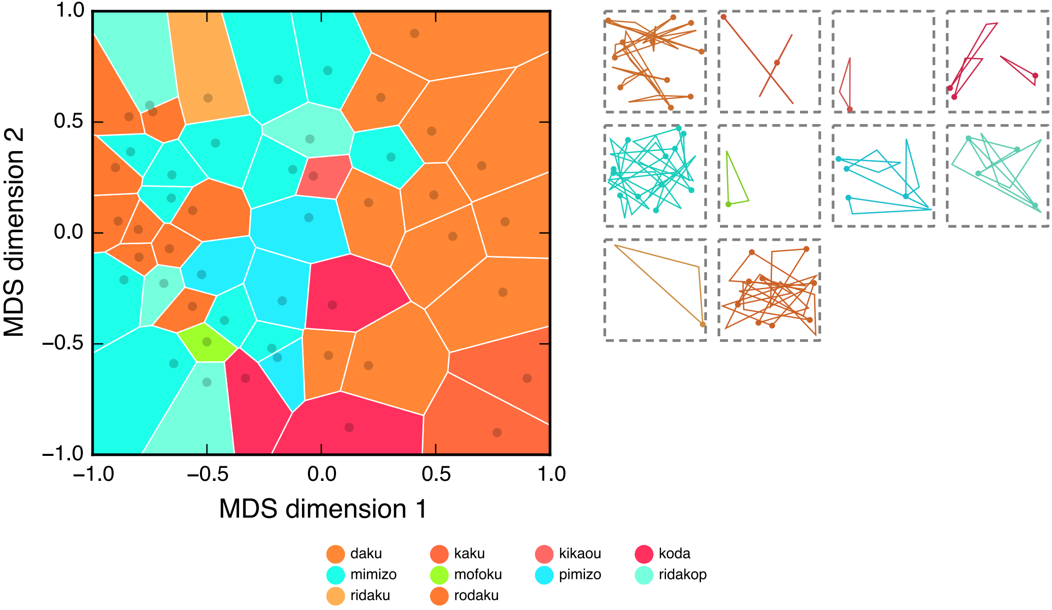

Experiment 3 Chain K Generation 6

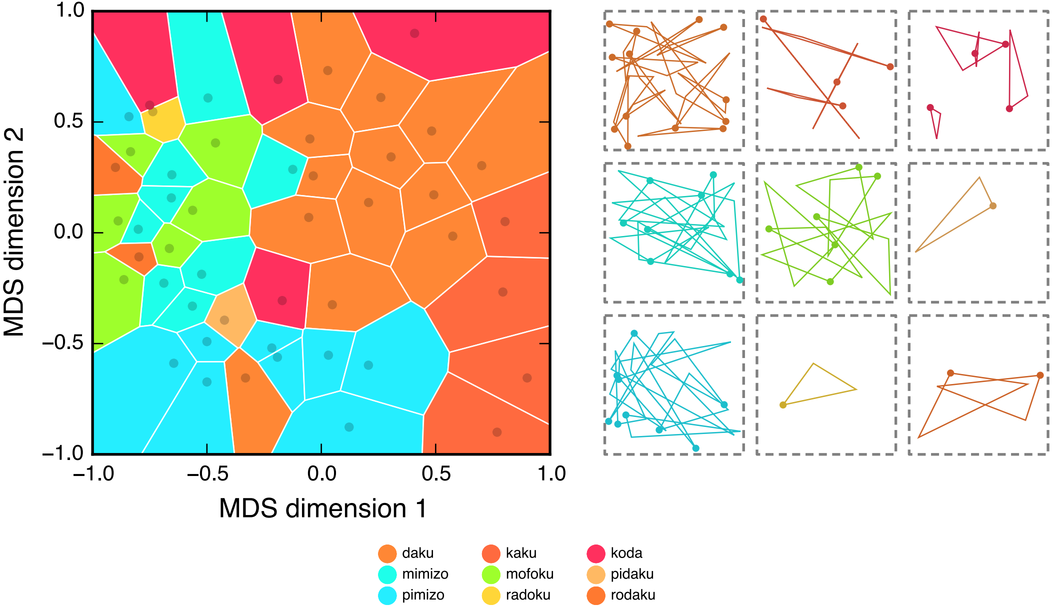

Experiment 3 Chain K Generation 7

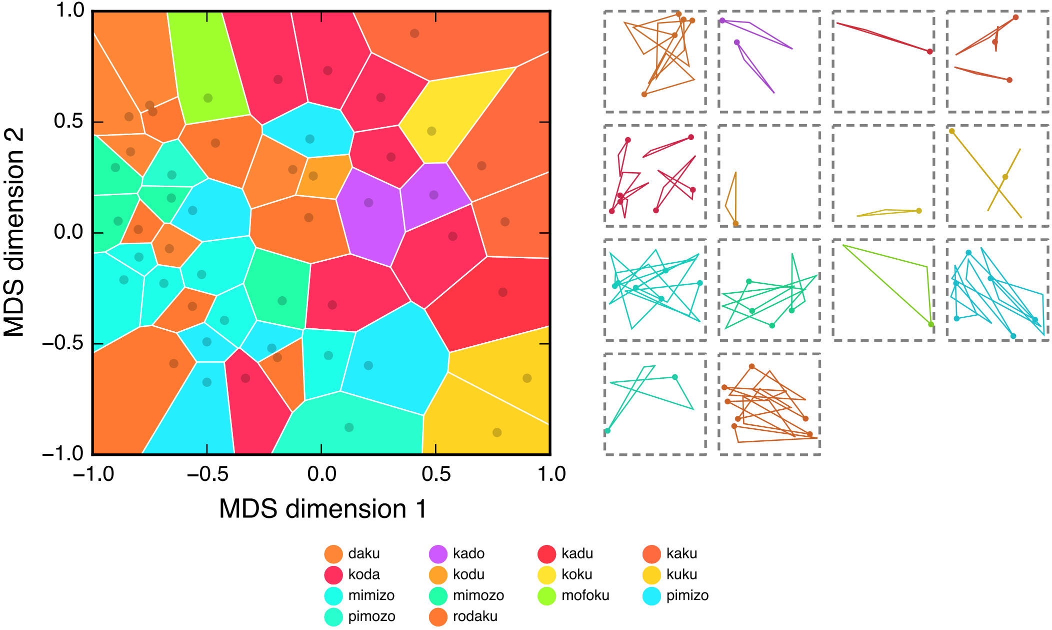

Experiment 3 Chain K Generation 8

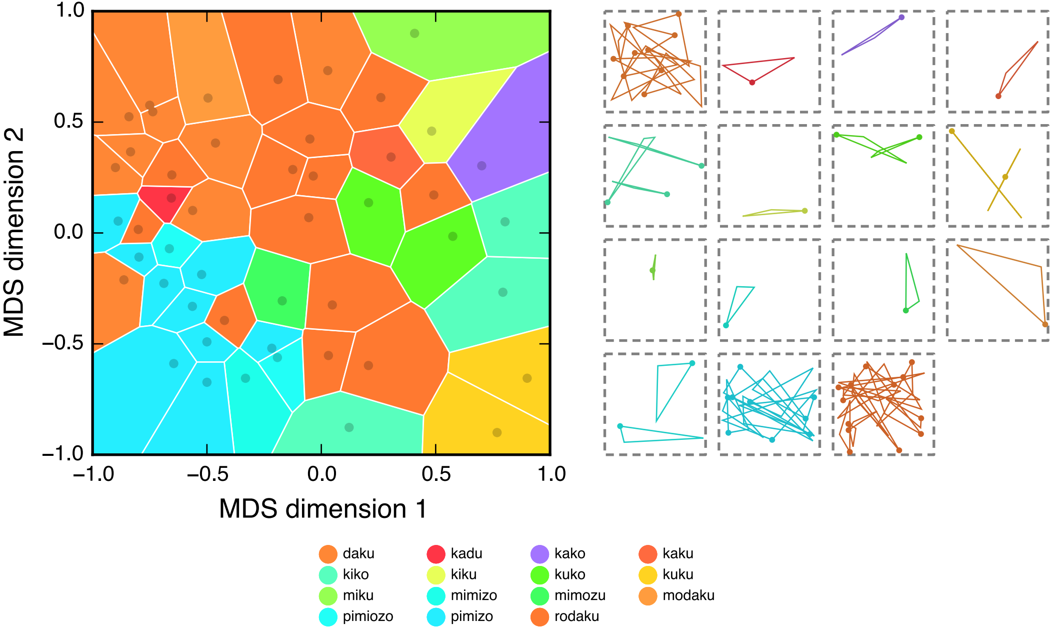

Experiment 3 Chain K Generation 9

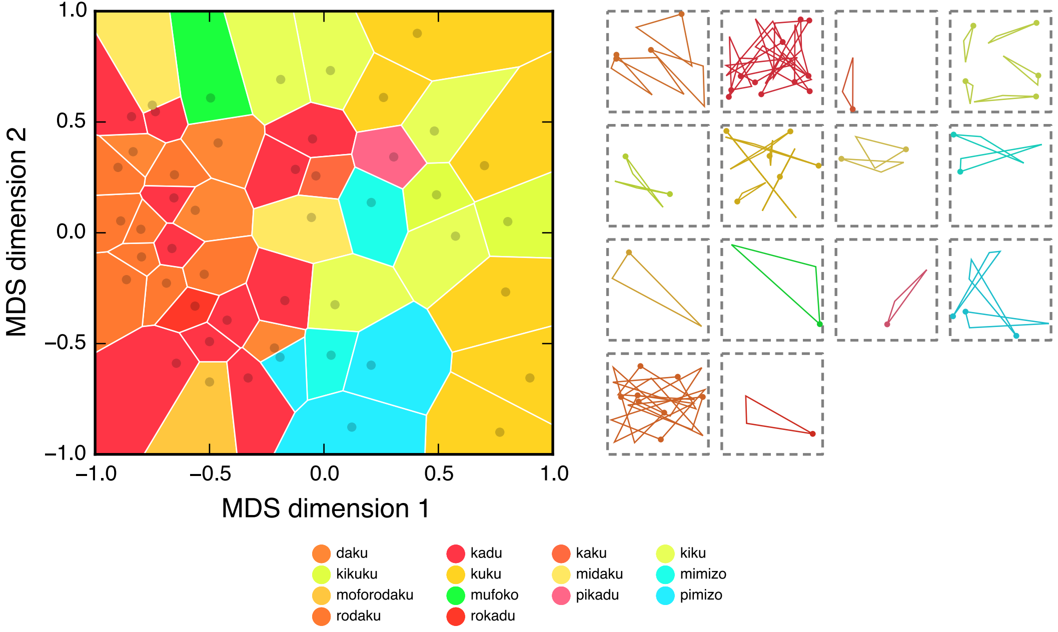

Experiment 3 Chain K Generation 10

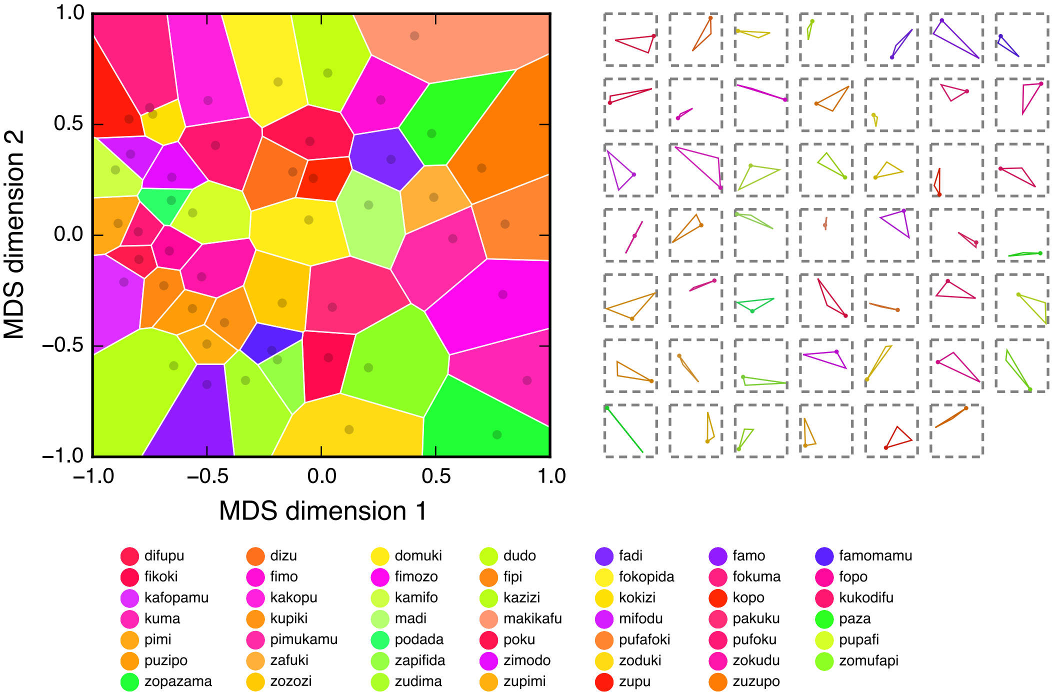

Experiment 3 Chain L Generation 0

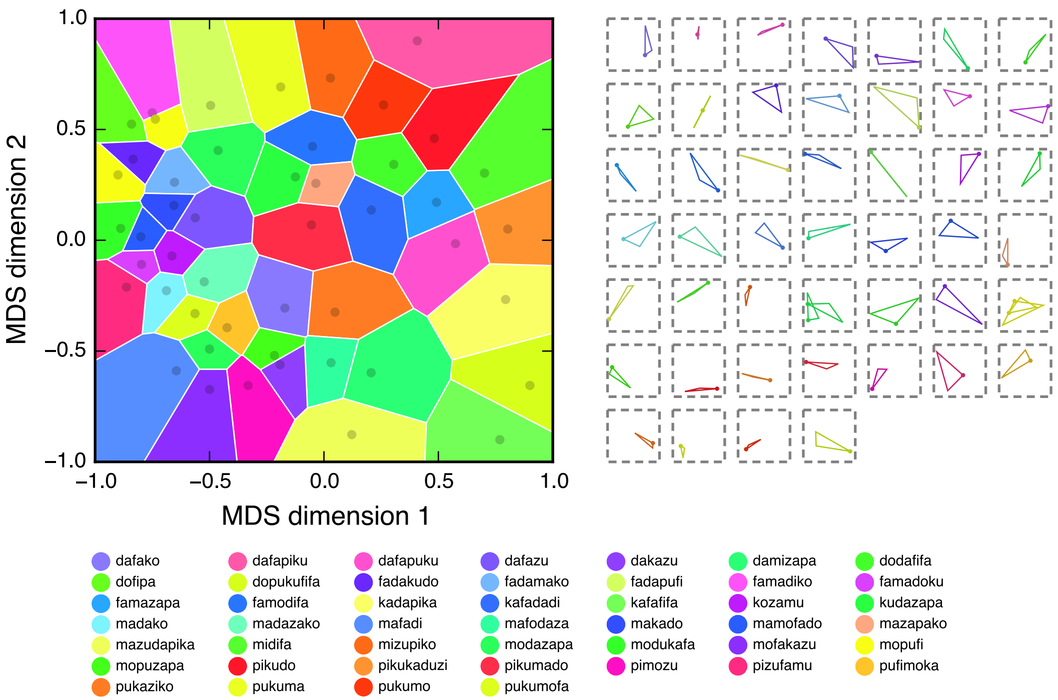

Experiment 3 Chain L Generation 1

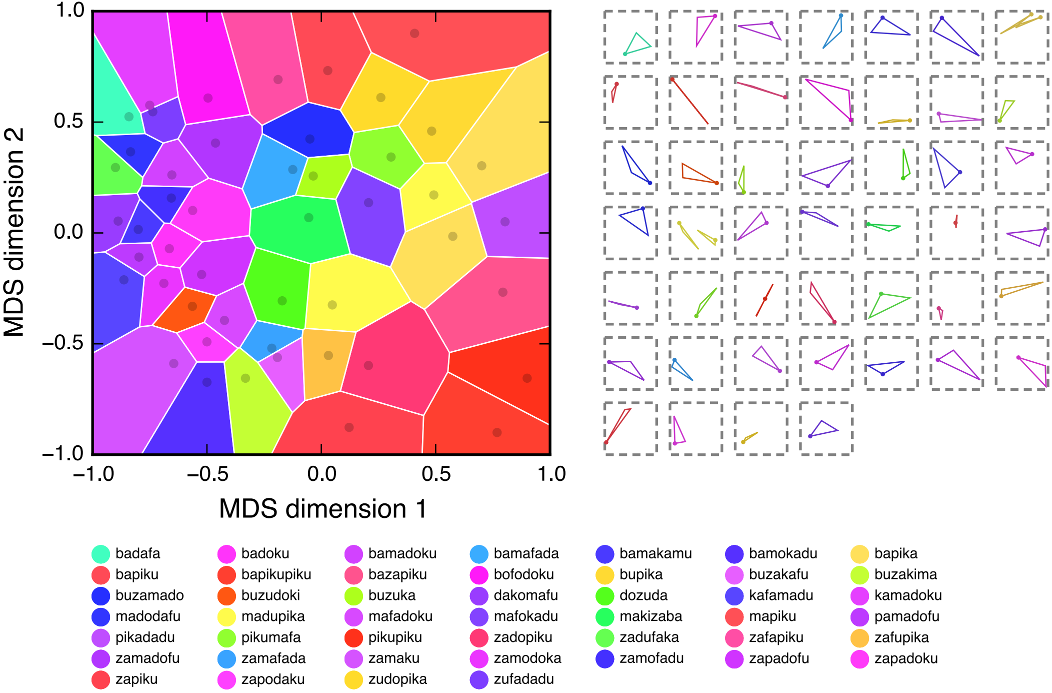

Experiment 3 Chain L Generation 2

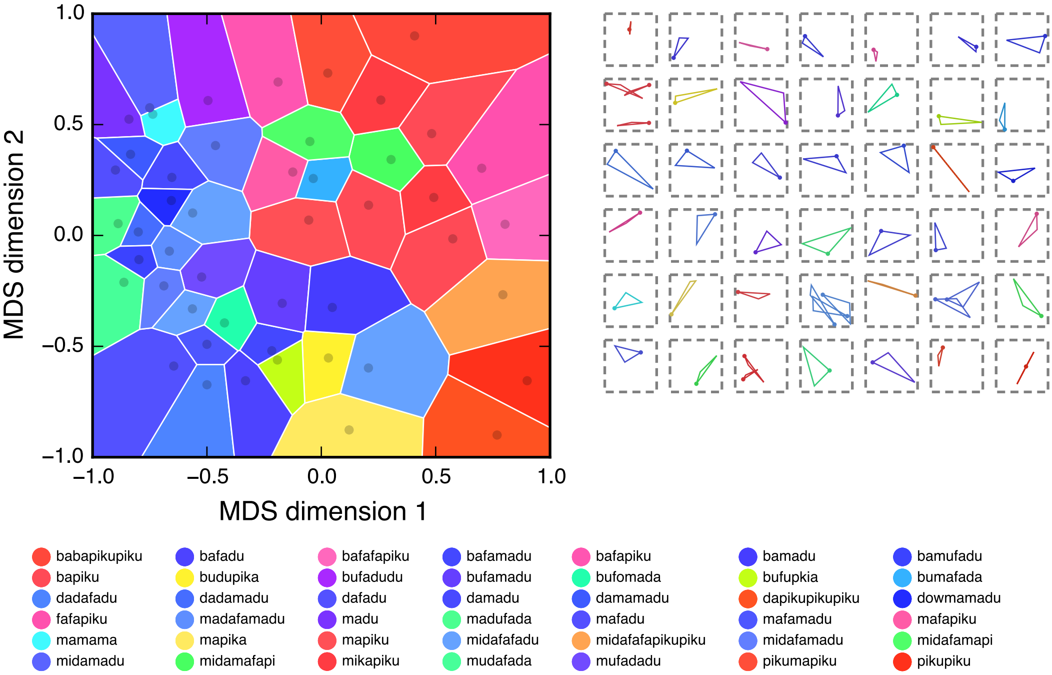

Experiment 3 Chain L Generation 3

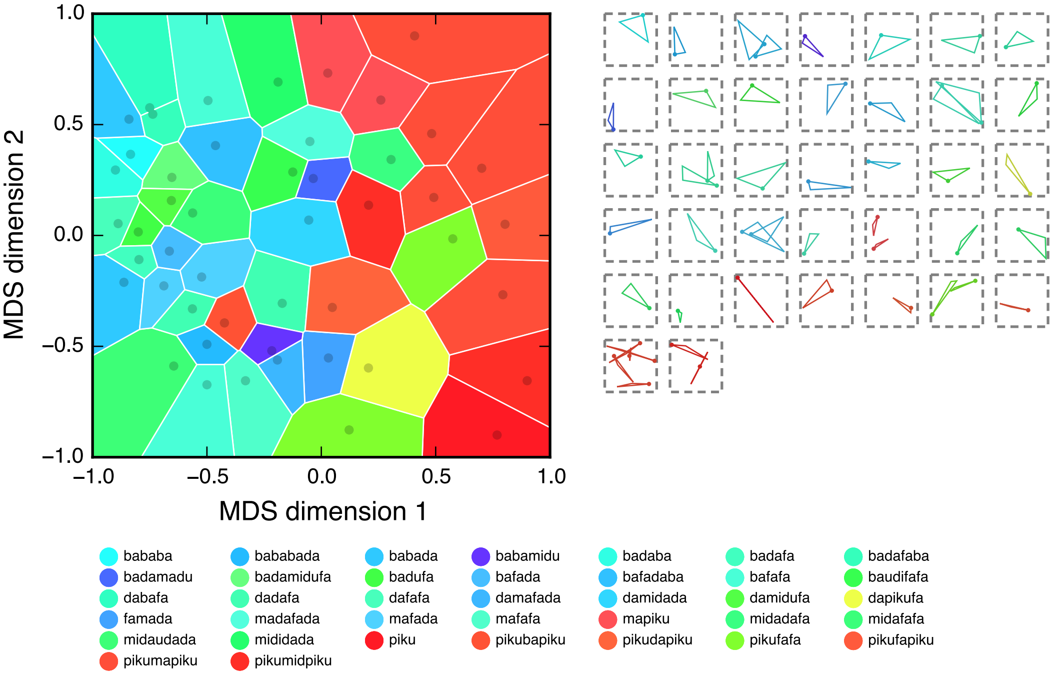

Experiment 3 Chain L Generation 4

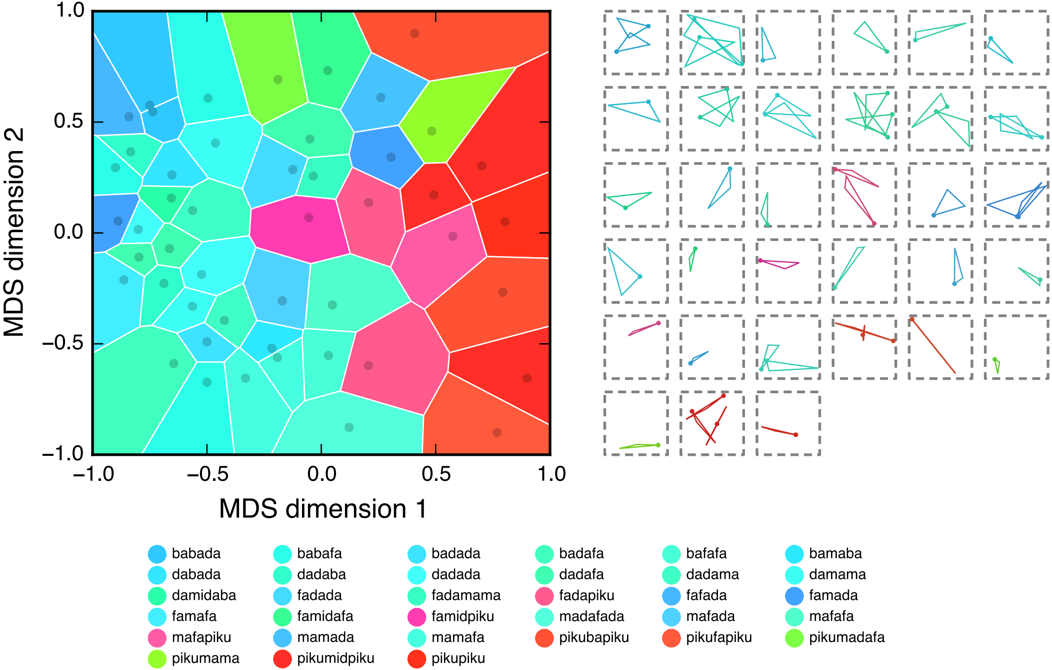

Experiment 3 Chain L Generation 5

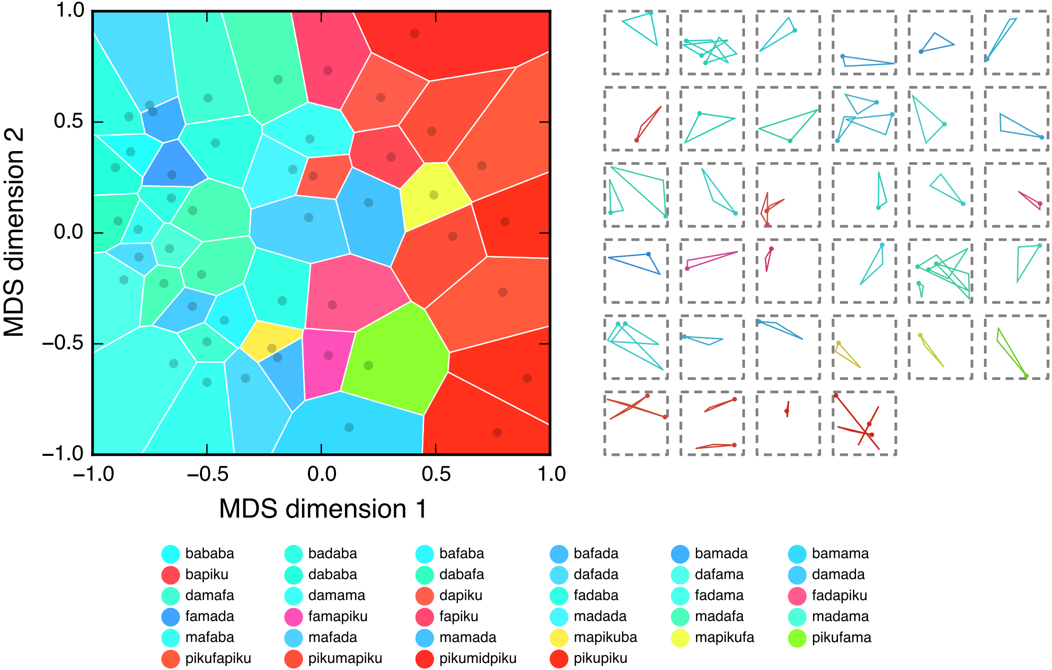

Experiment 3 Chain L Generation 6

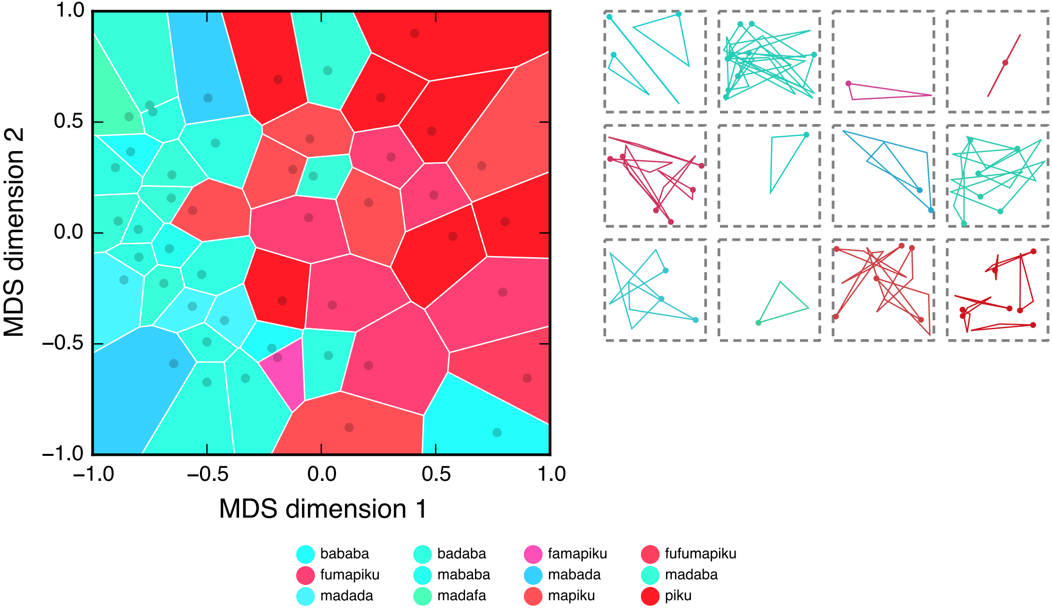

Experiment 3 Chain L Generation 7

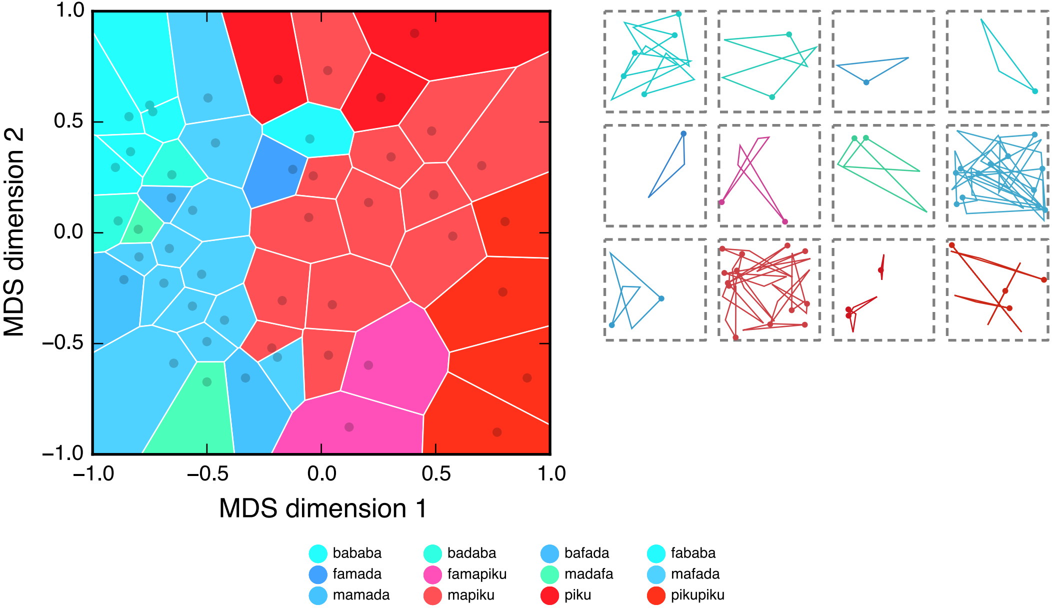

Experiment 3 Chain L Generation 8

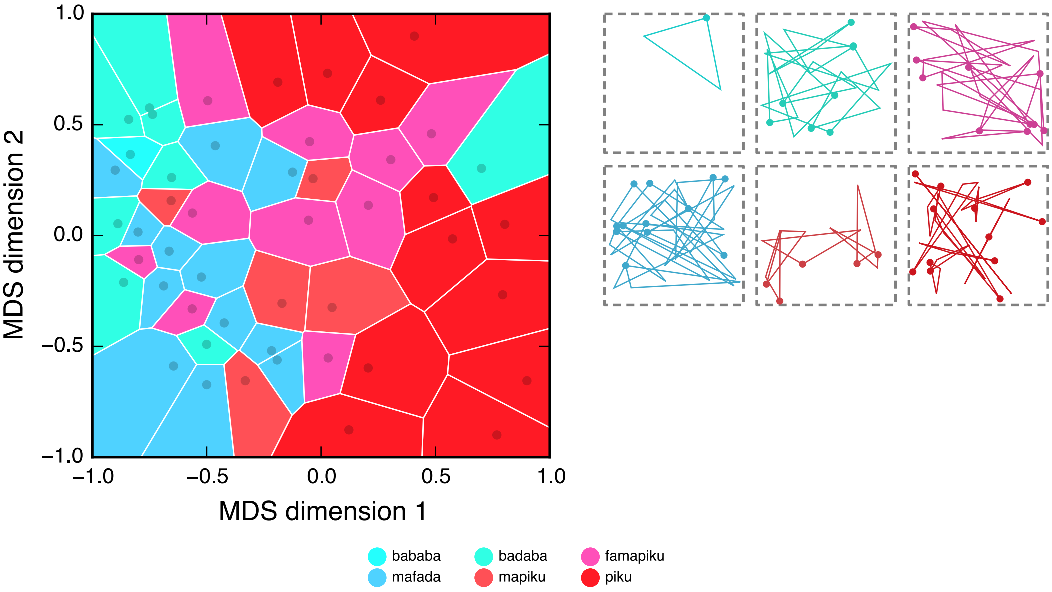

Experiment 3 Chain L Generation 9

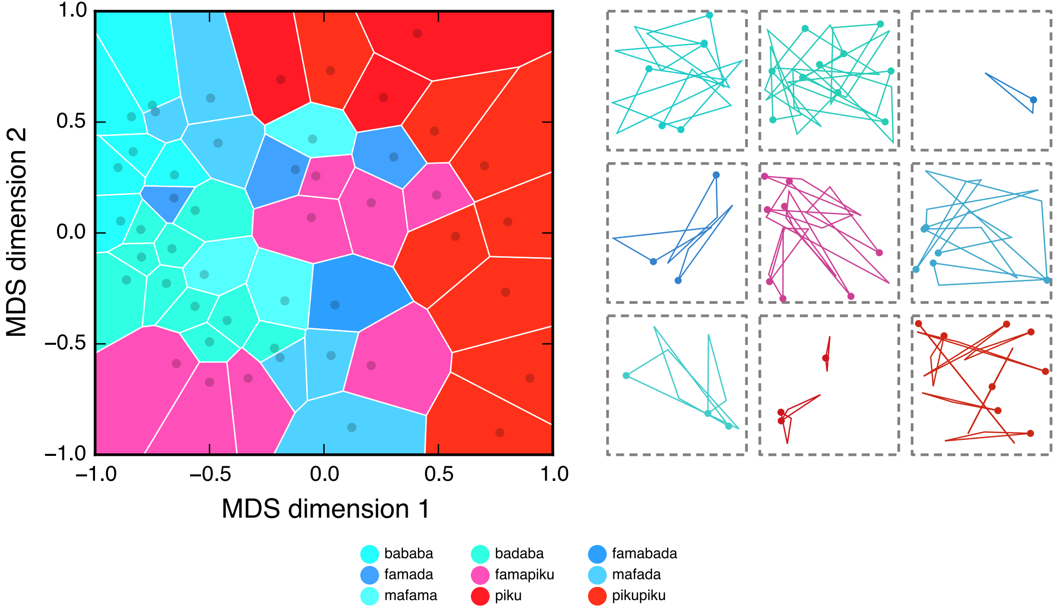

Experiment 3 Chain L Generation 10
